# Solution structure of the Sox2 DNA-binding domain reveals conformational selection in DNA binding

**DOI:** 10.1093/nar/gkaf1121

**Published:** 2025-11-04

**Authors:** Andrea Orsetti, Jonathan Slejfer, Satine Ha, Damian I Kevelam, Jan Tekkelenburg, Tjitske van Duijn, Anni Leppäkoski, Aren Sedrakyan, Ákos Szilagyi, Raymond D Schellevis, Abdenour Soufi, Vlad Cojocaru, Hugo van Ingen

**Affiliations:** NMR Spectroscopy Group, Bijvoet Centre for Biomolecular Research, Utrecht University, Utrecht, CH 3854, The Netherlands; NMR Spectroscopy Group, Bijvoet Centre for Biomolecular Research, Utrecht University, Utrecht, CH 3854, The Netherlands; NMR Spectroscopy Group, Bijvoet Centre for Biomolecular Research, Utrecht University, Utrecht, CH 3854, The Netherlands; NMR Spectroscopy Group, Bijvoet Centre for Biomolecular Research, Utrecht University, Utrecht, CH 3854, The Netherlands; NMR Spectroscopy Group, Bijvoet Centre for Biomolecular Research, Utrecht University, Utrecht, CH 3854, The Netherlands; NMR Spectroscopy Group, Bijvoet Centre for Biomolecular Research, Utrecht University, Utrecht, CH 3854, The Netherlands; NMR Spectroscopy Group, Bijvoet Centre for Biomolecular Research, Utrecht University, Utrecht, CH 3854, The Netherlands; NMR Spectroscopy Group, Bijvoet Centre for Biomolecular Research, Utrecht University, Utrecht, CH 3854, The Netherlands; STAR-UBB Institute & Doctoral School for Integrative Biology, Babeș-Bolyai University, Cluj-Napoca, 400084, România; NMR Spectroscopy Group, Bijvoet Centre for Biomolecular Research, Utrecht University, Utrecht, CH 3854, The Netherlands; Centre for Regenerative Medicine, Institute for Regeneration and Repair, The University of Edinburgh, Edinburgh, EH16 4UU, United Kingdom; NMR Spectroscopy Group, Bijvoet Centre for Biomolecular Research, Utrecht University, Utrecht, CH 3854, The Netherlands; STAR-UBB Institute & Doctoral School for Integrative Biology, Babeș-Bolyai University, Cluj-Napoca, 400084, România; NMR Spectroscopy Group, Bijvoet Centre for Biomolecular Research, Utrecht University, Utrecht, CH 3854, The Netherlands

## Abstract

The transcription factor Sox2 is a master regulator of cell pluripotency. While structural studies have provided insights into its DNA-bound conformation, the mechanisms governing its free-state conformational dynamics and DNA recognition remain elusive. Based on solution NMR spectroscopy and supported by molecular dynamics simulations, we here report the solution structure of the Sox2 DNA-binding domain (DBD), revealing that its helical core is well-structured and arranged as in its DNA-bound state. The folded, free protein coexists in dynamic equilibrium with partially unfolded states, which are quenched upon specific DNA binding. We show that the electrostatic environment significantly influences the Sox2–DBD stability, with high ionic strength stabilizing the protein. NMR titration experiments demonstrate that the nonspecific and specific DNA binding interfaces of Sox2 largely overlap. Specific binding, however, uniquely involves rigidification of part of the C-terminal tail. Based on these findings, we propose that the helical core of the Sox2–DBD is stabilized in its DNA-bound form prior to binding. Binding of Sox2 to DNA thus involves conformational selection, rather than exclusively induced fit, as was previously proposed. Through its pre-folded, DNA-binding competent fold, Sox2 may be able to rapidly switch from scanning of DNA to specific binding of its cognate site.

## Graphical Abstract

**Graphical Abstract:**
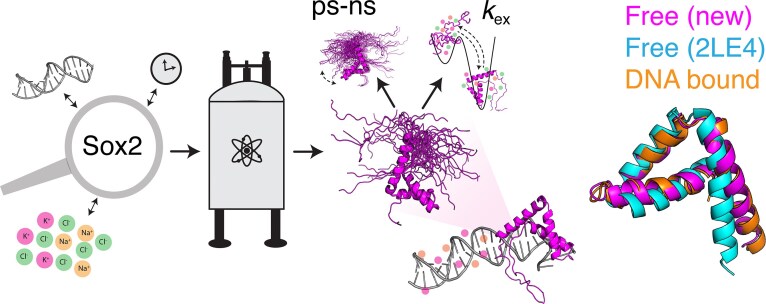

## Introduction

Transcription factors (TFs) are key regulators of gene expression and represent ∼1600 proteins of the human cell proteome [1]. Structurally, TFs share a common architecture, featured by a positively charged DNA-binding domain (DBD) and long intrinsically disordered regions (IDRs) at the N- and C-terminal domain [1, 2]. The DBD enables the TF to interact with its specific DNA-binding motif, while the IDRs contain transactivation domains, which recruit additional co-factors and components of the transcriptional machinery, ultimately triggering gene expression [1, 3, 4]. Binding of TFs to their target DNA is the critical first step of this cascade of events [3]. A thorough understanding of TF function requires a complete description of the molecular mechanism of DNA binding, including the structural transitions in protein and/or DNA, the binding affinities, kinetics, and specificity.

The DNA-binding mechanism is of particular interest for a special class of TFs that can bind chromatinized DNA to activate silenced genes, so called pioneer TFs (pTFs) [5–7]. These factors are able to bind to naked as well as nucleosomal DNA, placing additional constraints on their DBDs to cope with the partial occlusion of DNA in the nucleosome. Comparison of the DBDs of pTFs and non-pTFs has indicated that pTFs can adopt their DNA binding mode to engage only one face of the DNA helix [6, 8]. In addition, analysis of DNA target motifs bound by pTFs indicated a higher degree of divergence from the canonical binding motif (i.e. binding to “weak” motifs) when the pTFs bind nucleosomal DNA [8, 9]. Accordingly, recent cryo-EM structures of pTFs bound to nucleosomes have shown in some cases multiple pTFs bound to a nucleosome, including to “weak” motifs [10]. To what extent the DBDs of pTFs have special properties that provide them with required DNA-binding adaptability is not understood.

A quintessential pTF is Sox2, a member of the Sox (SRY-related HMG-box) protein family composed by 20 members found across the animal kingdom, which play key roles in regulating essential biological processes such as maintaining stem cell pluripotency, determining sex, enabling self-renewal, and guiding tissue development [11, 12]. Sox2 is crucial for both establishing and maintaining pluripotency during embryonic development [13, 14]. Beyond its key contribution to early development, Sox2 is involved in the maintenance and differentiation of proliferating neural progenitor cells (NPCs), thereby guiding the formation of the nervous system [15, 16]. Furthermore, its dysregulation has been implicated in cancer initiation and progression, where it contributes to tumor heterogeneity and stemness [17, 18]. Alongside these physiological and pathological roles, Sox2 is, together with Oct4, Myc and Klf4, the so called Yamanaka factors used to reprogram somatic cells into induced pluripotent stem cells (iPSCs) [19].

As a pTF, Sox2 can interact with enhancers localized in closed chromatin regions, promoting chromatin opening and genome accessibility [9, 20, 21]. Recent structural evidence indicates that Sox2 interplays with chromatin by binding nucleosomes in different superhelical locations, suggesting that binding is then followed by bending and unravelling of the DNA from the nucleosome complex [22, 23]. A prevailing idea is that Sox2–DBD is highly dynamic in its free state and that this property endows the DBD with required versatility to adapt the DNA-binding mode to different chromatin and sequence contexts [6, 23].

Like all Sox proteins, Sox2 features a conserved DBD belonging to the HMG (high-mobility group) superfamily, flanked by a short N-terminal IDR and long C-terminal IDR containing the transactivation domain (TAD) [11, 24]. The HMG domain consists of three α-helical bundles arranged in an L-shaped structure that upon binding to the DNA minor groove cause DNA bending [25, 26]. The Sox2 HMG binds a TTGT core motif, inducing a characteristic DNA bending at angles between 50 and 85 degrees [27, 28]. Residue M49 within helix α1 of the HMG intercalates (^) in the T^TGT localized in the minor groove leading to bending and opening of the DNA strands [28]. Comparison with recent cryo-EM structures indicates that this DNA-binding mode is maintained at exposed TTGT sites in the nucleosome [22, 29]. A detailed description of the free-state Sox2 HMG structural properties is however lacking.

The PDB contains an unpublished solution structure of the free Sox2 HMG domain (PDB: 2LE4), in which the relative orientation of the three helices is significantly different compared to the DNA-bound state. This is suggestive of a pronounced structural transition within the Sox2 HMG upon DNA binding, in line with a “floppy Sox” model in which the Sox HMG domains undergo a disorder-to-order transition upon binding to their cognate DNA, a process that involves mutual induced fit [30]. Analysis of Sox2 orthologue structures in free and bound state presents conflicting evidence for such structural changes [30–34]. Thus, extrapolation of these data to Sox2 remains highly tentative.

Here, we used solution NMR spectroscopy, thermal stability assays, and molecular dynamics (MD) simulations to determine the structure, dynamics and stability of an extended human Sox2 HMG domain. The Sox2 HMG domain solution structure shows a three-helical bundle conformation identical to its DNA-bound state, indicating that folding is already encoded in the free state. The domain has limited thermostability due to high electrostatic frustration, resulting in a dynamic equilibrium between the folded HMG and a minor population of partially unfolded states. These partially unfolded states are quenched upon specific DNA binding. Interestingly, high electrostatic environments can mimic the stabilization of Sox2 HMG observed when bound to specific DNA, biasing Sox2 toward its folded state. Furthermore, we found that during DNA titration, the initial binding at high Sox2/DNA ratios is highly similar for specific and nonspecific DNA, involving the large same Sox2 interface. In contrast, at 1:1 Sox2/DNA ratio, the binding mode to specific DNA is clearly distinct from random DNA binding, indicative of a smooth transition from nonspecific to specific binding. The fully bound state stabilizes the Sox2 HMG and rigidifies part of the C-terminal IDR. These findings support a conformational selection model in which the DNA-binding interface is pre-formed in the free-state Sox2 HMG domain, priming it for specific DNA interactions.

## Materials and methods

### Protein expression and purification

An extended version of the human Sox2–DBD was designed based on the UniProt entry P48431, encompassing amino acids 31–127 (henceforth Sox2^31–127^) with a theoretical molecular weight of 11.74 kDa (Swiss Prot). The sequence was optimized for *Escherichia coli* (*E. coli*) expression and cloned into a pET24(+) plasmid containing an N-terminal (6x)-Histidine (His) tag and a thrombin cleavage site. The plasmid was synthesized by Twist Bioscience. One Shot^™^ BL21 Star^™^ (DE3) cells were transformed with 50 ng of the pET24(+) plasmid encoding the 6xHis Sox2^31–127^. Transformed cells were cultured in 500 ml of Luria-Bertani (LB) medium containing 100 mg/ml ampicillin at 37°C with shaking at 200 rpm. Upon reaching an optical density at 600 nm (OD_600_) of 0.6, the cultures were cooled to 20°C and induced with 0.5 mM isopropyl β-d-1-thiogalactopyranoside (IPTG) for 16–18 h at 20°C, shaking at 180 rpm. For producing isotope labelled Sox2^31–127^ a 10 ml of LB pre-culture was grown for 8 h, followed by overnight inoculation into 50 ml of unlabeled M9 medium. Subsequently, 500 ml of labeled M9 medium (either with ^15^NH_4_Cl or ^15^N NH_4_Cl -^13^C-d-glucose) was inoculated with the adapted pre-culture, to a starting OD_600_ of 0.2. Cultures were then grown and induced following the same protocol as for the unlabeled protein. Cells were harvested by centrifugation at 4667 × *g* for 20 min at 4°C. The pellets were resuspended in 30 ml of binding buffer (20 mM NaH_2_PO_4_, 0.5 M NaCl, 20 mM imidazole, and 1 mM DTT, pH 7.4) and frozen at −80°C. Following one freeze–thaw cycle, protease inhibitors (cOmplete^™^ EDTA-free Protease Inhibitor Cocktail, Sigma–Aldrich), 1 mg/ml bovine lysozyme (Sigma–Aldrich), and 30 U/ml DNase I (Sigma–Aldrich) were added, followed by sonication in ice (Fisherbrand^™^ 505 Sonicator) for five cycles of 40 s (10 s on, 30 s off). After that the lysates were incubated at 37°C for 1 h to maximize DNase I activity. The lysates were ultracentrifuged at 37 156 × *g* for 40 min at 4°C.

The Sox2^31–127^ protein (unlabeled and isotope labeled) was purified using an ÄKTA pure^™^ chromatography system (Cytiva) using His-tag affinity chromatography followed by size-exclusion chromatography (Supplementary Fig. S1). For affinity chromatography, the supernatant was loaded onto a 5 ml HisTrap HP column (GE Healthcare) pre-equilibrated with 5 column volumes (CV) of binding buffer, followed by a wash with 10 CV of binding buffer. The protein was eluted using a 10 CV gradient with elution buffer (20 mM NaH_2_PO_4_, 0.5 M NaCl, 500 mM imidazole, and 1 mM Dithiothreitol (DTT), pH 7.4). Fractions containing the target protein were pooled and dialyzed overnight (16–18 h) at 4°C in a buffer (20 mM NaH_2_PO_4_, 0.5 M NaCl, and 1 mM DTT, pH 7.4) with 10 U/mg of bovine thrombin (Sigma–Aldrich) to cleave the His-tag. The dialyzed protein was concentrated to 5 ml using Amicon Ultra-50 centrifugal filters (3 kDa MWCO) and loaded onto a HiLoad 26/600 Superdex 75 pg (GE Healthcare) column pre-equilibrated with physiological-like buffer (135 mM KCl, 15 mM NaCl, 20 mM Tris, and 1 mM DTT, pH 7.3). The protein was purified via isocratic elution, and fractions containing the Sox2^31–127^protein were pooled, concentrated, and shocked-frozen for storage at −80°C.

### Production double-strand DNA

To perform DNA-binding studies, two complementary, 13-mer DNA oligonucleotides containing a canonical Sox2-binding site derived from the *FGF4* enhancer (henceforth FGF4) (5′-ACTCT**TTGT**TCGA-3′, 5′-TCGA**ACAA**AGAGT-3′) [28, 35], as well as a random DNA sequence lacking the TTGT motif but with same GC content (5′-ACTCTCTAGGATA-3′) and its reverse complement were obtained from Eurofins. To produce double-stranded DNA (dsDNA) the two complementary oligos were solubilized in physiological buffer (135 mM KCl, 15 mM NaCl, 20 mM Tris, and 1 mM DTT, pH 7.3) to a final concentration of 5 nmol at 1:1 molar ratio and annealed by heating to 90°C for 10 min and slowly cooling down to 10°C. The dsDNA formation was confirmed by 1D-NMR at 20°C from the appearance of clear imino proton signals in the region 12–14 ppm (Supplementary Fig. S2).

### Thermostability measurements

The thermostability of Sox2^31–127^ was determined using intrinsic fluorescence by NanoDSF equipped in a Prometheus Panta instrument (NanoTemper Technologies). A total of 18 different buffer conditions, varying in pH, salt concentration, and buffering agents, were used for screening (Table S1). Conditions 1–7 were adopted from previous studies reported in the literature while condition 8 was formulated based on insights from existing literature. Condition 9.1 represents the physiological-like buffer, while conditions 9.2 to 9.10 involve variations in salt concentration and pH of the physiological-like buffer. Buffer 9.1 was used to compare the thermal unfolding of free versus DNA bound Sox2^31–127^.

For the NanoDSF measurements, an initial stock solution of 200 µM Sox2^31–127^ was diluted with physiological-like buffer to a final concentration of 25 µM across all the 18 conditions and the DNA bound state. For DNA-bound samples, FGF4 DNA as added to a 1:1 molar ratio. All samples were pre-incubated at room temperature for 30 min, then loaded into 10 µl high-sensitivity capillaries (PR-C006) and analyzed in duplicates. The measurement was performed applying a heating ramp rate of 1 K/min ranging from 288 to 368 K. Melting curves were generated for each sample and fit to a sigmoidal curve to determine the melting temperature (*T*_m_) using the analysis software of Prometheus Panta. Uncertainty in the extracted *T*_m_ was set to 0.3 K (higher than the variability in the technical replicates) for all reported conditions based on independent repeat experiments on free Sox2^31–127^ in physiological-like buffer. In addition, the melting curves for free, FGF4 or random-DNA bound Sox2^31–127^ and free Sox2^31–127^ in 2 M KCl were fitted using an in-house MATLAB (The Mathworks) script, following the approach of Lindorff–Larson and Teilum [36] and as implemented in CDpal [37] (Supplementary Fig. S3). Briefly, the fluorescence (*f*) at temperature *T* is given by:


\begin{eqnarray*}
f\left( T \right) &=& \left\{ {(aN0\ + \ gN \cdot \left( {T - {{T}_r}} \right)+ \ (aU0\ + g1U } \right.\\ &&\ \ \left.{\cdot \left( {T - {{T}_r}} \right)\ + g2U \cdot {{{\left( {T - {{T}_r}} \right)}}^2}} \right\}\frac{{{{e}^{ - {\mathrm{\Delta }}G/RT}}}}{{1 + {{e}^{ - {\mathrm{\Delta }}G/RT}}}}
\end{eqnarray*}

and


**Figure 1.**
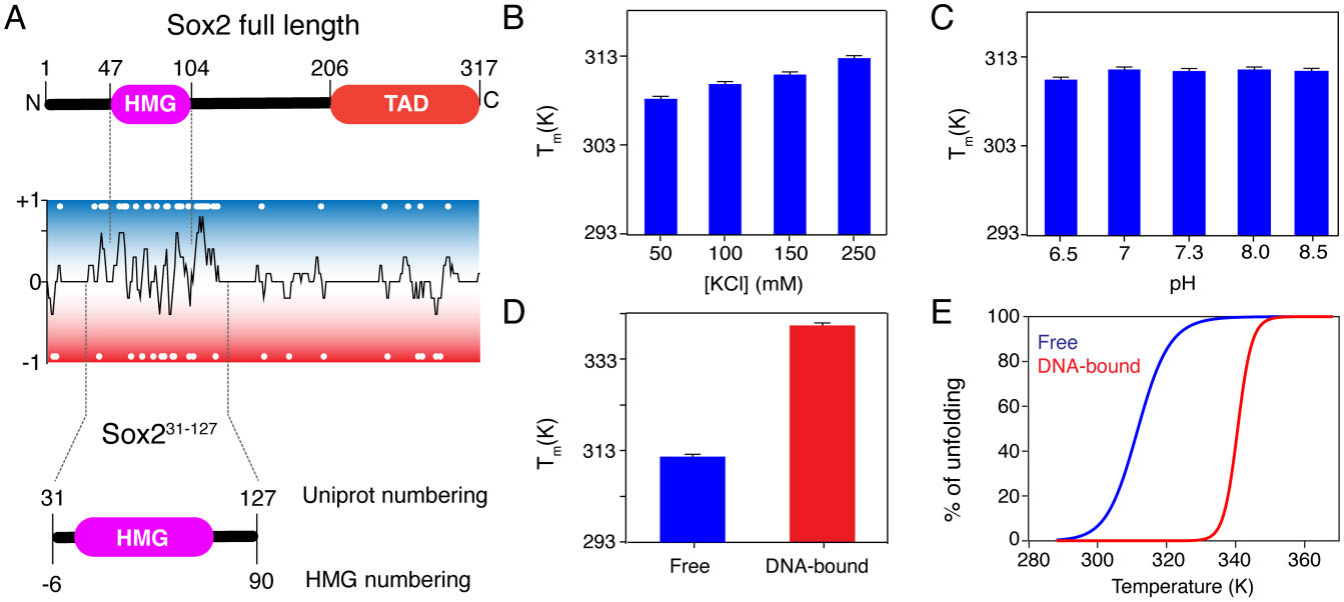
Sox2–DBD construct design and thermostability. (**A**) Domain architecture of full-length *Hs*. Sox2. Sox2 (top), charge distribution (5-residue window average, middle) and schematic of the Sox2^31–127^ construct used in this study (bottom). Positions of charged residues indicated as white dots. UniProt and HMG numbering are indicated for Sox2^31–127^. (**B** and **C**) Melting temperatures of free Sox2^31–127^ as a function of KCl concentration in 20 mM Tris, pH 7.3 (**B**) and as a function of pH at 150 mM ionic strength. (**C**) Data represent mean ± SD from *n* = 2. (**D** and **E**) Comparison of melting temperatures (D) and fraction unfolded as function of temperature (E) of free and FGF4 DNA-bound Sox2^31–127^ in physiological-like buffer. Data represent mean ± SD from *n* = 2.

\begin{eqnarray*}
{\mathrm{\Delta }}G &=& {\mathrm{\Delta }}H - T \cdot {\mathrm{\Delta }}S = {\mathrm{\Delta }}{{H}_m} + {\mathrm{\Delta }}{{C}_p} \cdot \left( {T - {{T}_m}} \right)\\&&- {\mathrm{\Delta }}{{C}_p} \cdot T \cdot ln\left( {T/{{T}_m}} \right)\ + {\mathrm{\Delta }}{{H}_m} \cdot T/{{T}_m}
\end{eqnarray*}

where *T*_m_ is the melting temperature; *T*_r_ is the reference temperature; aN0, gN, g1U, aU0, and g2U are baseline parameters, and Δ*H*_m_ is the enthalpy of unfolding; Δ*C*_p_ is the change in heat capacity of unfolding. The fits were used to calculate the fraction unfolded protein as function of temperature as shown in main text Fig. 1E.

### NMR spectroscopy

NMR experiments were conducted on Bruker spectrometers operating at 1200 (NEO), 900 (NEO), 850 (Avance III), and 600 MHz (Avance-III cryoprobe) ^1^H Larmor frequency. All were equipped with TCI or Prodigy (600) 5 mm cryoprobes. Experiments were conducted in 5 mm or 3 mm tubes at 293 K using samples containing 0.12–0.25 mM ^15^N or ^15^N,^13^C uniformly labeled Sox2^31–127^ in NMR buffer (20 mM Tris, 135 mM KCl, and 15 mM NaCl, pH 7.3) with 10% D_2_O and 0.01% NaN_3_. Spectra were processed using Bruker TopSpin 4 or NMRPipe [38]. Chemical shifts were referenced to 2,2-dimethyl-2-silapentane-5-sulfonate (DSS) [39].

*Backbone and side chain assignment*. Backbone assignments of N, H_N_, C_α_, C_β_, C’, H_α_, and H_β_ were derived using standard triple resonance approach based on 2D ^15^N-TROSY, ^15^N-HSQC, 3D HNCA, HNCOCA, HNCO, HNCACO, HNCACB, CBCACONH, and HBHACONH experiments. Backbone assignment was performed manually using POKY [40] and 96.8% complete. Assignments were missing for the terminal GS thrombin cleavage scar and the backbone amides of Sox2 residues N33, N68, S69, and R114, likely due to solvent exchange.

Assignment of aliphatic side chain resonances was based on 3D HCH-TOCSY and CCH-TOCSY. Aromatic side chains were assigned using CBHD and CBHE spectra. Stereo-specific assignments of the Val γ1/γ2 and Leu δ1/δ2 pro-chiral methyl groups were obtained using the approach of Neri *et al.* on a 10% ^13^C-labeled sample [41]. In addition, a 3D ^15^N-edited NOESY and a 2D ^1^H-^1^H-NOESY spectra were recorded at 1200 MHz and a broadband [42] (^13^C-edited NOESY at 850 MHz, all with NOE mixing times set to 120 ms, to aid assignment and to derive distance restraints for structure calculation).

Cross-peaks in TOCSY and NOESY spectra were picked automatically in POKY [40]. Assignments of side chains resonances and NOESY cross peak assignments were determined in a semi-automated, iterative manner using the FLYA algorithm in CYANA v3.98.15 [43] using 3D CCH, HCH TOCSY spectra, and the NOESY spectra together with the manually obtained backbone chemical shifts assignments (N, H_N_, C_α_, C_β_, C’, H_α_, and H_β_) and assignment of aromatic side chain resonances (based on CBHD and CBHE spectra) as input. In addition to the default exclusion of Arg NH_2_ and Lys NH_3_ resonances, hydroxyl protons of Ser, Thr were excluded from assignment. Peak tolerances were set to 0.3 ppm for ^15^N and ^13^C and 0.025 for ^1^H. FLYA assignment were manually validated, in particular for aromatic side chain, Met methyl, and Asn/Gln side chain resonances, which were manually refined and expanded based on initial results from FLYA using NOESY data. NOESY peak lists were refined manually based on visual inspection of unassigned and violating NOEs in initial structure calculation runs. All proline residues were determined to be in trans conformation based on C_β_/Cγ chemical shift difference. In the final FLYA run, in total 1055 strong assignment were obtained, which were manually curated to 1040 validated assignments (91.5% of all resonances), excluding a number of side-chain assignments for the flexible C-terminal residues. In this final FLYA run, all convergence criteria (target function value, % unassigned NOEs and RMSD) were met.

*Residual dipolar couplings (RDC) measurement*. Backbone amide residual dipolar couplings (NH-RDCs) were measured using a weak alignment medium. The alignment condition was optimized with pentaethylene glycol monodecyl ether in 50% (w/v) aqueous solution (Affymetrix) (henceforth C_10_E_5_) at various concentrations as described in the literature [44]. The optimal alignment condition was determined to be 5% C_10_E_5_/*n*-hexanol, based on the evaluation over time of deuterium-hydrogen splitting stability and line shape conservation. The sample preparation was conducted at room temperature. A 10% C_10_E_5_ stock solution in physiological buffer was prepared, and 250 µl of this solution were titrated with *n*-hexanol (Sigma–Aldrich) in 1 µl increments, vortexing after each addition. The solution turned milky upon the addition of *n*-hexanol, and *n*-hexanol was added until the solution became clear again, indicating the successful formation of a liquid crystal phase. Subsequently, 210 µl of 0.25 mM Sox2^31–127^ were mixed with 10 µl of D_2_O, 5 µl of 1% NaN_3_, and 10 µl of a 50× protease inhibitor cocktail. This mixture was added to the 250 µl of 10% C_10_E_5_/*n*-hexanol stock solution in 5 µl increments. The final solution contained 125 µM Sox2^31–127^, 5% C_10_E_5_/*n*-hexanol_,_ 10% D_2_O, and 0.01% NaN_3_. NH-RDCs were measured at 293 K using the IPAP (in-phase/anti-phase) HSQC pulse sequence [45]. To determine the NH-RDCs, two spectra were acquired: one with Sox2^31–127^ alone (without C_10_E_5_/*n*-hexanol) and another with Sox2^31–127^ in 5% C_10_E_5_/*n*-hexanol. Spectra were recorded with ^1^H/^15^N acquisition times of 95 ms, processed with linear prediction in the ^15^N dimension and zero-filled to digital resolution of 2.64/0.95 Hz in the ^1^H/^15^N dimension. Peak position was determined in POKY using the “peak center” command. The NH-RDCs were calculated by subtracting the scalar couplings (*J*_NH_^app^) obtained from the aligned sample from those of the unaligned sample (*J*_NH_).

*^15^N R_1_/R_2_, ^15^N{^1^H} NOE experiments*. Backbone amide ^15^N transverse and longitudinal relaxation (*R*_1_ and *R*_2_) and the steady-state heteronuclear Overhauser effect (*^15^N{^1^H}* NOE) were recorded for both free and DNA bound Sox2^31–127^ at 293K using a 600 MHz (^1^H frequency) equipped with a Prodigy cryoprobe using sensitivity-enhanced HSQC-based pulse sequences from the Bruker Topspin library. For the *R*_1_ experiment, the recycle delay was 2.5 s and the relaxation delays were set at 10, 100, 200, 300 (2×), 500, 1000, and 1500 ms. For the *R*_2_ experiment, the recycle delay was 2.5 s and the CPMG relaxation delays (with 90 μs 180° pulses spaced at 1.98 ms, corresponding to a CPMG frequency of 505 Hz) relaxation delays were 15.82, 31.64, 47.46, 63.28, 79.1, 94.92, 110.74 (2×), 126.56, 142.38, 158.2, 174.02, and 189.84 ms. The delays were recorded in interleaved manner. The *^15^N{^1^H}* NOE was recorded with a recycle delay of 10 s with (*I*_sat_) and without (*I*_0_) proton saturation. The *R*_1_, *R*_2_, and *{^1^H}-^15^N* NOE relaxation data were analyzed using CCPN Analysis 3.1.1 [46] .The *R*_1_ and *R*_2_ relaxation rate constants were determined by fitting the intensity decay monoexponentially as a function of relaxation delays. The *^15^N{^1^H}* NOE values were calculated from the ratio of peak heights between saturated (*I*_sat_) and unsaturated (*I*_0_) spectra. For both the free and DNA bound state, *R*_1_ and *R*_2_ and NOE values were determined for 72 of the 85 nonproline assigned residues. Residues with resonance overlap or low signal-to-noise ratios were excluded from analysis.

*Carr–Purcell–Meiboom–Gill (CPMG) relaxation dispersion experiments*. Backbone amide TROSY ^15^N CPMG relaxation dispersion experiments were recorded for both free Sox2^31–127^ at 900 (with 5 mm TCI cryoprobe) and 1200 MHz (with 5 mm TXO cryoprobe) ^1^H Larmor frequency at 293 K using a pseudo-3D constant time TROSY-based pulse sequence [47]. Data for DNA-bound Sox2^31–127^ were recorded at 900 MHz. Spectra were recorded with recycle delay of 2 s, the constant-time relaxation delay (*T*_relax_) of 40/30 ms (900/1200 MHz), ^15^N 180° pulses during the CPMG pulse train of 86.6/76 μs (900/1200 MHz). At 900 MHz, ν_CPMG_ (ν_CPMG_ = 1/(4τ), where 2τ is the interpulse delay in the CPMG train) was set to 25, 50, 75, 100 (2×), 125 150, 200, 250, 300, 400, 500 (2×), 600, 700, 800 (2×), 1000, 1250, 1500, and 2000 Hz. At 1200 MHz, ν_CPMG_ was set to 33.3, 66.7 (2×), 100, 133.3, 166.7, 200, 233.3, 266.7, 333.3, 400 (2×), 466.7, 566.7, 666.7, 800 (2×), 900, 1000, 1500, and 2000 Hz. Spectra were processed using NMRPipe [38] using a Lorentz-to-Gauss window function. Peak intensities (*I*) were determined by line-shape fitting (GALORE) using PINT [48]. The effective *R*_2_ (*R*_2,eff_) was computed as *R*_2,eff_ = –1/*T*_relax_ ln (I(ν_CPMG_)/*I*_0_), where *I*_0_ is the peak intensity in a spectrum recorded without the relaxation delay *T*_relax_. Uncertainties were determined from the root-mean-square-deviation (RMSD) of the *R*_2,eff_ values derived from the duplicate measurements and set to be at least 0.3 s^–1^ or 2% of the *R*_2,eff_ value.

Signs of the chemical shift differences between ground and excited states (Δω_GE_) were determined using comparison of ^1^H–^15^N HSQC and HMQC spectra [49]. At 293 K, maximum peak position difference was ∼1 Hz, likely due to the low population of the excited state. The sign of Δω_GE_ could only be determined for few residues. Subsequent HSQC/HMQC comparisons at 299 and 301 K resulted in much larger differences in peak position, up to 5.5 and 9.5 Hz, respectively (see Supplementary Fig. S13). At 301 K, the HMQC spectrum suffers from extensive line broadening for many resonances. Ultimately, the sign of Δω_GE_ could be obtained for 17 residues based on the 299 K data.

*NMR titration experiments*. To monitor interaction with DNA, an NMR titration study was carried out at 293 K using a 900 MHz spectrometer. The FGF4 (5′-ACTCT**TTGT**TCGA-3′) and random DNA (5′-ACTCTCTAGGATA-3′) were added to samples of ^15^N–Sox2^31–127^ at 10%, 30%, 50%, 70%, 100%, and 120% molar equivalents. A ^15^N-TROSY spectrum was acquired for each titration point, and at the final titration point, HNCA, NHCACB, and CBCACONH experiments were performed to assign backbone NH resonances in the bound state.

Titration with KCl on free state ^15^N–Sox2^31–127^ were conducted in a 3 mm NMR tube with an initial concentration of 100 µM ^15^N–Sox2^31–127^ in buffer 9.7 (refer to Supplementary Table S1 for buffer composition). Salt additions were performed incrementally in the following concentrations: 0.25, 0.5, 1, 1.5, and 2 M. For the KCl titration for each titration point a ^15^N-TROSY (293 K, 900 MHz) experiment was recorded to monitor changes in chemical shifts.

Data analysis was performed using the POKY software to extract chemical shift values, resonance intensities and signal-to-noise ratios (S/N) were determined using POKY [40]. Chemical shift perturbation (CSP) mapping between 0% and 100% of both FGF4 and random DNA and 0.15 and 2 M KCl was calculated using the following formula, where Δ_H_ and Δ_N_ are the peak displacements in ppm along the ^1^H and ^15^N dimension, respectively:


\begin{eqnarray*}
CSP = \ \sqrt {\left( {\Delta _H^2 + \ {{{\left( {\Delta _N^{}/6} \right)}}^2}} \right)}
\end{eqnarray*}

### Structure calculation

Dihedral angle restraints for the ϕ and ψ angles were obtained from N, NH, C’, Cα, Cβ, and Hα chemical shifts using TALOS-N [50]. Using the obtained chemical shift assignments as input, automatic assignment of NOESY cross peaks and structure calculation were run 10 times with different seeds and tolerances of 0.3 ppm for ^13^C,^15^N, and 0.03 ppm for ^1^H. NOESY cross peak assignments were kept only if a consistent assignment was obtained in seven or more runs, resulting in 1577 NOEs of which 245 long-range. Stereospecific assignments made by CYANA were kept only if reproduced in nine or more out ten runs. The ensemble of 20 structures calculated from this final set of distance restraints and the backbone dihedral angle restraints was subsequently used to determine the magnitude and rhombicity of the alignment tensor by singular-value decomposition using 43 experimental backbone N-H RDCs. In the final structure calculation, the distance, dihedral angle and RDC restraints were used to calculate 100 structures using the NOE/RDC script of CYANA with weight of RDCs increased from 0.02 to 0.03. The 20 structures with lowest target function value were selected to represent the final structural ensemble, structural statistics are shown in Table 1. Structure quality was assessed using PALES [51], PROCHECK [52] and the Protein Structure Validation Suite (PSVS) webserver (https://montelionelab.chem.rpi.edu/PSVS/PSVS/). The classification of the protein loops has been performed using the ArchDB classification system [53].

**Table 1.** Structural statistics for free-state Sox2^31–127^

*A. Restraint information*	
Total number of distance restraints	1577
Intra-residual/sequential/medium/long	454/423/455/245
Total number of RDC restraints	43
	97/42/16/0
Total number of backbone dihedral angle restraints *f/yq*	57/60
*B. Average deviation from experimental restraints*	
RMS experimental distance restraints (Å)	0.0090 ± 0.0010
Average number of distance violations > 0.5 Å	0
RMS experimental dihedral angle restraints (°)	0.249 ± 0.055
Average number of dihedral angle violations > 5°	0
RMS experimental RDC restraints (Hz)	0.79 ± 0.02
RDC *Q*-factor	8.07 ± 0.21
*C. Coordinate RMS deviation (Å)*	
*Average overall RMSD to mean structure* ^a^	
Ordered heavy backbone atoms	0.46 ± 0.19
Ordered all heavy atoms	0.86 ± 0.13
Global backbone atoms	9.58 ± 3.81
	1.64
Global all heavy atoms	9.84 ± 3.71
	2.25
*D. Ramachandran plot quality parameters (%)* ^*^a^*^	
Residues in most favored regions (ordered/global)	97.8 / 77.0
	83.0 /± 4.5
Residues in allowed regions (ordered/global)	2.2 / 19.0
	13.5 ± 4.0
Residues in additionally allowed regions (ordered/global)	0.0 / 2.6
	2.3 ± 2.1
Residues in disallowed regions (ordered/global)	0.0 / 1.4
	1.1 ± 1.1
*E. Abnormalities found in structural checks*	
Abnormally short interatomic distances /1000 atoms	4.24
	10 ± 2

a Statistics are given for residues 31–127. Ordered regions are residues 45–103.

An independent calculation using ARTINA [54] on the NMRtist platform [55], starting from the complete set of raw, unpicked spectra and without any starting assignments, resulted in a well-defined structure with a reported 1.1 Å structure accuracy metric. Assignments were reported to have 79% accuracy, flagging the aromatic assignments as low confidence. The ARTINA structure yielded the same relative orientation of the three helices (heavy backbone RMSD 1.31 Å for residues 47–104 corresponding to the well-folded HMG core). Comparison of ARTINA and manually curated assignments showed that ARTINA generates ~10% (114) more assignments that largely correspond to highly overlapping resonances from the flexible tails. These assignments were removed in the manual curation step. Overall ∼80% of the assignments (including all HMG core backbone assignments) agreed, while for ∼20% there was a chemical shift difference >0.2 ppm (^13^C,^15^N) or 0.02 ppm (^1^H) between the ARTINA and manually curated assignment. Most of these corresponded to resonances belonging to the flexible tails where ARTINA typically assigned multiple resonances to the same chemical shift. More consequential for the structure were several assignment swaps and overall lower assignment completeness for the aromatic side chain resonances, which resulted in a different packing of the aromatic residues in the HMG core compared to that in the manually curated structure and the crystal structure of DNA-bound Sox2 [28] (Supplementary Fig. S8).

### Model-free analysis

To characterize the amplitudes and time scales of intramolecular motions, the relaxation rate constants *R*_1_, *R*_2_, and the steady-state heteronuclear *^15^N{^1^H}* NOE were analyzed using the Lipari–Szabo model-free formalism [56]. Uncertainties in *R*_1_, *R*_2_, and *^15^N{^1^H}* NOE values were set to a minimum of 3% of the measured value. Default values for the nitrogen-hydrogen bond length (*r*_NH_) of 1.02 Å was used in the analysis. We further used fixed the ^15^N chemical shift anisotropy (Δσ) to a single default value for all residues (–160 ppm) [57] as only single field relaxation data were available.

Diffusion tensors were initially determined using quadratic_diffusion software [58] using the structural ensemble of 20 conformers and the *R*_1_, *R*_2_ data of the rigid core (47–100) as input. For both free and DNA-bound Sox2^31–127^, an axially symmetric diffusion tensor yielded a statistically significantly better fit than an isotropic diffusion model, while a fully anisotropic model offered no significant improvement. This is in reasonable agreement with the profile of *R*_1_*R*_2_ versus *R*_2_/*R*_1_ values [59] (Supplementary Fig. S11).

The estimated axially symmetric global diffusion tensor parameters were subsequently optimized in ModelFree4 [57], including only residues with *^15^N{^1^H}* NOE > 0.5 (free) and > 0.6 (DNA-bound). In the subsequent step, diffusion parameters were fixed, and the highly flexible N- and C-terminal regions were included using a fix algorithm for model refinement. Statistical analysis for model selection was achieved by in-house script following the procedure of Mandel *et al.* [57]. All final fitted parameters are reported in Supplementary Tables S2 (free) and S3 (bound).

### CPMG relaxation dispersion analysis

Residues with significant dispersion of their effective ^15^N *R*_2_ transverse relaxation rate constant (*R*_2, eff_) free state Sox2^31–127 ^were defined when Δ*R*_2_ [*R*_2,eff_ (highest (ν_CPMG_)) – *R*_2,eff_ (lowest (ν_CPMG_))] was >1.5 s^–1^and the fit of *R*_2,eff_ to a no-exchange model (constant *R*_2,eff_) had χ^2^_red_ > 4. Relaxation dispersion curves were acquired at 900 and 1200 MHz of in total 26 residues were fitted to a two-site exchange model (2st) using the ChemEx program (https://github.com/gbouvignies/ChemEx). As input parameters τc was taken from model-free analysis (9.25 ns), while population of states (*p*_B_), exchange rates (*k*_ex,AB_), and chemical shift difference (Δω_AB_) was left as defaults. The ^15^N chemical shifts derived from ^15^N-HSQC spectra together with the experimental *R*_1_ and *R*_2_ values were included as input. Initial single-residue fits (2st_rs) were performed, followed by grouping residues in two group based on similar *k*_ex,AB_ and *p*_B_ for a separate global fitting.

### Molecular dynamics simulations

*Starting structures*. For the free Sox2 we selected three different models from the NMR-based generated ensemble. The sequence of the models was GSNQKNSPDR VKRPMNAFMV WSRGQRRKMA QENPKMHNSE ISKRLGAEWK LLSETEKRPF IDEAKRLRAL HMKEHPDYKY RPRRKTKTLM KKDKYTL. The models of Sox2 bound to the FGF4 DNA element were built based on the 1GT0 structure [28]. The sequence of Sox2 in these models was identical to the that in the free-state model, with exception of N32 instead of S32 and addition of P128–G129 to the C-terminus. The N- and C-terminal tails missing from the 1GT0 structure were added with MODELLER [60] using the DOPE Loopmodel procedure in which the tails were defined as loops. 25 models and 25 loopmodels were generated and the best three ranked using the Normalized DOPE score were selected for the simulations. The sequence of FGF4 DNA element in these models was taken from the human FGF4 enhancer 5′-TTCCTTTTGA AAACTCTTTG TTCGAATGCA AATCATC-3′ (Sox2 recognition motif underlined). The DNA structure from the 1GT0 structure was extended using idealized B-DNA segments generated with the NAB program (part of the AMBER software [61]). The sequence was adapted using the swapna function in Chimera [62].

Each model was solvated in water using the four-point OPC water model [63] and neutralized with 16 Cl^−^ ions (free Sox2) or 58 Na^+^ ions (Sox2–DNA complex). In addition 150 mM KCl was added as buffer. The Li-Merz parameters optimized for the OPC water model were used for the ions. The ff19SB force field [64] was used for the protein and the parmbsc1 [65] for the DNA. The size of the solvated periodic box was chosen to allow sufficient space for the motions of the Sox2 tails. In the free Sox2, the minimal distance between the solute and the box edges was 16.7 Å, in the Sox2–FGF4 complex this was 12 Å. This resulted in systems with the size of ~110 000 atoms for the free Sox2 models and 160 000 atoms for the DNA-bound Sox2 models.

*Energy minimization, equilibration, and production simulations*. The systems were minimized in 25 000 conjugate gradient steps using the AMBER software [61] without any positional restraints to allow for the entire systems to relax. Then, the systems were equilibrated for 23 stages totaling 15.55 ns in NAMD [66]. In the first stage, the temperature was raised from 20 to 300 K for 150 ps in the NVT ensemble using Langevin dynamics (the Langevin damping coefficient was 5 ps^−1^). In the second stage, the density of the system was adjusted to 1 atm for 150 ps in the NPT ensemble using the Nose–Hoover Langevin Piston barostat (Langevin piston period was 100 fs and decay 50 fs). In these stages, the solute motion was restrained with positional restraints. During the next 18 stages (250 ps each), the protocol was kept and the positional restraints were gradually removed. For the Sox2–DNA complexes, additional restraints were added to keep the base pairs of the DNA formed to avoid any potential artifacts due to the procedure to adapt the DNA sequence. The stage 21 was 1.5 ns long and free of any restraints. In the stage 22 (2.25 ns long), the time step was increased from 1.0 to 1.5 fs whereas in the last stage a timestep of 2 fs was applied. The SHAKE algorithm was used to maintain bonds involving hydrogen atoms rigid. The Particle Mesh Ewald method was used for evaluating long-range electrostatic interactions. A cutoff of 10 Å was used for the short-range interactions. During the production runs, the Langevin damping coefficient was decreased to 0.1 ps^-1^, whereas the Langevin piston period and Langevin piston decay were set to 2000 and 1000 fs, respectively. The simulations were visualized in VMD [67] and analyzed in CPPTRAJ [68].

## Results

To study the structure and dynamics of Sox2–DBD, we designed a human Sox2 HMG construct with extended C- and N-terminal IDR (Sox2^31–127^) as shown in Fig. 1A. At the N-terminus, a positively charged region of 16 residues is included, leaving out the low-complexity region of residues 1–30. At the C-terminus, the construct includes a highly positively charged region after the HMG, up to L127, after which the Sox2 sequence become much less charged (Fig. 1A). This extended DBD construct can thus be expected to be able to capture the DNA binding mode of the full-length protein. The construct expressed well, resulting in pure and monomeric protein (Supplementary Fig. S1).

### DNA and salts increase the thermostability of Sox2^31–127^

As a first step, we determined the thermostability of Sox2^31–127^ across a range of conditions reported in the literature and a in buffer with physiological salt concentration (20 mM Tris, 135 mM KCl/15 mM NaCl) and pH (7.3) (referred to as physiological-like buffer hereafter). The melting temperature (*T*_m_) ranged from 302.7 to 314.0 K (Supplementary Table S1and Supplementary Fig. S3). As the *T*_m_ in the physiological-like buffer (311.8 K) was close to the maximum value in this screen, we chose to use this buffer throughout.

Increasing the salt concentration in the physiological-like buffer resulted in slightly increasing *T*_m_ values, with a *T*_m_ of 312.9 K at 250 mM KCl (Fig. 1B) whereas higher or lower pH values showed no systematic effect (Fig. 1C). A large increase of *T*_m_ to 340.6 K was observed upon FGF4 DNA binding, indicating that specific DNA binding significantly stabilizes Sox2^31–127^ (Fig. 1D). Binding to a shuffled DNA lacking the TTGT target site (hereafter “random DNA”) resulted in a lower melting temperature of 334.6 K(Supplementary Fig. S3). Fits of the unfolding curves of free and FGF4 DNA bound Sox2^31–127^ highlighted strong increase in cooperativity of unfolding in the DNA bound state, as indicated by steeper slope and increased enthalpy of unfolding (176 kJ/mol versus 472 kJ/mol) (Fig. 1E and Supplementary Fig. S3).

### Solution NMR unraveled the structure of Sox2 HMG

To understand the folding of the free Sox2^31–127^ in detail, we turned to solution NMR spectroscopy. A^15^N-TROSY NMR spectrum of the free Sox2^31–127^ showed the wide dispersion of chemical shifts characteristic of a well-folded protein (Supplementary Fig. S4). Indeed, the backbone chemical shifts (92% assignment completeness) are consistent with the three helix structure as in the structure of the DNA complex [28, 69] (Supplementary Fig. S4). Including side chains, in total 91.5% of all resonances could be assigned. Most unassigned resonances were from side chain atoms of residues in the C-terminal part. In addition, backbone resonances corresponding to residues G31-N33, N68-S69, and R114 could not be found, indicative of significant line broadening. Specifically, G31-N33 and R114 are located in the N- and C-terminal IDRs, while N68 and S69 are part of the N-terminal cap of helix α2, which might also be solvent-exposed.

Encouraged by the data quality, we determined the solution structure of Sox2^31–127^ in its free state. To complement distance restraints from NOESY data, backbone amide RDCs were determined in a liquid crystal phase generated with 5% C_10_E_5_/*n*-hexanol. The ^1^H–^15^N-HSQC spectra under aligned and unaligned conditions show minimal chemical shift changes, indicating the protein structure remains stable and unaffected by C_10_E_5_/*n*-hexanol (Supplementary Fig. S5). In a total 43 backbone amide RDCs, spanning between –12.9 Hz and 25.3 Hz (Supplementary Fig. S5), were used in the structure calculation together with 1577 NOESY-based distance restraints, of which 245 long-range (see Table 1).

**Figure 2.**
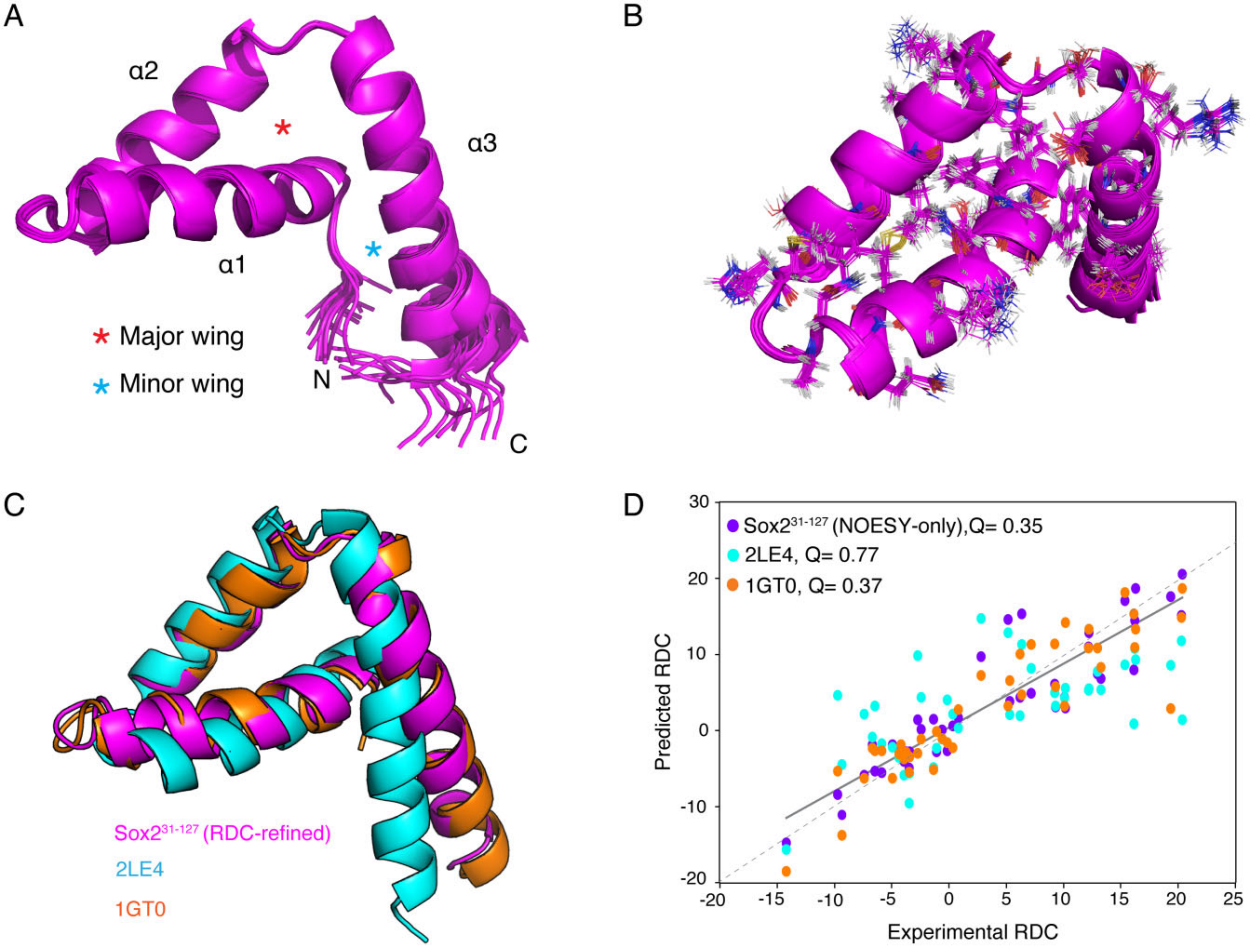
Solution structure of Sox2^31–127^. (**A**) Cartoon-representation of Sox2^31–127^ ensemble (residues 42–106), exhibiting the characteristic HMG domain L-shaped three-helical bundle. Helices and HMG major and minor wings are indicated. (**B**) Side-chain conformations within the HMG core (residues 47–103), displaying only those with heavy atom RMSD < 1 Å. (**C**) Structural alignment of the RDC-refined Sox2^31–127^ structure (HMG residues 47–104) with an unpublished free state structure (PDB: 2LE4) and the DNA-bound structure (PDB: 1GT0). (**D**) Correlation plot comparing experimental and structure-predicted backbone amide RDCs, with Q-factors in the legend.

The final RDC-refined ensemble of Sox2^31–127^, comprises the 20 lowest-energy conformers, has a well-defined three-helix bundle with a characteristic L-shape fold, as observed in other HMG domains (Fig. 2A and B, see Table 1 for structural statistics) [26]. The HMG domain spans from residue 47 to 103, with helix α1 spanning between residue 47 and 62, α2 from residue 68 to 81 and α3 from residue 84 to 103. The HMG major wing is formed by helix α1 and α2 and minor wing by α3 and residues 41–46 in the N-terminus [25]. Both helix α1–α2 and α2–α3 are connected by two HH (alpha-alpha) loops according to the ArchDB [53] classification, ranging from 63 to 67 and 82 to 83, respectively. Residues 31–46 in the N- and 104–127 in the C-terminal tails are unstructured.

The primary hydrophobic core is localized within the major wing and helix α3 involving residues W51, W79, F48, and F90. This core is stabilized by an extensive π–π interaction network (Supplementary Fig. S6). These interactions are known to play a critical role in maintaining the structural integrity and stability of the overall protein fold [70]. Additionally, a smaller hydrophobic cluster between A47, V50, A94, and L97 and between I71 and L75 support the formation of the L-shaped helical bundle (Supplementary Fig. S6).

Comparison to the unpublished solution structure of free Sox2 HMG (PDB: 2LE4) shows significant structural differences (heavy atom backbone RMSD 3.7 Å for residues 47–103). Most notable difference is the orientation of helix α3 (Fig. 2C), which is at an angle with helix α1 of 107° in our structure, compared to is 77° in 2LE4. Comparison of the 2LE4 structure against the experimental RDCs resulting in a very poor Q-factor (0.76). In comparison, the RDC-refined structure of Sox2^31–127^ has a *Q* of 0.08, which increases to 0.35 for a structure based only on NOESY data, i.e. without RDC refinement. Thus, the 2LE4 structure does not fully capture the free state structure of Sox2 HMG.

Closer analysis showed that the side-chain orientations in the primary hydrophobic core of Sox2^31–127^ are notably different in 2LE4 (Supplementary Fig. S7), suggesting that the different helical orientations may be due to an assignment issue of the aromatic residues in 2LE4. A fully automated structure calculation using the NMRtist platform [55] resulted in fewer assignments of the aromatic residues compared to our manual analysis, resulting in different packing of aromatic core residues (Supplementary Fig. S8 and see Materials and methods for details). Importantly, the automatic calculation resulted in the same helical bundle structure as in the manual run. We conclude that the NOESY-based and RDC-refined solution structure of Sox2^31–127^ determined here accurately captures the conformation of Sox2-DBD in its free state.

### Free state Sox2 HMG fold mirrors the DNA bound state

Strikingly, the DNA-bound structure of Sox2 HMG domain (PDB 1GT0 [28]) resembles the free state Sox2^31–137^ closely, with heavy atom backbone RMSD of 0.8 Å (Fig. 2C). The helices in the free state assume the same orientation as in the DNA-bound state. The crystal structure of the DNA-bound state indeed exhibits a similar Q-factor as the Sox2^31–127^ structure calculated from NOESY data only, demonstrating agreement with the experimental backbone amide RDCs for both structures (Fig. 2D). Notably, the side chain orientations in the primary hydrophobic core superimpose closely in free and DNA-bound forms. (Supplementary Fig. S7).

To verify the similarity of Sox2^31–127^ conformation in free and DNA-bound forms in solution, we compared circular dichroism spectra of free and FGF4 DNA-bound Sox2, confirming a largely unchanged overall secondary structure (Supplementary Fig. S9). To obtain a more detailed analysis, we assigned the backbone chemical shifts of Sox2^31–127^ bound to DNA (95% completeness). The backbone chemical shifts of DNA bound Sox2^31–127^ point to an extension of the α3 helix to E104 and stabilization of the final helical turn upon DNA binding, in line with the crystal structure (Supplementary Fig. S10).

When examining the conformations of the N- and C-terminal tails of the HMG domain, it is notable that, due to several long-range NOEs to the helix α3, the N-terminal tail is relatively well-defined in the free-state ensemble in a conformation that is similar to the DNA bound state (Supplementary Fig. S10). Residues 106–114 in the C-terminal tail are completely unstructured in the free state but are well-ordered in the DNA-bound state, thanks to interactions with DNA and/or HMG residues. Interestingly, residue H101 that upon DNA binding forms a hydrophobic cluster with V41, P44, Y108, and H105, is positively charged in the NMR structure of the DNA-complex (PDB: 1O4X [69]). We determined the His side chain p*K*a values in both free and FGF4 DNA-bound states and found that, at physiological pH, H101 is neutral in the complex (p*K*a 4.1), consistent with its buried position (Supplementary Fig. S11).

Altogether these data indicate that the HMG core fold is encoded in the Sox2 primary sequence and that structural changes upon DNA binding are limited to the folding of the N- and C-terminal IDR regions.

### Part of the C-terminal IDR of Sox2 rigidifies upon specific DNA binding

**Figure 3.**
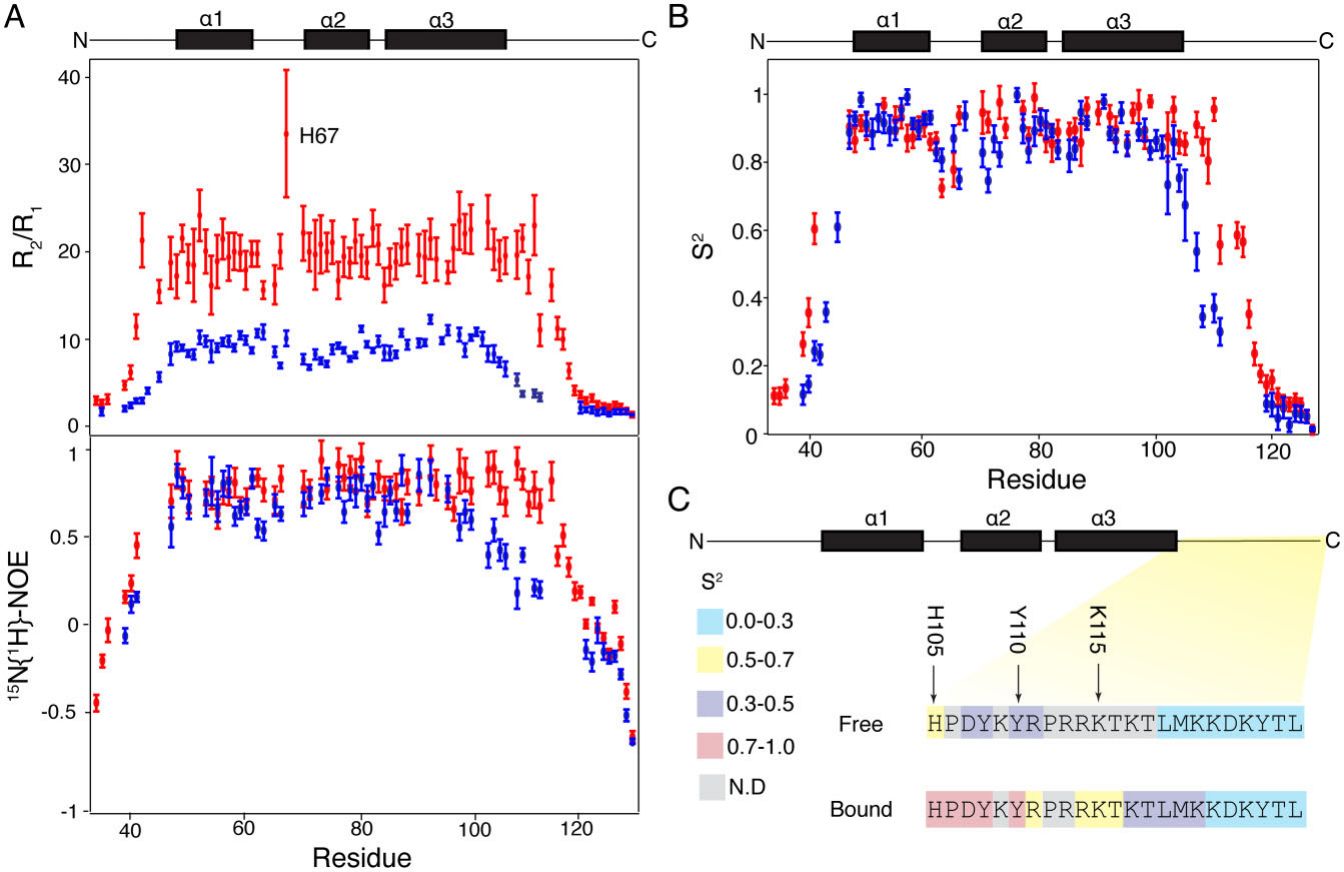
Fast (ps–ns) backbone dynamics of Sox2^31–127^ in free and FGF4–DNA bound state. (**A** and **B**) Backbone ^15^N *R*_1_/*R*_2_ relaxation rate constant ratio and *^15^N{^1^H}* NOE values (A) and derived model-free *S*^2^ values (B) in free (blue) and DNA bound state (red), with secondary structure indicated above the panel. (**C**) Analysis of *S*^2^ values and changes upon DNA binding within the C-terminal IDR region. Color coding based indicated; ND is “no data.” The arrows indicate the start of the early, middle, and late C-terminal regions.

To further assess the impact of specific DNA binding, we inspected the fast time-scale (ps–ns) motions of the Sox2^31–127^ backbone in free and FGF4 DNA bound states, extracted from the ^15^N backbone amide *R*_1_ and *R*_2_ relaxation rate constants and *^15^N{^1^H}* NOEs (Fig. 3A). The DNA binding slows the overall molecular tumbling of Sox2, reflected in the increased *R*_2_/*R*_1_ ratios. Further analysis of the relaxation data indicated axially anisotropic diffusion for both free and DNA-bound Sox2^31–127^ (Supplementary Fig. S12), with an increase of the global tumbling time (τ_m_) from 9.3 to 14.2 ns upon specific DNA binding (Supplementary Tables S2 and S3).

Interestingly, H67 has elevated *R*_2_/*R*_1_ compared to the overall trend in the DNA bound state, indicative of exchange broadening. Residue H67 is positioned at the end of the first loop (N63–H67) and is close to the DNA in both the crystal and NMR structure of the DNA complex (PDB: 1GT0 and 1O4X [28, 69]). As H67 forms a hydrogen bond to the DNA backbone in 1O4X but not in 1GT0, we determined its protonation state in both free and DNA bound states. The p*K*a of H67 increases from 5.76 to 6.25 upon DNA binding, indicating a modest stabilization of the protonated state due proximity to the DNA (Supplementary Fig. S11). Around neutral pH, H67 will be partially protonated and thus there may be chemical exchange between a protonated state, hydrogen-bonded to the DNA, and the neutral state that is not closely interacting with the DNA.

To quantify the local fast time scale motions, the relaxation data were analyzed using the model free formalism [56] to extract for every residue the order parameter (*S*²), and, where applicable, the exchange constant (*R*_ex_), and the internal residue tumbling time (τ_e_) (summarized in Supplementary Tables S2 and S3). The *S*² measures the amplitude of motion of the backbone amide bonds, ranging from 0 to 1, where 0 indicates complete isotropic motion and 1 signifies no internal motions.

Upon DNA binding, the average *S*² for the core HMG domain (47–100) remained comparable to the free state, increasing slightly from 0.81 ± 0.03 to 0.83 ± 0.04 indicating that the HMG domain does not rigidify. In contrast, consistent with the chemical shift based secondary structure analysis (Supplementary Fig. S10), we observed an increase of *S*² at the end of helix α3 when Sox2^31–127^ is bound to DNA compared to the free state, indicating enhanced rigidity (Fig. 3B and C). Additionally, regions of the N- (residues 40–45) and C-terminal (residues 105–115) IDR that are part of the DNA binding interface exhibited increase in *S*^2^ rigidity upon DNA binding, consistent with their role in the binding interface.

For the sake of clarity, we divide the C-terminal IDR in an early, middle, and late part, depending on the observed level of rigidification. The early region, from residue H105 to Y110, exhibited a strong increase in *S*², from 0.48 ± 0.06 to 0.89 ± 0.04 (Fig. 3C). The increased rigidity of this segment is in line with the formation of specific interactions between DNA and HMG residues upon binding [28, 69]. For the middle region, R111 to K115, a smaller increase in *S*² is observed, from 0.30 ± 0.04 to 0.57 ± 0.05. This is consistent with their binding in the DNA minor groove, anchored by R114. Of note, while R114 could not be assigned in the free state, its backbone resonances are clearly visible in the DNA-bound state. In contrast, the late C-terminal IDR (K115 to L127) displayed persistently low *S*² values, going from 0.06 ± 0.02 to 0.12 ± 0.03, indicating that this segment retains substantial flexibility even upon DNA binding (Fig. 3C).

These findings demonstrate that while the HMG domain does not rigidify upon DNA binding, the early C-terminal IDR undergoes a disorder-to-order transition, acquiring a rigid structure upon specific DNA binding. This transition highlights the critical role of the highly positively charged early and middle C-terminal IDR in facilitating specific DNA interactions, whereas the late C-terminal IDR does not appear to play a significant role.

### Sox2 HMG transitions between a major folded state and partially unfolded states

**Figure 4.**
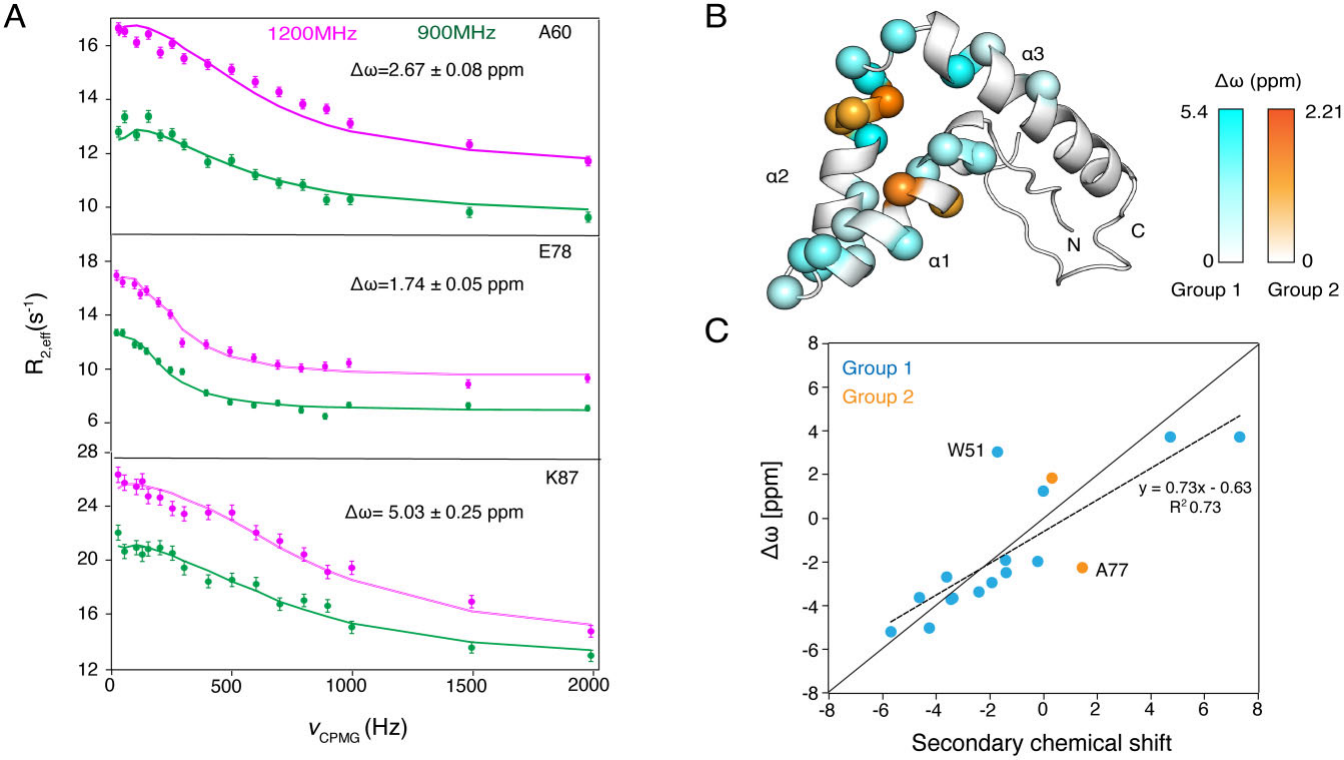
Slow (μs–ms) backbone dynamics in the Sox2^31–127^ free state. (**A**) Relaxation dispersion plots recorded at 900 and 1200 MHz for residues from each of the three α helices. Best fit curves and chemical shift difference between major and minor states ( Δω) indicated. (**B**) Magnitude of Δω color-coded onto Sox2^31–127^ structure (only 39–118 displayed). (**C**) Correlation plot between difference to random coil chemical shift from the major state (secondary chemical shift) and the fitted Δω (with signs derived from HSQC/HMQC comparison). Best-fit correlation indicated. Two outliers to the fit are labeled.

We next compared the slow time scale (μs–ms) dynamics of the free and DNA bound Sox2^31–127^ using CPMG relaxation dispersion experiments. Data were recorded at high magnetic fields (900 and 1200 MHz) to make use of the magnet field-dependent impact of slow conformational dynamics on the NMR signal. In the free state, we observed significant dispersion of transverse ^15^N relaxation rates (*R*_2,eff_) for 26 residues mainly localized in the major wing of the HMG (Fig. 4A and B;Supplementary Fig. S13 and Table S4), while both N- and C-terminal IDRs did not show any significant dispersion.

To further characterize the conformational exchange, the dispersion data were fitted using a two-state model in which the major, ground state (*A*) interconverts stochastically with a minor, higher energy state (*B*) with an intrinsic rate constant *k*_ex_, defined as the sum of forward and backward reaction rates (*k_AB_* + *k_BA_*) and chemical shift difference Δω between state *A* and *B*. Residues were clustered into two groups (groups 1 and 2), based on their fitted *k*_ex_ and the fractional population of the minor state (*p_B_*).

Residues in group 1 exhibited a wide range of Δω ranging from 1.78 to 5.42 ppm, and exchanged on a sub-millisecond time scale (*k*_ex_ 3.5 ± 0.1 ·103 s⁻¹) to a sparsely populated minor state (*p_B_* 0.69 ± 0.03%). Residues in group 2 had Δω values ranging from 1.24 to 2.28 ppm, and exchange on millisecond time scale (*k*_ex_ 0.86 ± 0.05 ·103 s⁻¹) to a minor state populated to 1.20 ± 0.03% (Fig. 4B).

To further elucidate the nature of these minor states, we measured the sign of the chemical shift difference Δω and examined the correlation to the random coil chemical shifts (Fig. 4C and Supplementary Fig. S14). A clear, but not perfect correlation was found, indicating that the minor state resembles a partially unfolded state. This is in line with the limited thermostability of Sox2^31–127^. Based on the fitted thermostability curves (Fig. 1ESupplementary Fig. S3), the fraction unfolded species at 293 K is expected to be ∼1%, which is in reasonable agreement with the population of the minor state at this temperature.

Strikingly, upon stoichiometric addition of DNA, we observed a complete quenching of the conformational line broadening, indicating that the presence of DNA shifts the equilibrium toward the major folded state (Supplementary Fig. S15).

### High electrostatic environment stabilizes the Sox2 HMG fold

**Figure 5.**
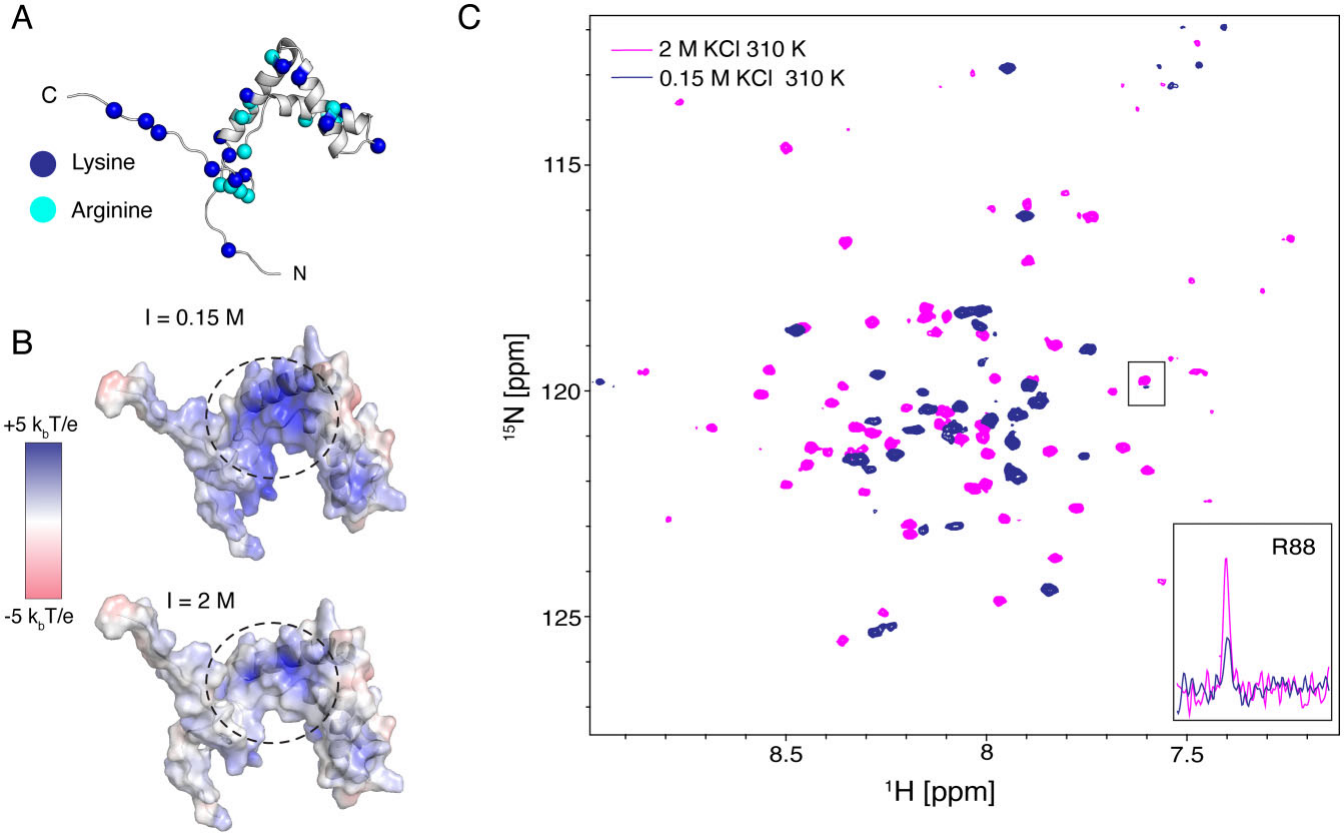
Electrostatic frustration and stabilization of Sox2^31–127^. (**A**) Structure of free-state Sox2^31–127^ with Cα position of lysines and arginines highlighted spheres. (**B**) Electrostatic potential surface of Sox2^31–127^ calculated at 310 K and 0.15 and 2 M ionic strength using the Adaptive Poisson–Boltzmann Solver (APBS) [71]. The dashed circle highlights a highly positively potential region at 0.15 M. (**C**) Overlay of ^15^N-TROSY spectra at 0.15 and 2 M ionic strength at 310 K. A 1D trace (bottom right) taken through the resonance of residue R88 is shown in the inset.

To get further insight into the stabilizing impact of DNA binding, we investigated the thermal stability and structure of Sox2^31–127^ as function of ionic strength. The net charge of Sox2^31–127^ is ~ +16 at pH 7.3, with pronounced clusters of positive charges localized in the N-terminal region, helix α3, and parts of the C-terminal IDR (Fig. 5A and B). These clusters can lead to unfavorable intramolecular electrostatic interactions that destabilize the HMG, in line with the low thermal stability (Fig. 1E).

A salt titration from 0.15 to 2 M KCl followed by NMR revealed significant CSPs for charged residues in helix α1 and the C-terminal end of helix α3, suggestive of small, local structural changes (Supplementary Fig. S16). NanoDSF measurements further confirmed enhanced thermal stability under high ionic strength. At 2 M KCl, the *T*_m_ increased by 8 K to 319.4 K, with negligible changes in enthalpy to Δ*H* of 180.1 kJ/mol, indicating retention of the global fold (see Supplementary Fig. S3).

At physiological temperature (310 K), NMR spectra of Sox2^31–127^ show striking differences between 0.15 and 2 M KCl (Fig. 5C). At physiological salt concentration (0.15 M) Sox2^31–127^ displayed overall much reduced peak intensities, mostly for resonances away from the ^1^H_N_ random coil chemical shift (8.2 ppm), see for instance R88 in the inset of Fig. 5C. The combination of high intensity resonances around 8.2 ppm and otherwise low-intensity resonances is typical of partial unfolding (expected fraction unfolded species is 41%) (Fig. 5C and Supplementary Fig. S17). Conversely, at 2 M KCl, the resonances were more dispersed with more homogenous distribution of peak intensities, reflecting a predominantly folded state (Fig. 5C and Supplementary Fig. S17). Based on the melting temperature at 2 M KCl, ~90% of the protein is expected to be folded at 310 K. Interestingly, the improved spectral appearance in terms of peak intensities and chemical shift dispersion at 310 K in presence of 2 M KCl are also observed in the presence of DNA (Supplementary Fig. S17). Together, the data suggest that a high local concentration of charges, either from salt or from DNA, is crucial in screening the intramolecular repulsion in the Sox2 HMG domain and stabilizing it in a folded conformation.

### Molecular dynamics simulations support stable folding of HMG domain and DNA binding impact

**Figure 6.**
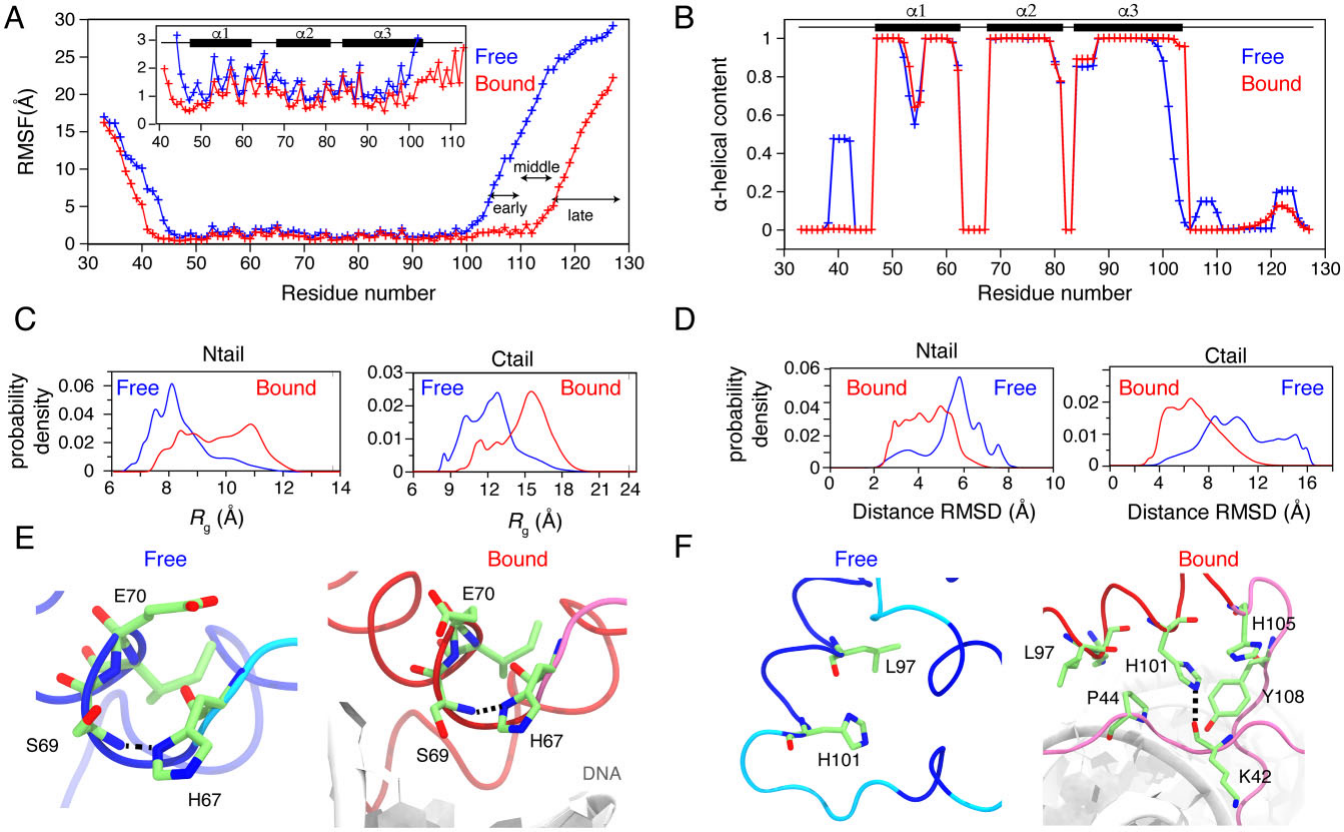
Molecular dynamics simulation of Sox2^31–127^ in free and DNA-bound state. (**A**) Average heavy atom RMSF per residue for free (blue) and DNA-bound Sox2–DBD (red) . The positions of the early, middle, and late C-terminal IDR are indicated. The inset shows a zoomed-in plot for the HMG region with secondary structure in the NMR ensemble indicated. (**B**) Fraction a-helical conformation per residue over the course of the simulation. Secondary structure in NMR ensemble is indicated. (**C** and **D**) Histograms showing the distribution of computed *R*_g_ (C) and distance RMSD (D) for the N-terminal (Ntail) and C-terminal (Ctail) IDRs in free and DNA-bound state over the course of the simulation. (**E** and **F**) Representative conformation of H67 (E) and H101 (F) in free and DNA-bound states, with side chain hydrogen-bonding indicated as black dashed lines.

To further test the stability of the Sox2 DBD, we next performed three independent molecular dynamics simulations of the free-state Sox2^31–127^ structure and of Sox2 bound to the FGF4 enhancer element. Each simulation was 5 µs long, thus reaching a total of 15 µs ensemble sampling for the free and another 15 µs for the DNA-bound Sox2. The root-mean-square-fluctuations (RMSF) averaged over all heavy atoms per residue showed that the HMG fold (residues 47–103) remains stable over the course of the simulations (Fig. 6A). The HMG helices were well-defined during the simulation for both free and DNA-bound Sox2^31–127^, with boundaries largely matching those observed in the free-state NMR structure determined here and the DNA-bound crystal structure (Fig. 6B). Importantly, the low RMSF and stable secondary structure for the free state HMG region support the experimentally determined structure and the rigidity of the HMG in the fast dynamics analysis (Fig. 3). Notably, the simulations highlight significant kinking of helix α1 at G54 (Fig. 6B). In addition, the stabilization of the last helical turn in a3 upon DNA binding, which was also evident experimentally (Supplementary Fig. S9 and Fig. 3), is clear in both from reduction in the RMSF (Fig. 6A) and increase in α-helical content (Fig. 6B) in the simulations.

The N- and C-terminal tails show high positional variance in both free and DNA-bound Sox2 (Fig. 6A and D), consistent with their high dynamics seen experimentally (Fig. 3). The fluctuations of both tails were higher in the free Sox2, as in the DNA-bound Sox2 their motion was somewhat restricted by interactions with DNA of some of their residues. Consistent with the experimental fast dynamics data (Fig. 3), the early and middle C-terminal residues show a strong reduction in RMSF to values comparable of HMG residues (Fig. 6A). Although the tails sampled mostly extended conformations in all simulations, they were overall more compact than free Sox2, as seen from lower radius-of-gyration (*R*_g_, Fig. 6C), indicating their interaction with the linear DNA in the DNA-bound state results in a more extended conformation.

Inspection of H67 and H101 side chain motion revealed that the sampling of the H67 side chain is restricted to some extent by a hydrogen bond with the backbone amide of S69 ( Supplementary Table S5 and Fig. 6E). The occupancy of this hydrogen bond is less in the DNA-bound Sox2 where H67 transits between conformations pointing toward or away from the DNA without establishing stable interactions with the DNA. These findings are in line with the modest effect on the p*K*a of H67 seen experimentally. The motion of the H101 side chain is restricted in the DNA bound Sox2 by a dynamic hydrogen bond with the backbone of K42 as well as hydrophobic and π–π interactions with P44, H105, and Y108 (Supplementary Table S5 and Fig. 6F). The burial of H101 side chain upon DNA binding is consistent with extreme shift in p*K*a value, as noted earlier.

### Sox2^31–127^ has distinct specific and nonspecific DNA-binding modes

To get more insight into the DNA-binding mechanism, we compared Sox2^31–127^ binding to both FGF4 DNA and a random DNA sequence lacking the cognate binding site using NMR titration experiments. Addition of random DNA resulted in rapid line broadening of most resonances, even at excess DNA (up to 2.3 molar equivalent), precluding assignment of the fully bound state. This reflects a lack of stable complex formation, suggesting that Sox2 interacts through multiple, transient binding poses rather than forming a distinct complex when the TTGT motif is absent.

**Figure 7.**
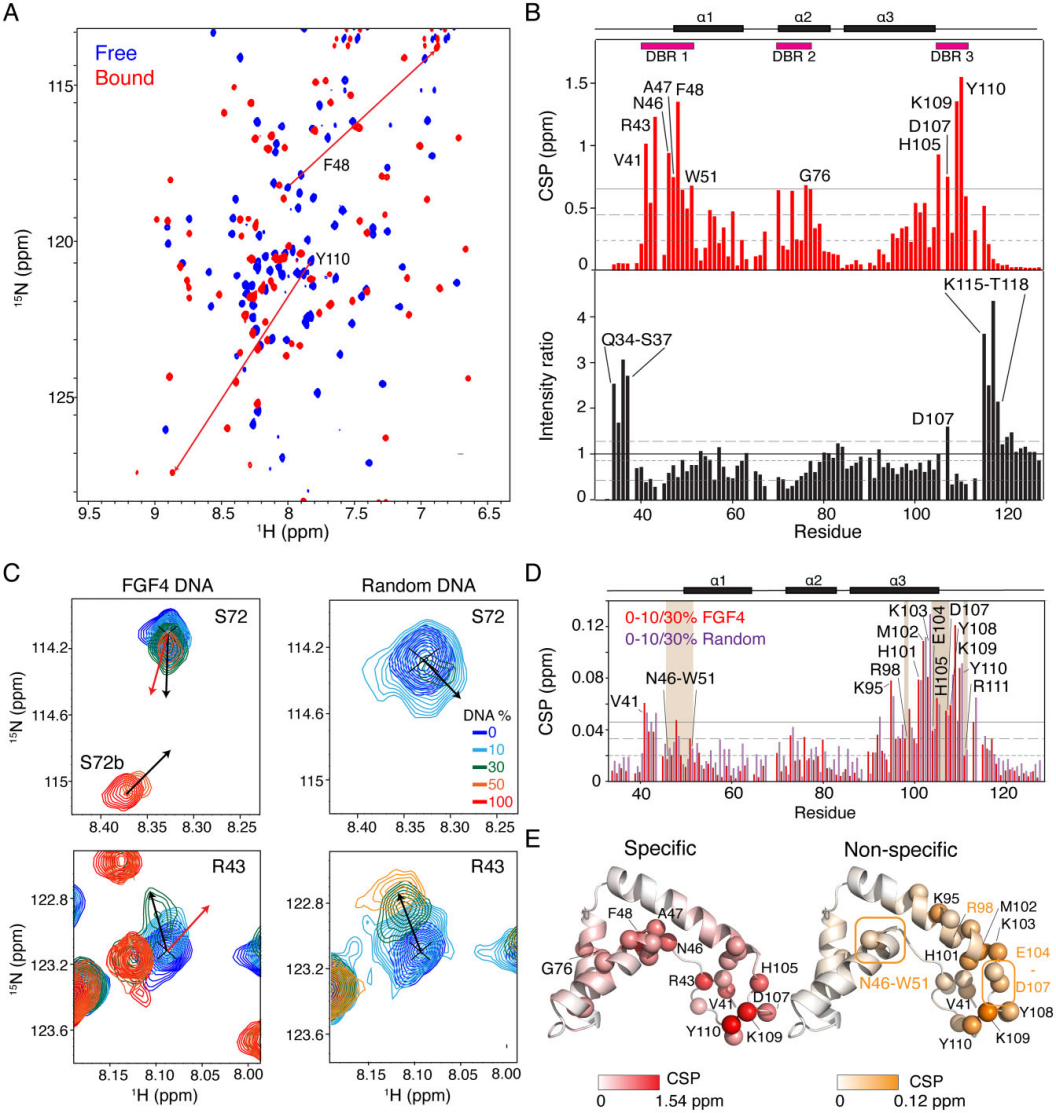
NMR titration of Sox2^31–127^ with FGF4 DNA (**A**) Overlay of ^15^N-TROSY spectra of Sox2^31–127^, free (blue) and in presence of 100% FGF4 DNA (red), with chemical shifts changes indicated for two residues. (**B**) CSP between free and FGF4 DNA-bound states (top) alongside intensity ratios. Thresholds at 10% trimmed mean (short dashes) plus one (long dashes) or two (solid line, in the case of CSP plot) standard deviation (SD), and three DNA-binding regions (DBRs) indicated. Secondary structure indicated on the top of the panel. (**C**) Chemical shift trajectories of S72 and R43 upon binding specific (FGF4) and random DNA. Black arrows indicate the direction of CSP trajectories in either early or late titration phase, red arrows indicate the direction CSP from free to fully bound state. (**D**) CSP upon DNA binding in the initial phase of the titration (up to 30% molar equivalents DNA added) for both FGF4 and random DNA. Brown-shaded boxes indicate CSPs calculated on the basis of the 10% point because of signal loss at higher molar ratios. (**E**) CSPs for specific binding (upon addition of 1 molar equivalent FGF4 DNA) and nonspecific binding (upon addition of 30% molar equivalent FGF4 DNA). Spheres indicates residues with CSP higher than 10% trimmed mean + 1 SD; residues with CSP higher 10% trimmed mean + 2 SD are labeled. Residues with signal loss at 30% point are indicated in box and with orange font.

Addition of FGF4 DNA resulted in a clear and drastically different peak pattern, indicative of stable and specific binding (Fig. 7A). CSP mapping between free and fully bound states revealed that affected residues are localized within the folded HMG core and the N- and C-terminal IDR regions. Among these, R43, N46, F48, M49, W51, H67, K109, and Y110 were previously identified as critical for specific DNA binding (Fig. 7B) [72]. Additionally, residues V41, G76, E70, and K115 have significant CSPs, indicating these contribute at least indirectly to the specific DNA-binding mode (Fig. 7B and Supplementary Fig. S18).

Relative peak intensities of N-terminal and, especially, the late C-terminal IDRs increase significantly upon DNA binding (Fig. 7B). Although the *S*² values for both N- and late C- terminal IDRs indicate that these region remains dynamic upon binding, their solvent exposure may be altered due to the proximity of with the DNA, reducing solvent exchange [73].

### Sox2 binds specific DNA initially in a nonspecific binding mode

Closer inspection of the titration data showed that addition of both specific (FGF4) and nonspecific (random) DNA induced similar CSPs during the early titration phase (0%–30% molar equivalents DNA added), see Fig. 7C. At 10% DNA added, the majority of residues followed very similar trajectories for both specific and nonspecific DNA (Fig. 7C and Supplementary Fig. S19). At 30% DNA, the signals for residues N46, A47, F48, M49, V50, W51, K95, R98, M102, E104, H105, Y110, and R111 were broadened beyond detection for both FGF4 and random DNA, while the remaining residues maintained similar chemical shift trajectories. These findings indicate that at high Sox2-to-DNA ratios nonspecific interactions dominate, even when a specific target sequence is present.

At 50% FGF4 DNA added, two distinct signals can be observed for several residues, reflecting the co-existence of both nonspecific and specific binding: one following the initial CSP trajectory, and another in intermediate-to-slow exchange, displaying a significantly different CSP trajectory (see Supplementary Fig. S72 in Fig. 7C). Comparison of CSP directions across the FGF4 DNA titration revealed significant differences between the early and the fully saturated phases for most of the residues in the DNA-binding interface (Fig. 7C and Supplementary Fig. S19). This suggests a two-step binding mode during the titration with DNA, involving a transition from initial nonspecific to a final specific interaction, both mediated by largely the same DNA binding surface on Sox2^31–127^.

Analysis of the CSPs in the initial titration phase with either FGF4 or random DNA (0%–30% molar equivalent DNA added) highlights that the interaction mode under these conditions is very similar for both DNA types (Fig. 7D). The similarity of the CSP patterns further underscores that these data reflect a nonspecific binding mode. Compared to the specific interaction mode (Fig. 7B), it is notable that while the CSPs cluster in the same three DNA binding regions, the overall pattern is different (see also Fig. 7E). The interaction surface for the nonspecific mode observed at high Sox2:DNA ratios does not involve as much the major wing and is dominated by residues at the base of the minor wing and the early C-terminal tail. Accordingly, close inspection of the titration data shows significant different CSP directions for minor wing residues in the FGF4 titration compared to random DNA (Supplementary Fig. S19).

An NMR titration experiment with FGF4 DNA conducted at 500 mM ionic strength showed a much reduced contribution from this nonspecific-binding mode (Supplementary Fig. S20). Despite the presence of apolar residues in the interface, the nonspecific binding is thus mostly driven by electrostatic interactions. Notably, the specific binding still occurred in the slow exchange regime, indicating the specific interactions are largely insensitive to the increased ionic strength.

## Discussion

The function of pTF Sox2 depends on its ability to recognize and bind its target DNA sites within an overwhelming excess of nonspecific sites. Sox2 binds sequences containing a TTGT core motif within diverse flanking sequences; these sequences can be either in fully accessible, “naked,” DNA or embedded at different positions within a nucleosome. Several authors have argued that intrinsic disorder in the HMGs such as Sox2–DBD is essential to permit binding within such diverse sequence and structural contexts [6, 23, 30–32, 72]. Here, we critically examined this hypothesis through a study of the stability, structure, dynamics and DNA interactions of the Sox2 HMG domain (Sox2^31–127^).

The so-called “floppy” Sox model suggests that Sox HMG domains lack a well-defined tertiary structure in their free state, and undergo a disorder-to-order transition upon DNA binding to adopt a stable, folded conformation [30, 31]. As the DNA is bent in the Sox–DNA complex, binding is thought to occur through a mutual induced fit mechanism. This model was initially proposed by Weiss based on random coil chemical shifts for V41 and lack of long-range NOE between V41 and Y108 in Sry that are part of the minor wing in the DNA bound state. As a result, helix α3 was suggested to be partially released from the major wing helices α1 and α2 in the free state [30].

This model was seemingly supported by the unpublished free-state Sox2–DBD structure (PDB: 2LE4) that features significant rearrangement of helix α3 compared to the DNA-bound state. However, backed by careful analysis of NOESY and RDC data and supported by MD simulations, we find that the Sox2 HMG has the same helical orientations as in the DNA-bound state. Furthermore, based on a complete set of ^15^N backbone NMR relaxation experiments, we find that the HMG has very similar rigidity in the free and bound states, while the N- and C-terminal tails become much more rigid upon binding. Previous work on Sox2 HMG reached the same conclusion on the tails based on analysis of ^15^N backbone transverse relaxation rates but could not extract the backbone dynamics of the HMG core [23].

We thus conclude that the helical bundle structure of the HMG domain is intrinsically encoded in the sequence and that the structural changes in the Sox2–DBD upon DNA binding are limited to (i) stabilization of the helix α3 end (H101–E104); (ii) rigidification of the N-terminal tail (R40–M45); and (iii) folding and rigidification of the early and middle regions in the C-terminal IDR. The latter regions are considered as a key part of the DNA-binding regions contributing to both binding affinity and specificity [74]. Stabilization of these regions into their defined DNA-bound structure is however independent of the HMG helical bundle structure as we show here.

The structural changes upon DNA binding for Sox2 are thus much more in line with that of family members Sox5 and Sox17 for which no changes in HMG helical structure were observed [33, 34]. AlphaFold-3 [75] predictions of all 20 human Sox HMG domain structures resulted highly comparable helical content and orientations, with minimal changes compared to the available DNA-bound structures (Supplementary Fig. S21), although the predicted free state structures may be biased towards the DNA-bound state by over-representation in the PDB [76, 77].

Still, we found the Sox2–DBD to have limited thermostability (*T*_m_ 311.8 K at physiological ionic strength and pH). At 293 K, the folded state continuously and rapidly interconverts with a small (∼1%) fraction of a minor conformational state, affecting mostly the major wing. The presence of such minor state with alternate conformation was anticipated in previous work on Sox2 based on partial NMR relaxation data [23]. Here, using CPMG relaxation dispersion data recorded at 900 and 1200 MHz, we could extract the characteristics of this conformational exchange process. While the minor state chemical shifts correlate with the random coil chemical shifts (Fig. 4C), there are significant outliers to this trend and several residues with sizeable secondary shifts did not show dispersion of their ^15^N *R*_2,eff_ values. Together, this suggest the minor state represents a partially unfolded molecule rather than a globally unfolded state. In addition, the need to fit the CPMG data in two groups indicates that there are multiple minor states present, which could include the globally unfolded state and one or more partially unfolded states. To what extent the DBD stability and conformational dynamics is further influenced by the IDR regions, e.g. through dynamic interaction between IDR and DBD [78] and cellular conditions remains to be established.

A similar low thermostability has been found for other HMG-box proteins in extensive biophysical analyses by the Privalov lab [79–81]. These studies also showed that DNA binding result in marked increase in stability, similar to what we observed here for Sox2^31–127^. The *T*_m_ increased by ∼29 K to 340.8 K upon binding FGF4 DNA (Fig. 1 and Supplementary Fig. S3) and interconversion of the folded ground state to an unfolded state could no longer be detected in the CPMG relaxation dispersion experiments.In the complex, one face of the Sox2–DBD spanning both major and minor wings is tightly packed on the DNA, forming numerous intermolecular interactions, including base-pair specific hydrogen bonds [28]. These likely contribute to the much larger enthalpy of Sox2–DBD unfolding in the bound state compared to the free state (Δ*H* 472 versus 176 kJ/mol, see Supplementary Fig. S3).

Binding to a random DNA lacking the TTGT motif also resulted in a pronounced Sox2–DBD stabilization (*T*_m_ increased by ∼23 K to 335.2 K, Supplementary Fig. S3). Previous work on other sequence-specific HMG boxes, including Sox family member Sox5, showed similar stabilization when using noncognate DNA [81]. It is notable that fit of the melting data is rather poor (Supplementary Fig. S3). Together with the lack of a well-defined NMR fingerprint for this complex, this indicates a dynamic Sox2–DNA interaction consisting of multiple binding poses with each sufficient favorable interactions to stabilize the protein.

We found also found a clear, yet modest, increase in thermal stability upon increasing ionic strength (Fig. 1C), leading up to a ∼8 K increase at 2 M KCl (*T*_m_ 319.4 K, Supplementary Fig. S3). High net charge and clustering of like charges can reduce thermostability due to electrostatic repulsion in the folded state [82]. Increasing ionic strength can help to screen these interactions and thus to stabilize the protein, as has been shown for the CytR DBD [83]. Model calculations using the Tanford–Kirkwood-Solvent-Accessbility (TKSA) method [84] indicate that while the protein has overall favorable electrostatics, many charged residues are predicted to have unfavorable electrostatic energy (Supplementary Fig. S22). Interestingly, these cluster in the major wing in helix α1, overlapping partly with the residues with significant μs–ms dynamics, the C-terminal end of α3 and the C-terminal tail. Computational alanine-scanning supports relatively low stability at these sites (Supplementary Fig. S22).

Next to DNA binding and high ionic strength, an additional factor in the stabilization of Sox2–DBD in presence of DNA may be the electric field of the DNA itself. A recent study by Munshi *et al.* proposed that unstable TFs may fold upon their approach to the DNA [83]. As a poly-anion, DNA produces an electric field which extends a few nanometers from its surface, possibly mediated by structured water molecules [85]. Based on salt-induced electrostatic folding on CytR DBD (Cytidine repressor), Munshi *et al.* proposed that charged phosphates of the DNA backbone could provide sufficiently high electrostatic environment to quench the frustration in the highly positively charged DBDs [83]. Their results indicated an increase of ∼15 K in *T*_m_ upon approaching the DNA. Even if for Sox2–DBD the stabilizing effect is more modest, a sizeable increase in the fraction folded species is likely. For example, a 2 K increase in *T*_m_ would increase the population folded from ∼59% at 310K (37°C) to 70%.

Analysis of the nonspecific-binding mode observed at high molar ratios of Sox2 versus FGF4 or random DNA indicates that nonspecific DNA binding involves largely the same residues as in specific DNA binding (Fig. 7E). Compared to the specific interaction mode, nonspecific binding involves fewer residues in the major wing, more residues in helix α3, and larger chemical shift changes for residues at the base of the α3 helix and the early C-terminal tail. This nonspecific interaction could be significantly suppressed by increasing the ionic strength to 500 mM while the specific interaction was unaffected (Supplementary Fig. S20). Since large part of apolar residues that are key to the specific binding are also part of the interface for nonspecific binding (e.g. M49, W51, and Y110), these data suggest that the interface is rather loosely packed in nonspecific binding. These findings are in line with previous work on Sox5 for which it was found that the nonelectrostatic contribution to binding was largest for binding cognate DNA [81]. The nonspecific mode analyzed here occurred at the initial phase of the NMR titration experiment with high Sox2:DNA molar ratio pointing to simultaneous binding of two or more Sox2^31–127^ to the same DNA molecule. To what extent the interface and binding mode characterized here can be extended to other conditions and DNA sequence contexts remains to be established.

**Figure 8.**
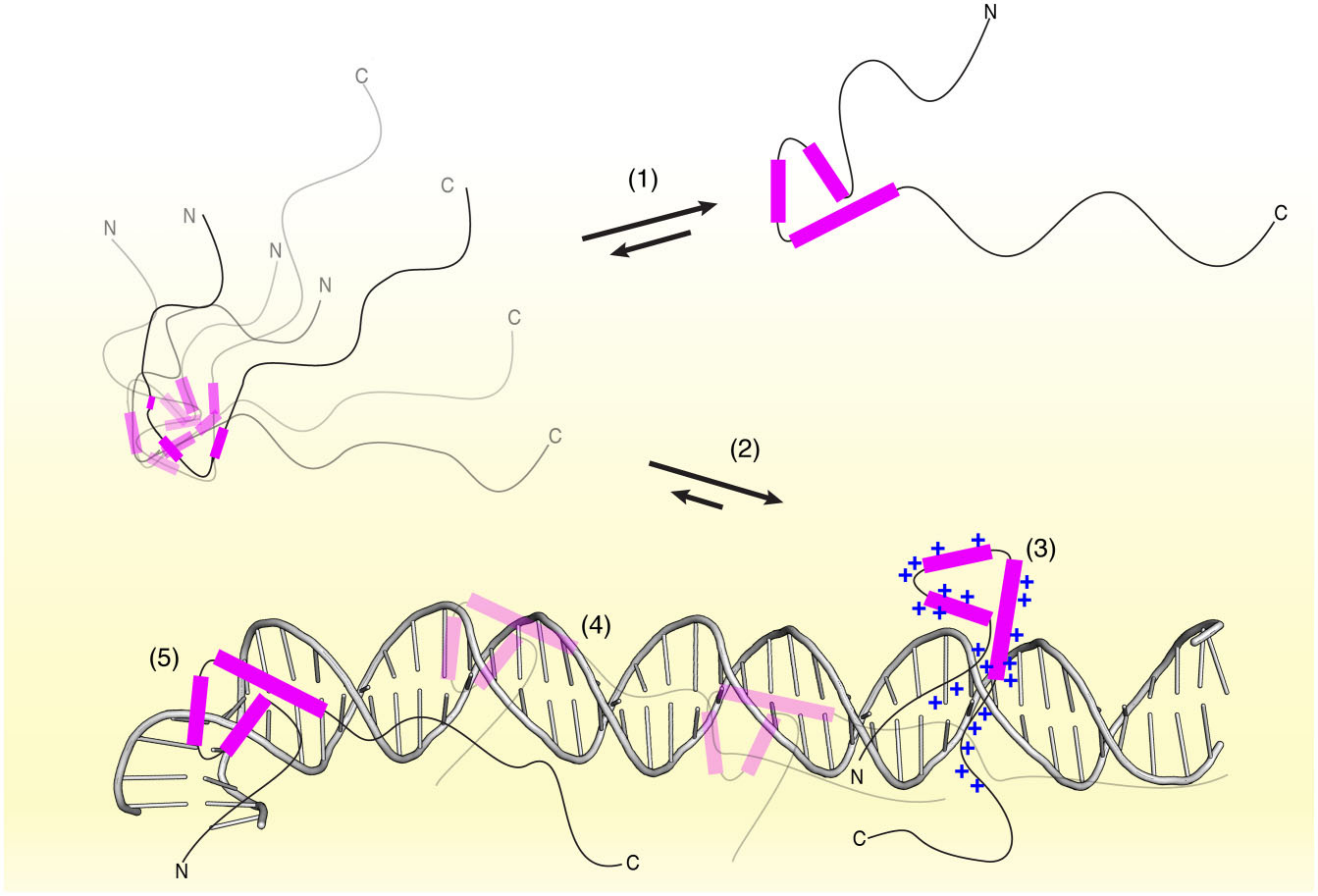
Model for the conformational selection pathway in DNA binding by Sox2. (1) Under physiological conditions, Sox2 exists in an equilibrium between a dominant folded state and unfolded states. (2) Upon approaching the DNA, the folded state is further stabilized by the electric field generated by the DNA (represented by the yellow gradient). (3) Initial nonspecific binding is predominantly electrostatic involving mainly the minor wing and the early C-terminal tail, which facilitate (4) dynamic scanning along the DNA surface. (5) Upon recognition of its cognate binding site, Sox2 binds the DNA in its specific binding mode including the major wing with structural rearrangement and rigidification limited to mostly to the early-middle C-terminal IDR, resulting in DNA intercalation and bending. Alternatively, and not depicted here, unfolded or partially unfolded Sox2 species may also bind via an induced fit pathway (see Supplementary Fig. S23).

We thus come to the following model describing the molecular mechanism of DNA by Sox2–DBD, as depicted in Fig. 8. At physiological conditions and temperatures, unbound Sox2–DBD exists in dynamic equilibrium between a dominant folded (59%) and (partially) unfolded states (41%) (1). The diffusion toward the DNA is likely enhanced by the complementary electrostatics between Sox2–DBD and DNA. The increasing impact of the DNA electrostatics during the approach progressively alleviates electrostatic frustration with the DBD, stabilizing the folded HMG core further before binding occurs (2). DNA binding may thus primarily proceed via conformational selection rather than an induced fit mechanism. Delineation of the exact contribution of conformation selection and induced fit in binding requires detailed knowledge of the kinetic reaction rate constants [86]. Model calculations suggest that even at 310 K (37°C), and without assuming DNA-guided stabilization of the folded state, the flux through conformation selection pathway exceeds that through the induced fit pathway across a range of conditions (Supplementary Fig. S23).

Considering the conformational selection mechanism, our structure shows that the HMG part of the DNA-binding interface is pre-formed, presenting the side chains of key residues for specific DNA binding already in binding competent conformation (Fig. 6D). This process may be facilitated by positively charged residues within the HMG and C-terminal IDR act as anchors that “pull” and orient the DBD toward the DNA [87, 88]. Our titration data revealed a loosely packed nonspecific DNA-binding mode that involved predominantly the HMG minor wing and the early C-terminal tail (Fig. 7E) (3). Once this complex is formed, Sox2 can diffuse along the DNA via one-dimensional scanning to locate its target site [89, 90]. The large overlap between nonspecific and specific binding interfaces is thought to enable a smooth transition from scanning to specific recognition with a low kinetic barrier, as proposed for TF DBDs [91]

Once a cognate binding site is found, the early-middle C-terminal IDR undergoes a disorder-to-order transition supported by specific protein–DNA interactions, resulting stabilization of binding. Such “clamping” role for the C-terminal region has been shown for Sry [92]. How and to what extent these interactions cooperate with the specific interactions in the HMG part of the interface, including the intercalation of M49, remains to be determined. Ultimately, Sox2 bends the DNA with structural rearrangements limited to the N- and C-terminal tails (5). Overall, we thus propose that conformational selection plays a significant role in DNA binding for the Sox2 HMG core and that induced fit is restricted to the conformational changes in the Sox2 N- and C-terminal tails. From the DNA perspective, its conformational change upon Sox2 binding is typically described as induced fit [30] resulting from side chain intercalation and asymmetric charge neutralization by the protein [81].

## Conclusions

Our study provides new insights into the structural dynamics of the Sox2 HMG domain and its DNA-binding mechanism. We showed that the fold of the Sox2 HMG domain is intrinsically encoded in the free state. The folded structure has relatively low thermostability and is in dynamic equilibrium with partially unfolded states. Both DNA binding and increase in ionic strength stabilize the protein. Based on these findings, we propose that conformational selection contributes significantly to DNA binding by Sox2. The population of folded state is dominant at physiological temperature and could further be boosted through stabilization by the DNA electrostatics. As a result, the majority of the DNA-binding interface is pre-formed and ready for interaction. Due to the large overlap between nonspecific and specific DNA-binding interfaces, Sox2 could efficiently transition from a scanning binding mode to the specific binding mode once the target site is reached. At this point, the C-terminal IDR undergoes a disorder-to-order transition, contributing to tighten the binding. Overall, the description of the Sox2–DBD free state and its relevance for DNA binding provided here can form a foundation for further of studies of the Sox2–DNA/chromatin interaction and may be relevant for other pTFs.

## Supplementary Material

gkaf1121_Supplemental_File

## Data Availability

Analysis scripts available upon reasonable request. Chemical shifts of free Sox2^31–127^ are deposited in the Biological Magnetic Resonance Database under accession numbers BMRB ID 34988. The solution structure of Sox2^31–127^ is deposited in the Protein Data Bank under accession code PDB ID: 9QPF.
