# Supplementary material for: Solution structure of the Sox2 DNA-binding domain reveals conformational selection in DNA binding
Source: Nucleic Acids Res. 2025 Nov 4;53(20):gkaf1121. doi: 10.1093/nar/gkaf1121 (PMC12585912; doi:10.1093/nar/gkaf1121)
Supplement: gkaf1121_Supplemental_File [file gkaf1121_supplemental_file.pdf]

Supplementary Information

for

Solution structure of the Sox2 DNA binding domain reveals conformational selection in DNA binding

Andrea Orsetti[1], Jonathan Slejfer[1], Satine Ha[1], Damian I. Kevelam[1], Jan Tekkelenburg[1], Tjitske van Duijn, Anni Leppäkoski, Aren Sedrakyan, Akos Szilagyi[2], Raymond D. Schellevis[1] , Abdenour Soufi[3], Vlad Cojocaru[1,2] Hugo van Ingen[1]

[1] NMR Spectroscopy group, Bijvoet Centre for Biomolecular Research, Utrecht University, Utrecht, The Netherlands

[2] STAR-UBB Institute & Doctoral School for Integrative Biology, Babeș-Bolyai University, Cluj-Napoca, România

[3] Centre for Regenerative Medicine, Institute for Regeneration and Repair, The University of Edinburgh, Edinburgh, United Kingdom

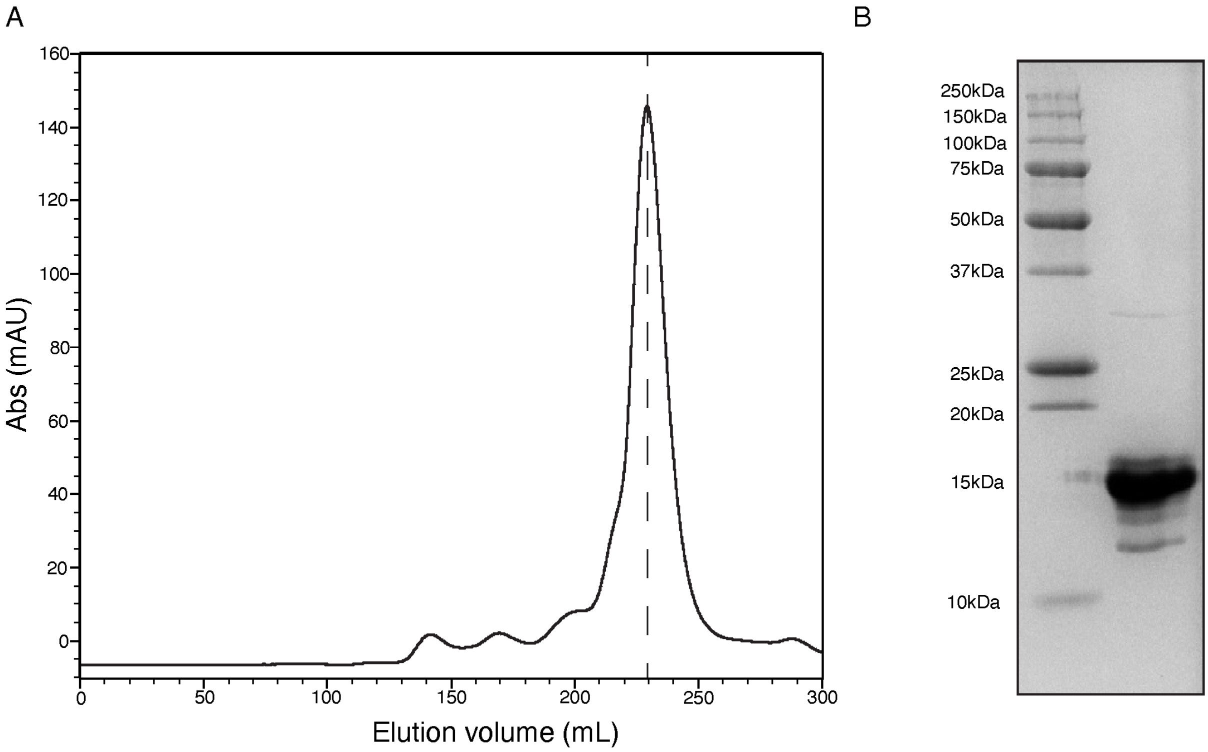

Figure S1. Sox2$^{31\text{-}127}$ purification. (A) Size-exclusion chromatogram of Sox2$^{31\text{-}127}$ using a HiLoad 26/60 Superdex 75 pg column at a flow rate of 2mL/min. The dashed line indicates an elution volume of 230 mL. (B) SDS-PAGE (10% polyacrylamide) showing the apparent molecular weight marker (left) and purified Sox2$^{31\text{-}127}$ (right), the final yield of soluble Sox2$^{31\text{-}127}$ both unlabeled and isotope labeled was ~20 mg/L of culture, with a A_{260}/A_{280} of 0.5-0.6.

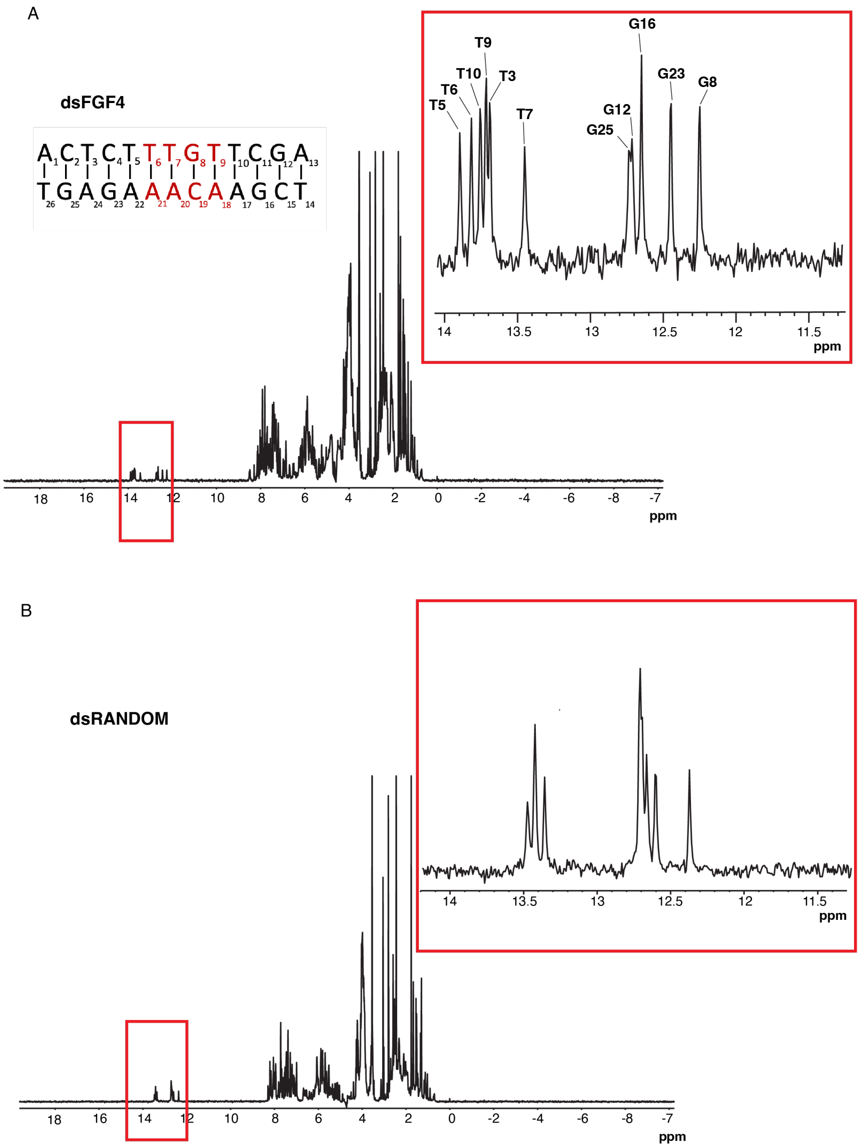

Figure S2. One-dimensional ^{1}H NMR spectra of the double-stranded DNAs used in this study, recorded at 293 K in 10 mM potassium phosphate buffer (pH 7.0).(A) Spectrum of FGF4 DNA (dsFGF4) with inset highlighting the imino proton region (12–14 ppm), which reflects hydrogen-bonded base pairs. Assignments were derived from a ^{1}H^{1}H NOESY experiment under the same conditions.(B) Spectrum of the random DNA, lacking the TTGT motif (dsRANDOM) also highlighting the imino proton region (12–14 ppm), confirming the presence of stable Watson-Crick base pairing.

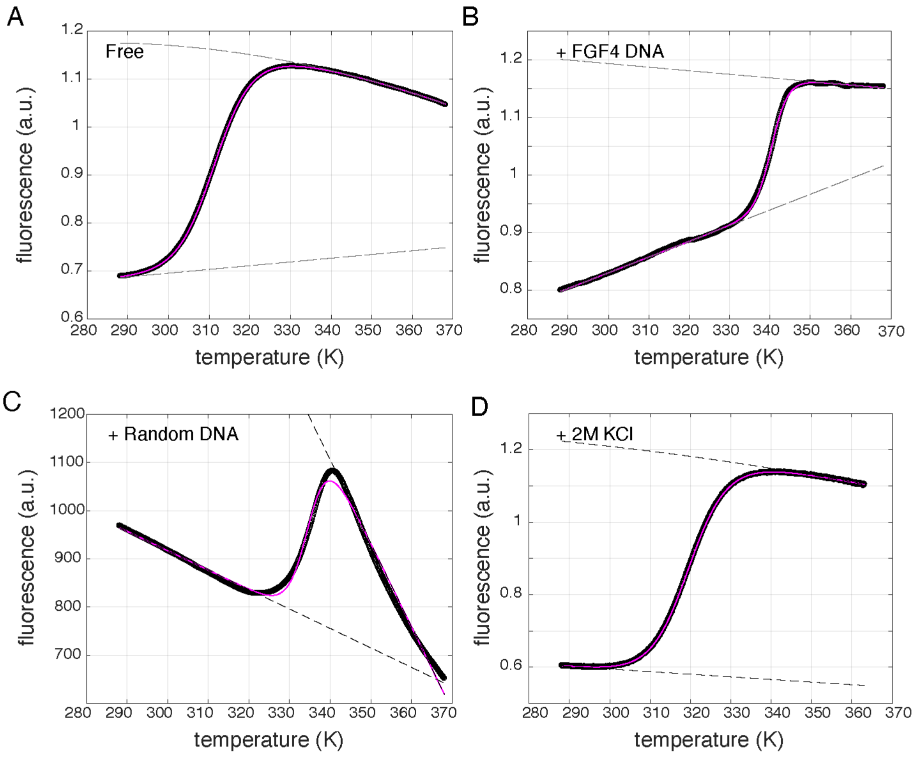

Figure S3. Raw melting curves and fits for (A) free,(B) FGF4 DNA bound, (C) random DNA bound Sox2$^{31\text{-}127}$ (all in physiological-like buffer: 20 mM Tris, 135 mM KCl, 15 mM NaCl, pH 7.3) and (D) free Sox2$^{31\text{-}127}$ in physiological-like buffer plus 2 M KCl. Raw data in black, fitted curve in magenta and fitted baseline in folded and unfolded state shown as dashed gray line. Fit parameters free state: T_m: 311.7 (K), ΔH_m: 176 (kJ/mol), ΔC_p: 0.019 (kJ/mol/K), aN0: 0.674 (a.u.), aU0: 1.17 (a.u.), gN : 0.000781 (K^{-1}), g1U: 0.000312 (K^{-1}), g2U: –1.73e-05 (K^{-2}); FGF4 bound state: T_m: 340.6 (K), ΔH_m : 472 (kJ/mol), ΔC_p: 0 (kJ/mol/K), aN0: 0.756 (a.u.), aU0: 1.21 (a.u.), gN: 0.00273 (K^{-1}), g1U: –0.000621 (K^{-1}), g2U: 0 (K^{-2}); random DNA bound state: T_m: 335.2 (K), ΔH_m : 375 (kJ/mol), ΔC_p: 0.101 (kJ/mol/K), aN0: 1030 (a.u.), aU0: 2270 (a.u.), gN: –4.4 (K^{-1}), g1U: –17.3 (K^{-1}), g2U: 0 (K^{-2}); 2 M KCl: T_m: 319.9 (K), ΔH_m: 179 (kJ/mol), ΔC_p: 0.0 (kJ/mol/K), aN0: 0.615 (a.u.), aU0: 1.28 (a.u.), gN : –0.000777 (K^{-1}), g1U: –0.0019 (K^{-1}), g2U: 0.0 (K^{-2}) where T_m is the melting temperature, ΔH_m the enthalpy of unfolding, ΔC_p the change in heat capacity of unfolding, and aN0, gN are parameters for the linear baseline of the native, folded state and aU0, g1U and g2U are parameters for the quadratic baseline of the unfolded state.

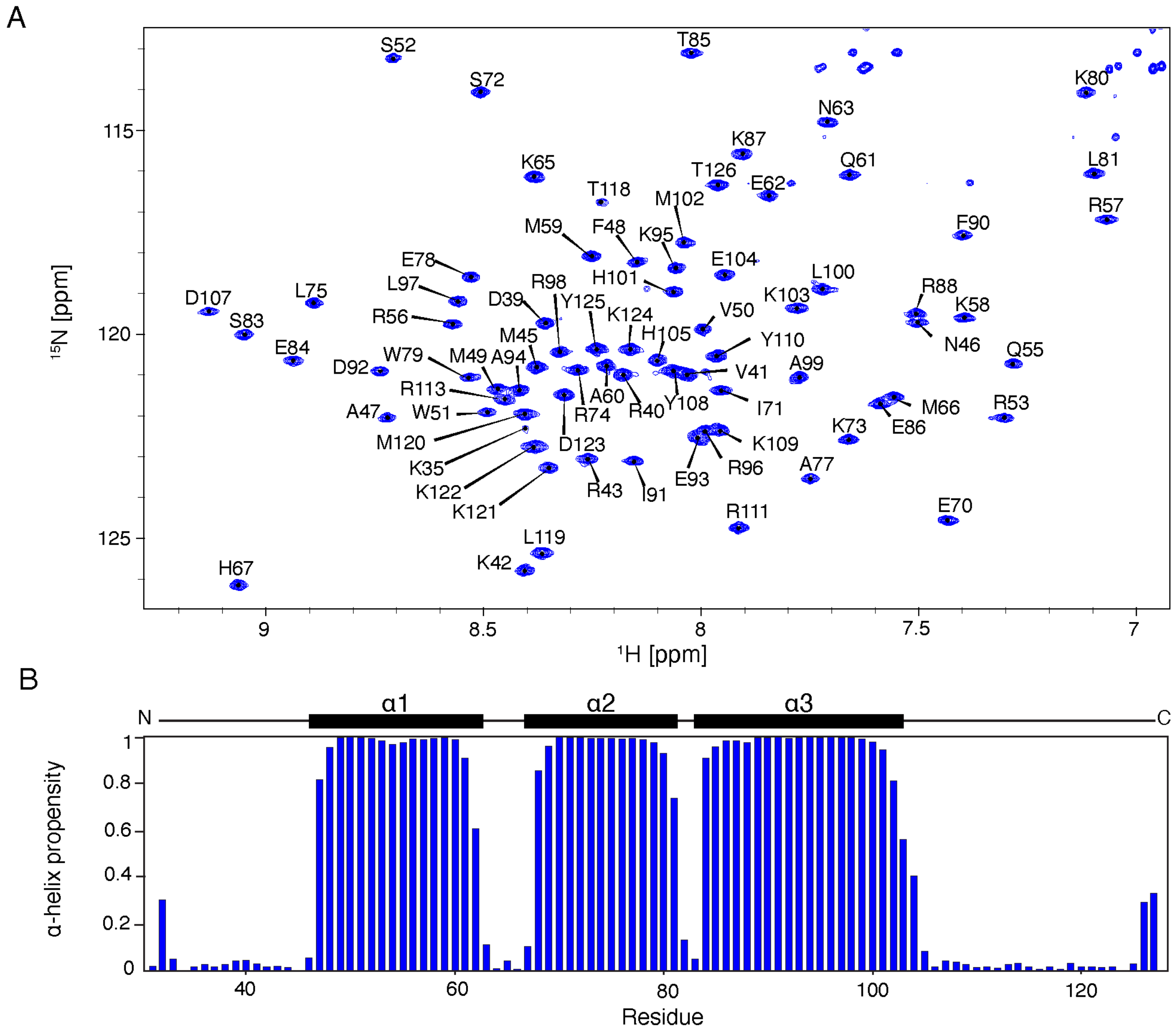

Figure S4: (A) ^{15}N-TROSY of Sox2$^{31\text{-}127}$ in physiological-like buffer recorded at 900MHz with a temperature of 293K. (B) Secondary structure determination of Sox2$^{31\text{-}127}$ using TALOS-N showing the α-helix propensity as a score between 0 and 1 [1].

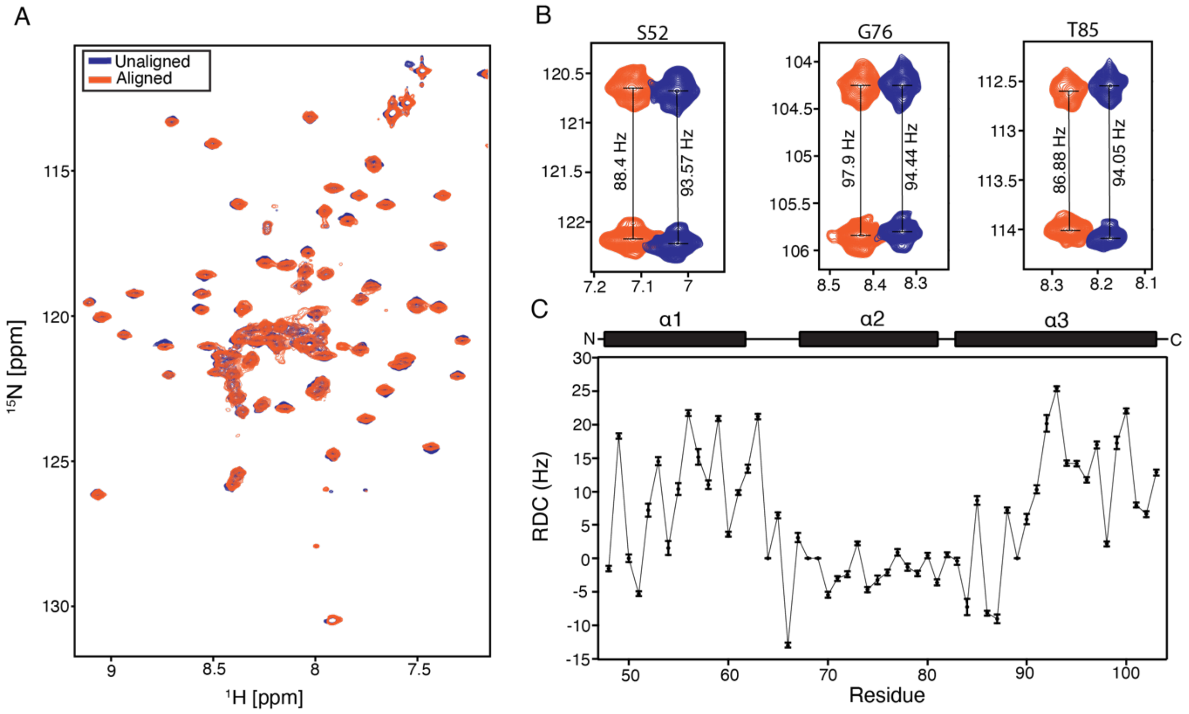

Figure S5. Backbone amide RDCs of Sox2$^{31\text{-}127}$ (A) ^{15}N-HSQC of Sox2$^{31\text{-}127}$ in both unaligned and aligned states, demonstrating consistent peak positions across both conditions. (B) Overlay of selected peaks from three different helical regions, comparing the unaligned (blue) and aligned (orange) ^{15}N HSQC-IPAPs of residues within the three helices. J_{NH} (unaligned) and $J_{NH,app}$ (aligned) are displayed beside the splittings. (C) RDCs values plotted as a function of residue number, illustrating the dipolar wave trend within the helices.

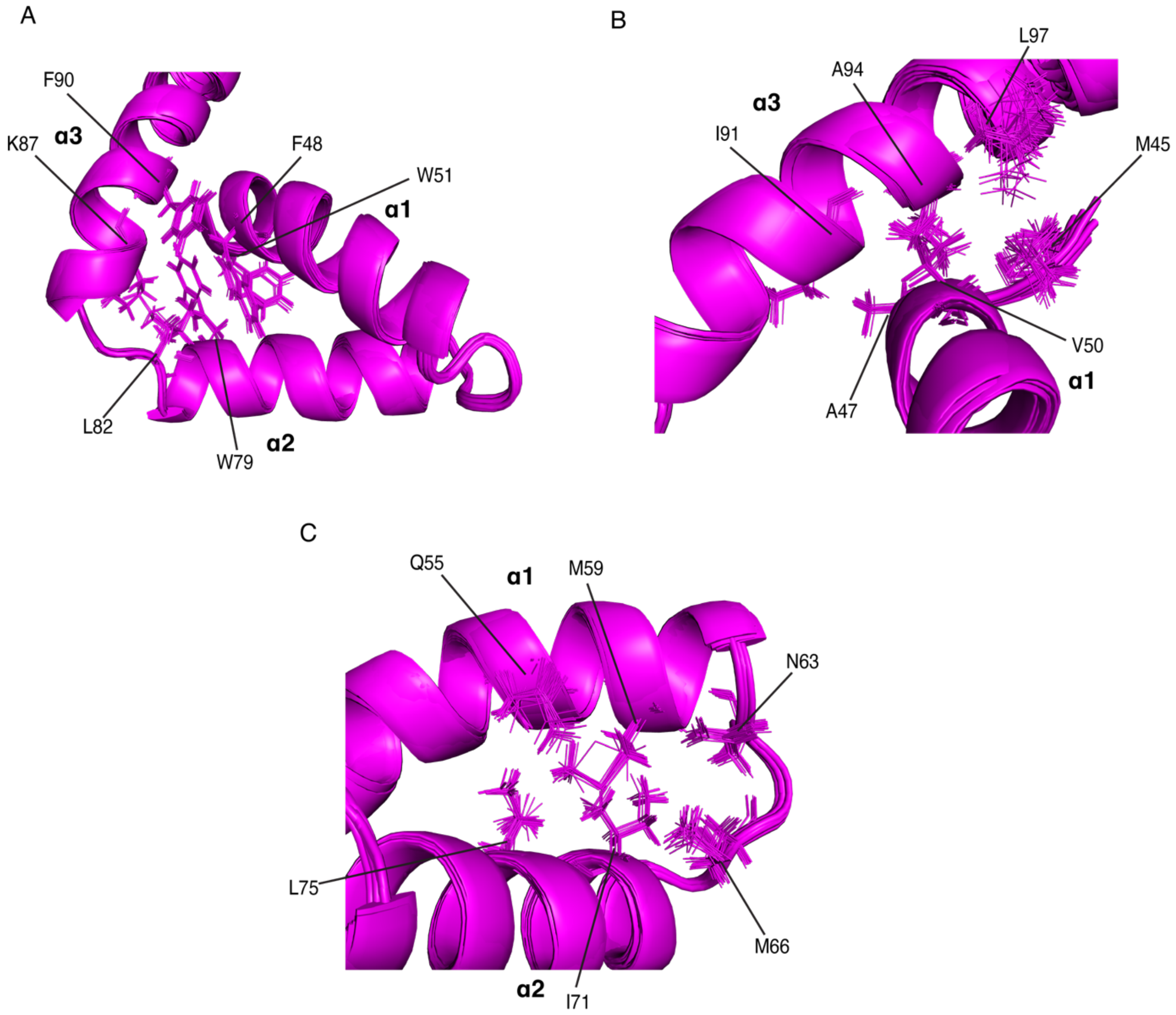

Figure S6. Hydrophobic cores in Sox2$^{31\text{-}127}$: primary hydrophobic core (A), second hydrophobic cluster between residues within the beginning of helix α1 and the middle of helix α3 (B), and third hydrophobic cluster located in between helix α1 and α2 (C).

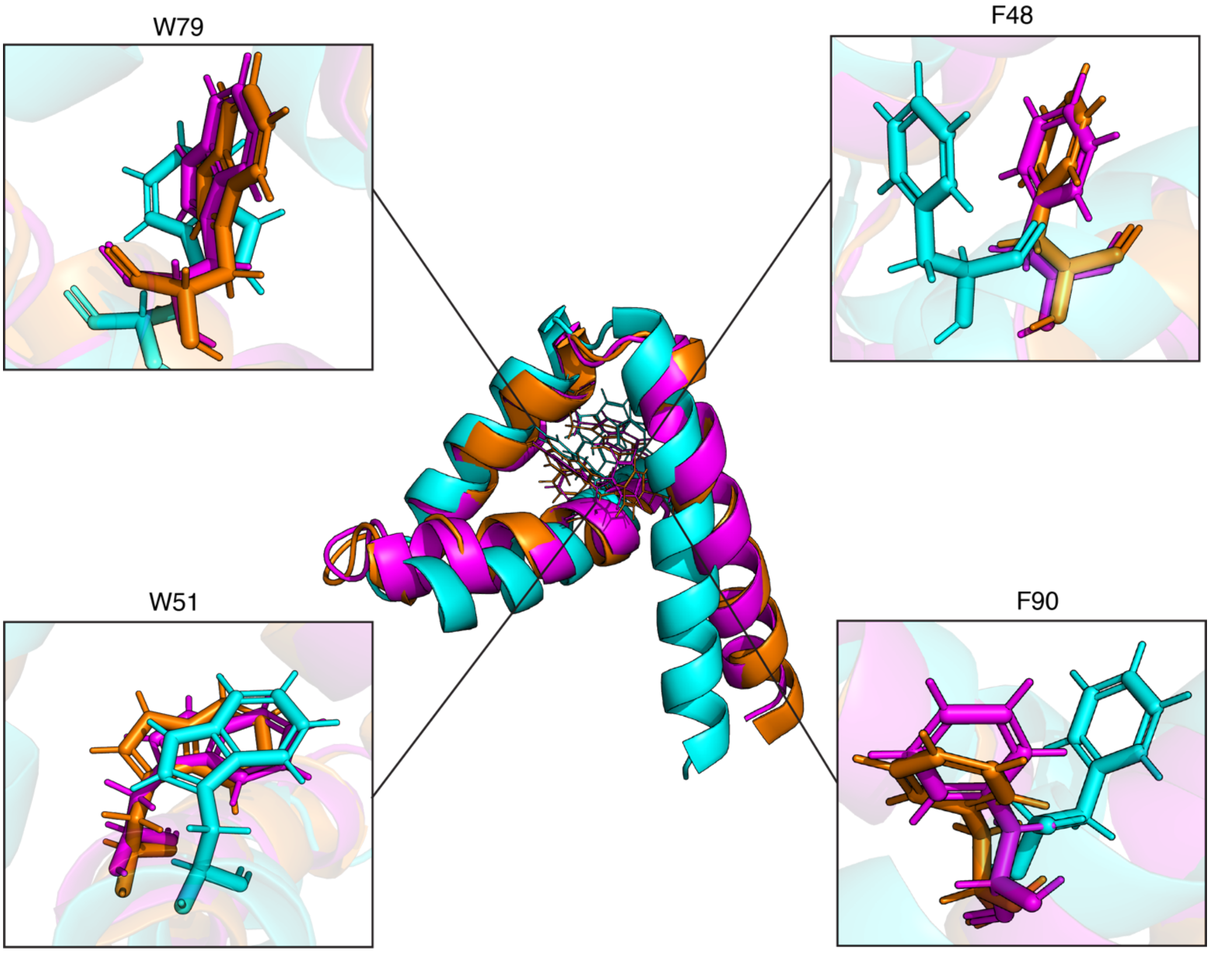

Figure S7. Structural alignment (47-104) of Sox2$^{31\text{-}127}$ (magenta), the free-state Sox2-DBD from PDB 2LE4 (cyan), and the DNA-bound Sox2-DBD from PDB 1GT0 [2] (orange). Four close-up views highlight the side chain orientation of aromatic residues within the hydrophobic core. As shown, Sox2$^{31\text{-}127}$ and 1GT0 exhibit similar orientations of these residues, while 2LE4 deviates significantly. Notably, the W79 side chain forms a hydrogen bond, via the NH group in the indole ring, to the DNA in the bound state. Clearly, while W79 is in a DNA-binding competent state in Sox2$^{31\text{-}127}$, it is not in 2LE4 where the side chain is rotated such that the NH group is facing the protein interior.

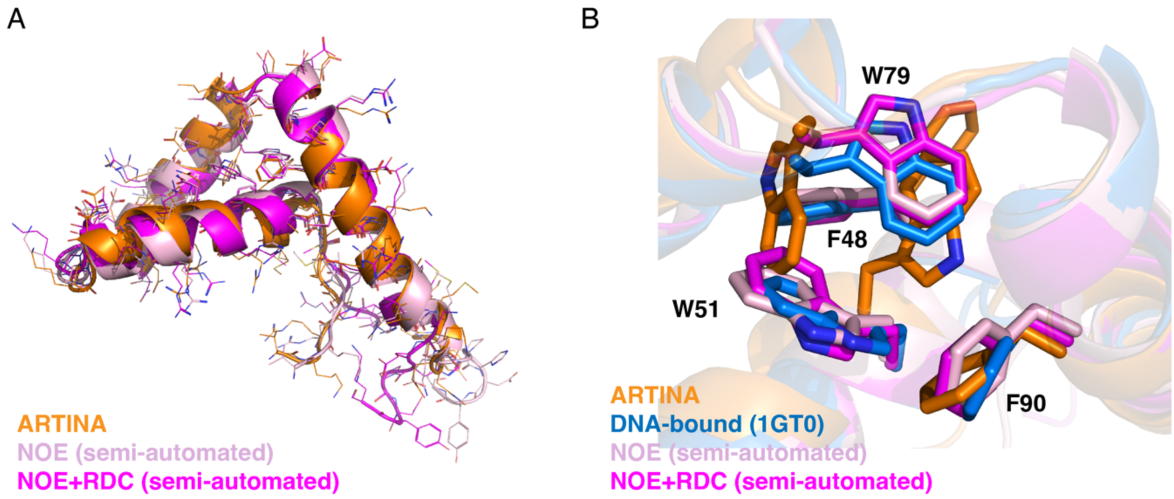

Figure S8. Comparison of manually curated and ARTINA structure of Sox2$^{31\text{-}127}$. (A) Superposition of 1st structure proposal from a fully automated structure determination by ARTINA [3] and the structures resulting from the semi-automated, manually curated determination either based on NOESY data only, or on NOESY and RDC data. The heavy atom backbone RMSD for the folded HMG core (residues 47-104) is 1.31 Å between the ARTINA and NOE only structure. (B) Zoom on the aromatic core with key side chains shows as sticks and labeled. In the ARTINA run assignment of W51 and W79 HH2 are swapped which together with overall lower assignment completeness for aromatic residues results in very different side chain configuration in the ARTINA structure.

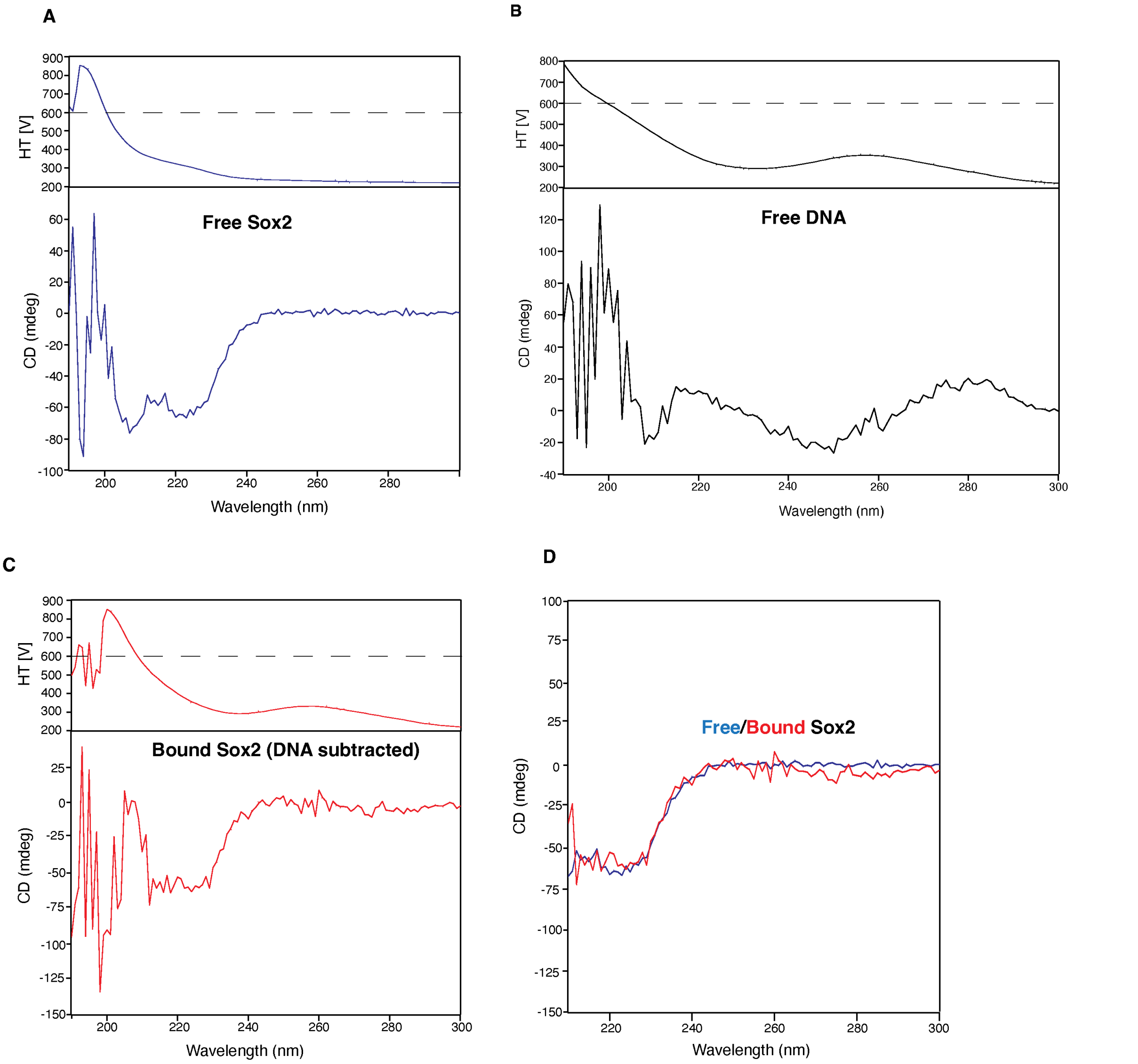

Figure S9. CD spectra of free Sox2$^{31\text{-}127}$ (A), free FGF4 DNA (B), bound Sox2$^{31\text{-}12}$ (free DNA spectrum subtracted from the complex spectrum) (C) and overlay of free and bound Sox2$^{31\text{-}127}$ (D), all recorded at 293 K in 10 mM NaPi, pH 7.3 on a JASCO J-810 CD spectropolarimeter in 1 mm pathlength rectangular quartz cell. Protein concentration 40 µM, protein and DNA mixed in 1:1 molar ratio to obtain complex. Only data acquired at high-tension (HT) voltages below 600 V were included in the final analysis in panel D to ensure spectral reliability.

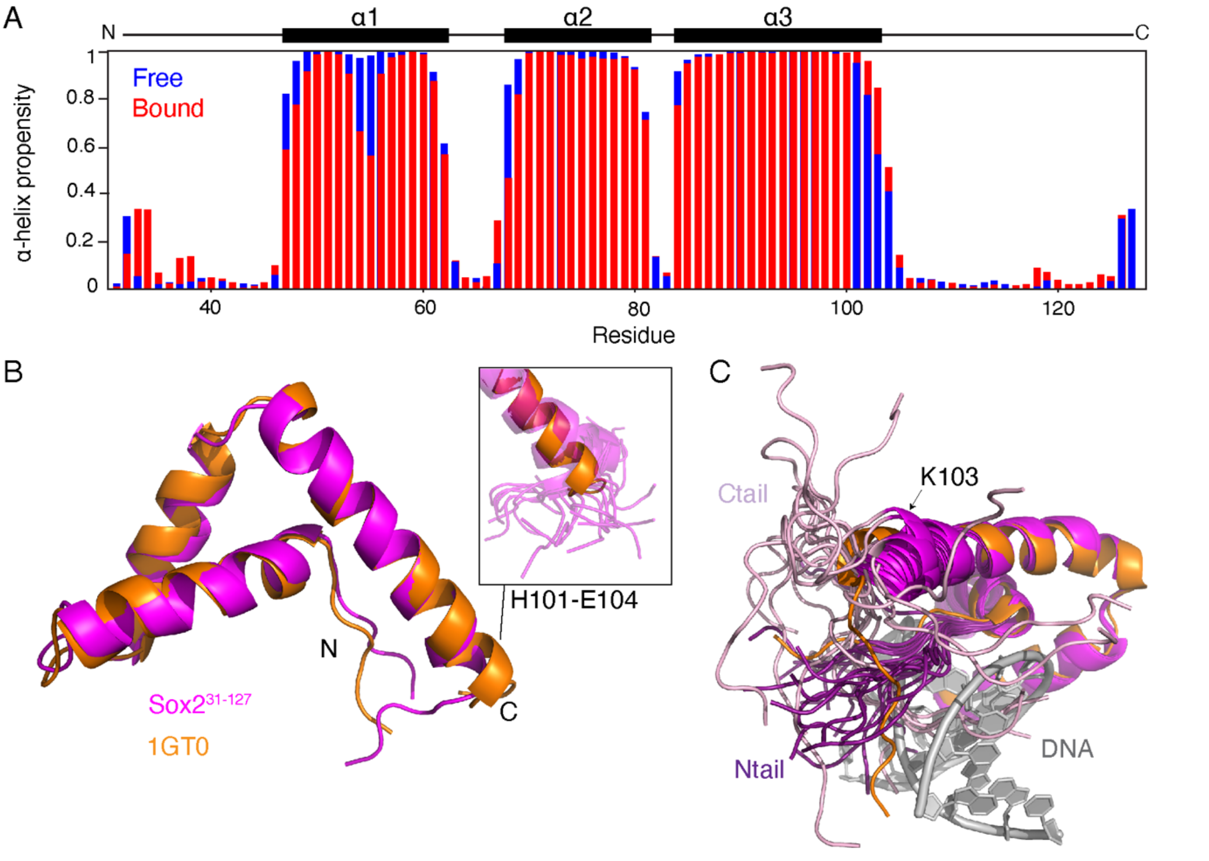

Figure S10. Comparison of free and bound Sox2 DBD structure. (A) Secondary structure determination of free (blue) and FGF4-DNA bound (red) Sox2$^{31-127}$ using TALOS-N showing the α-helix propensity as a score between 0 and 1. (B) Structural alignment of free Sox2$^{31-127}$(magenta) with the FGF4 DNA bound state (PDB:1GT0) (orange). Inset highlights the loose definition of the final turn of the of α3 helix (L100–E104) in the free state. (C) Comparison of the N- and C-terminal tails of the Sox2 HMG domain in free and DNA-bound states. Color coding indicated.

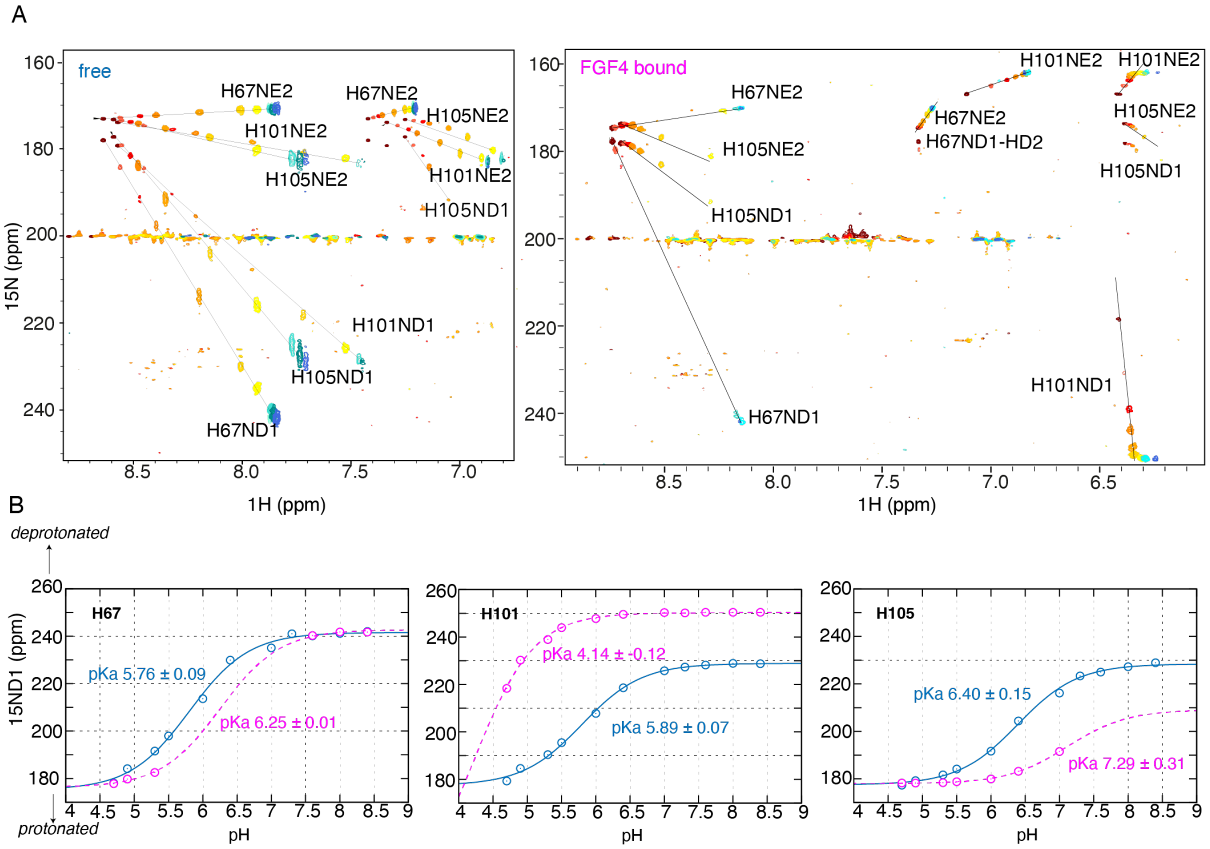

Figure S11. Determination of histidine side chain pKa values in free and FGF4-DNA bound Sox2[31-127]. (A) Overlay of ^2J 1H-15N HMQC spectra[6] recorded between pH 4.7 (dark red) and 8.4 (blue). Data for free Sox2 on left and DNA-bound on right. Peak trajectories indicated. In the DNA-bound state the trajectory for H101-HE1 is distinctly curved at high pH (>7), indicative of additional conformational changes. Analysis of peak patterns according to Pelton et al. [4] revealed that all histidines assume Nε1 tautomer in the deprotonated state (i.e. the Nδ1 is deprotonated). (B) Chemical shift of the Nδ1resonance as function of pH for H67, H101 and H105 in free and DNA-bound state; high ppm values are indicative of the deprotonated state. Best fit lines and average pKa determined from independent fits of all cross-peaks that could be tracked are shown; error bar is given by the standard deviation of the average pKa over these individual fits. All buffers were controlled to 150 mM ionic strength.

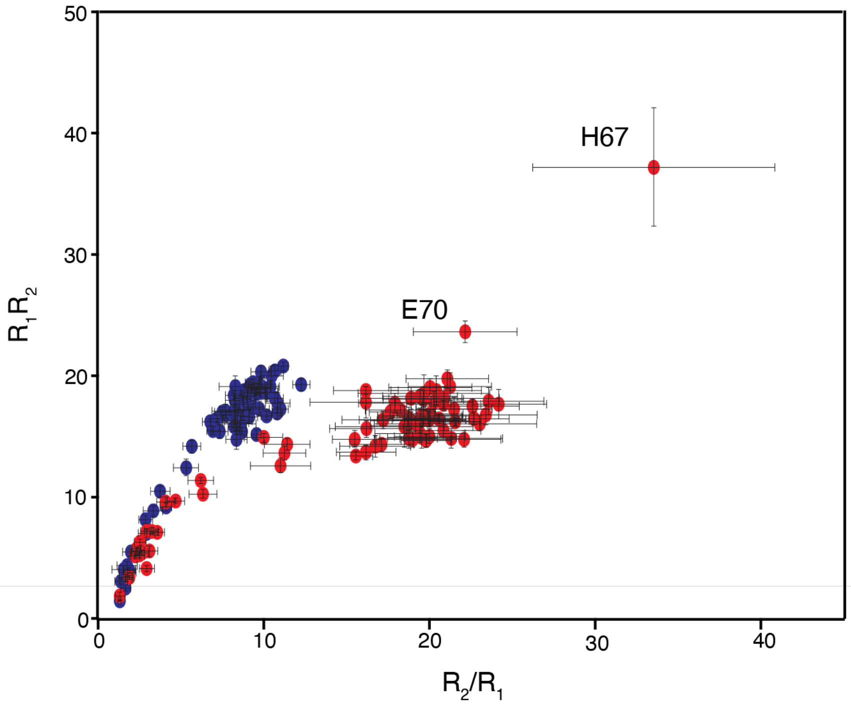

Figure S12. Backbone amide ^{15}N relaxation data for $Sox2^{31\text{-}127}$ are displayed as R_1R_2 vs R_2/R_1. The two datasets refer to $Sox2^{31\text{-}127}$ free (blue) and $Sox2^{31\text{-}127}$ FGF4 DNA bound (red). For both free and DNA-bound $Sox2^{31\text{-}127}$, an axially symmetric diffusion tensor yielded a statistically significantly better fit than an isotropic diffusion model in the modelfree analysis.

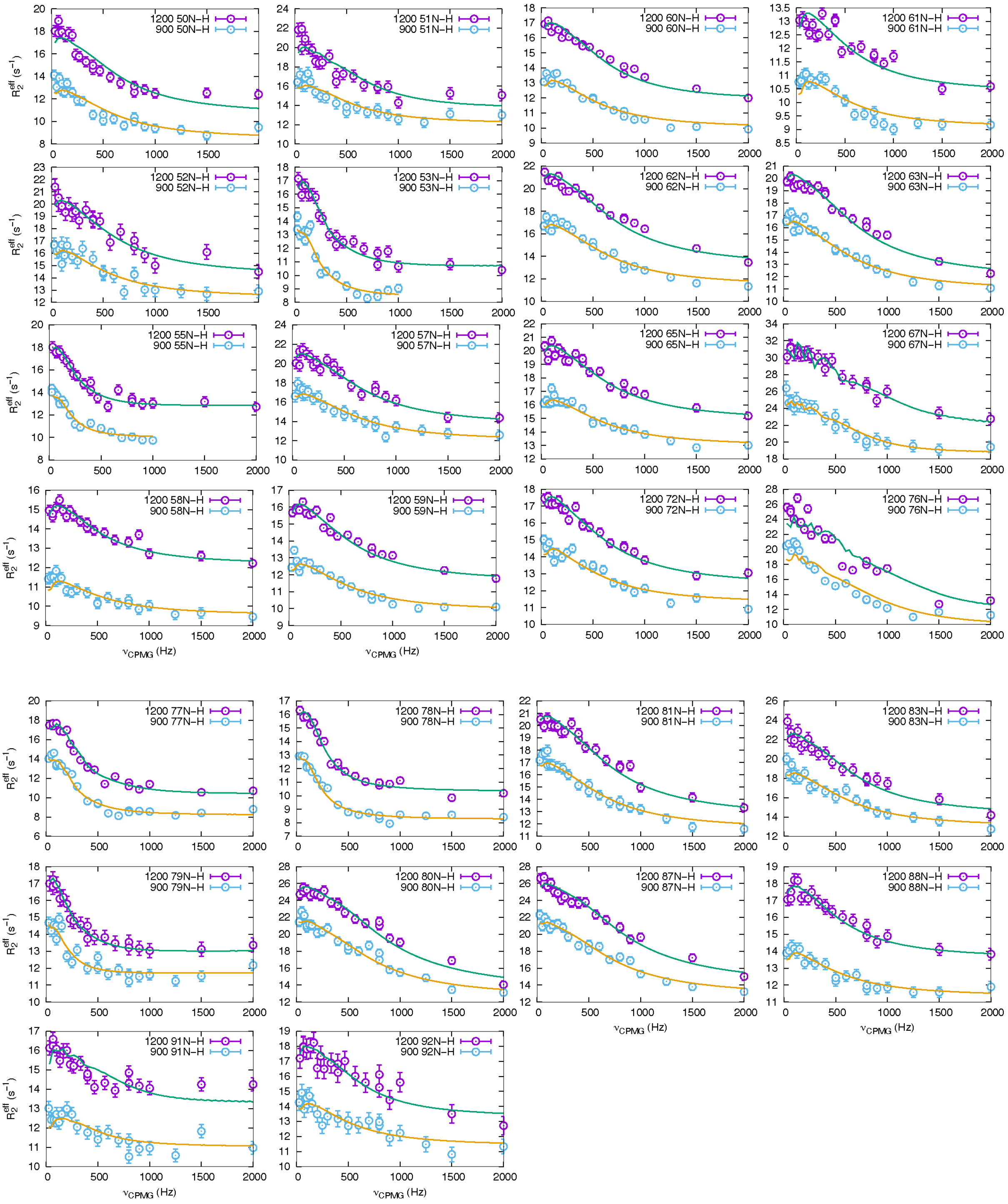

Figure S13. Experimental CPMG data and fits from ChemEx for free Sox2[31-127]. Note that for residues R53 and Q55 the 900 data was truncated at 1000 Hz maximum CPMG frequency due to data abnormalities at higher pulsing rates. Fitted parameters are listed in Table S3.

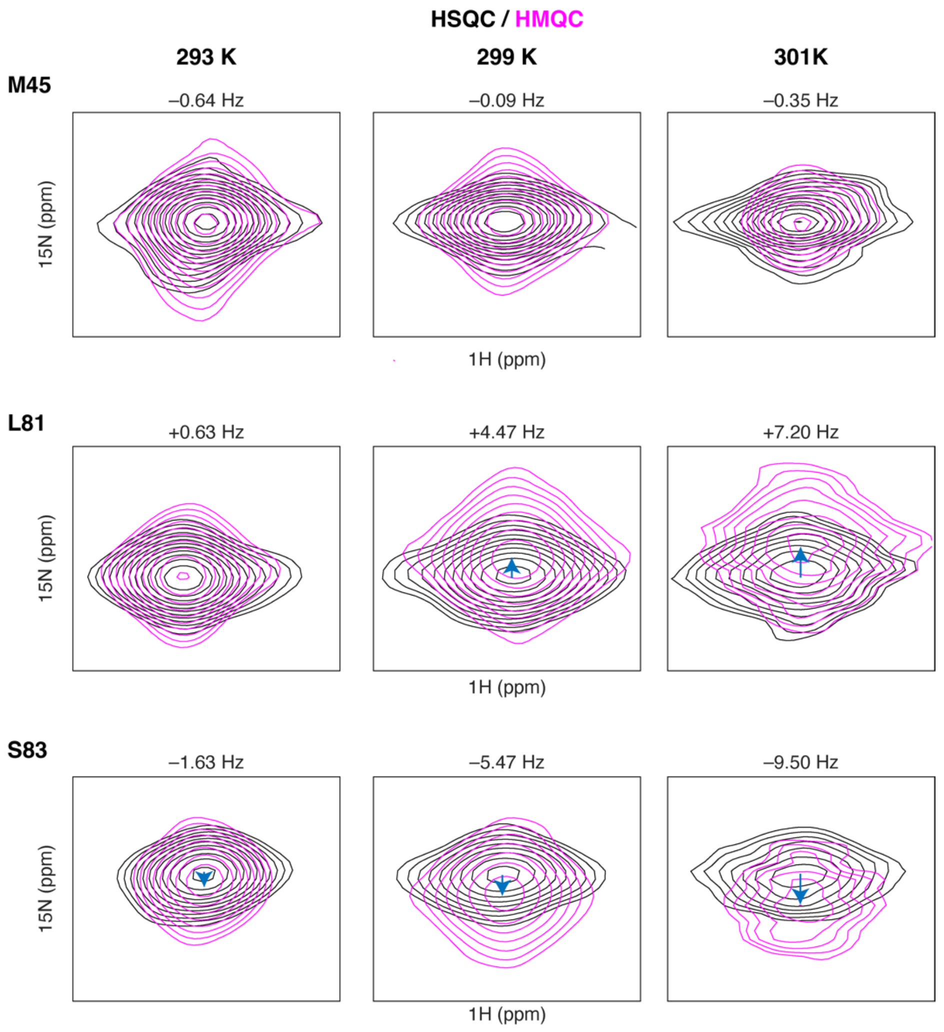

Figure S14. Comparison of ^{1}H-^{15}N HSQC (black) and HMQC (magenta) spectra of free-state Sox2$^{31\text{-}127}$ for the determination of the sign of the chemical shift difference between ground and excited state. For residue M45 no significant difference in peak position is obtained at either 293, 299 or 301 K (peak position difference in Hz listed above each panel). This matches the absence of significant dispersion of $R_{2,eff}$ values in ^{15}N CPMG relaxation dispersion experiments. For residues L81 and S83 significant dispersion had been found ($\Delta\omega_{GE}$ 3.6 and 3.7 ppm). At 299 K a clear difference can be observed while at 293 K this difference is just visible. At 301 K the peak position difference is further amplified at costs of extensive line broadening, in particular for the HMQC spectrum. Importantly, the relative difference in peak position is the same across the different temperatures.

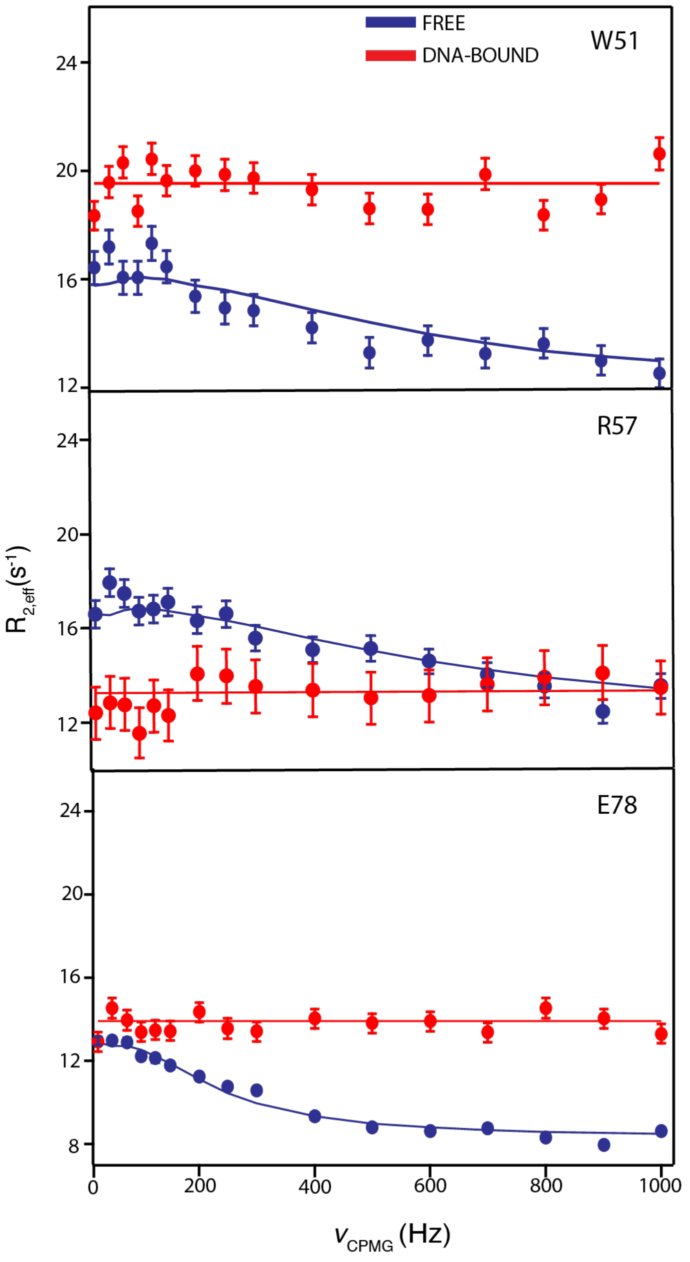

Figure S15. Comparison of ^{15}N-CPMG dispersion plots between free and DNA bound Sox2$^{31-127}$ recorded at 900 MHz ^{1}H frequency. The data show a quench of the dispersion in the DNA bound state.

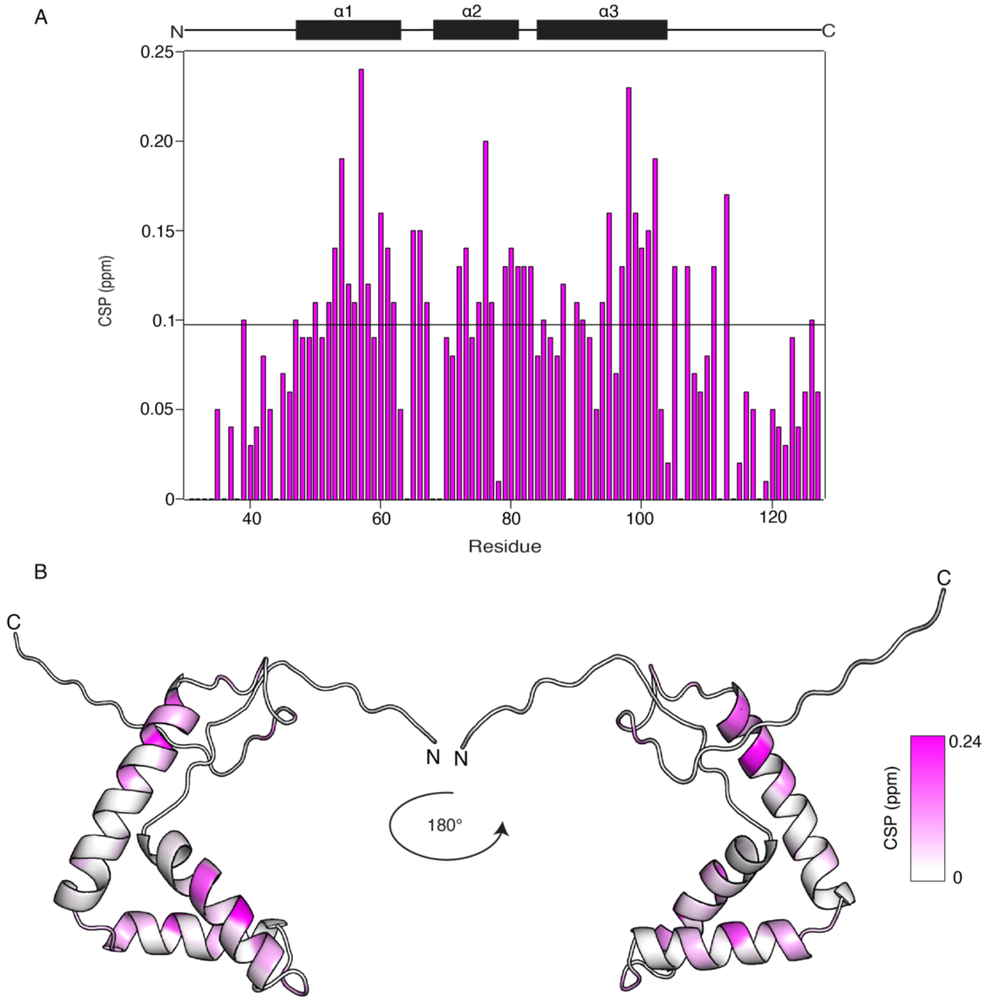

Figure S16. Chemical shift perturbation of Sox2$^{31-127}$ upon KCl titration (A) Plot showing chemical shift perturbations (CSPs) of Sox2$^{31-127}$ as the KCl concentration increases from 135 mM to 2 M. The significance threshold, set at twice the standard deviation of all CSP values (0.97 ppm), is indicated by the horizontal black line. Residues with CSPs above this threshold are considered significantly affected by KCl.(B) CSPs mapped onto the 3D structure of Sox2$^{31-127}$. Color coding indicated in the Figure.

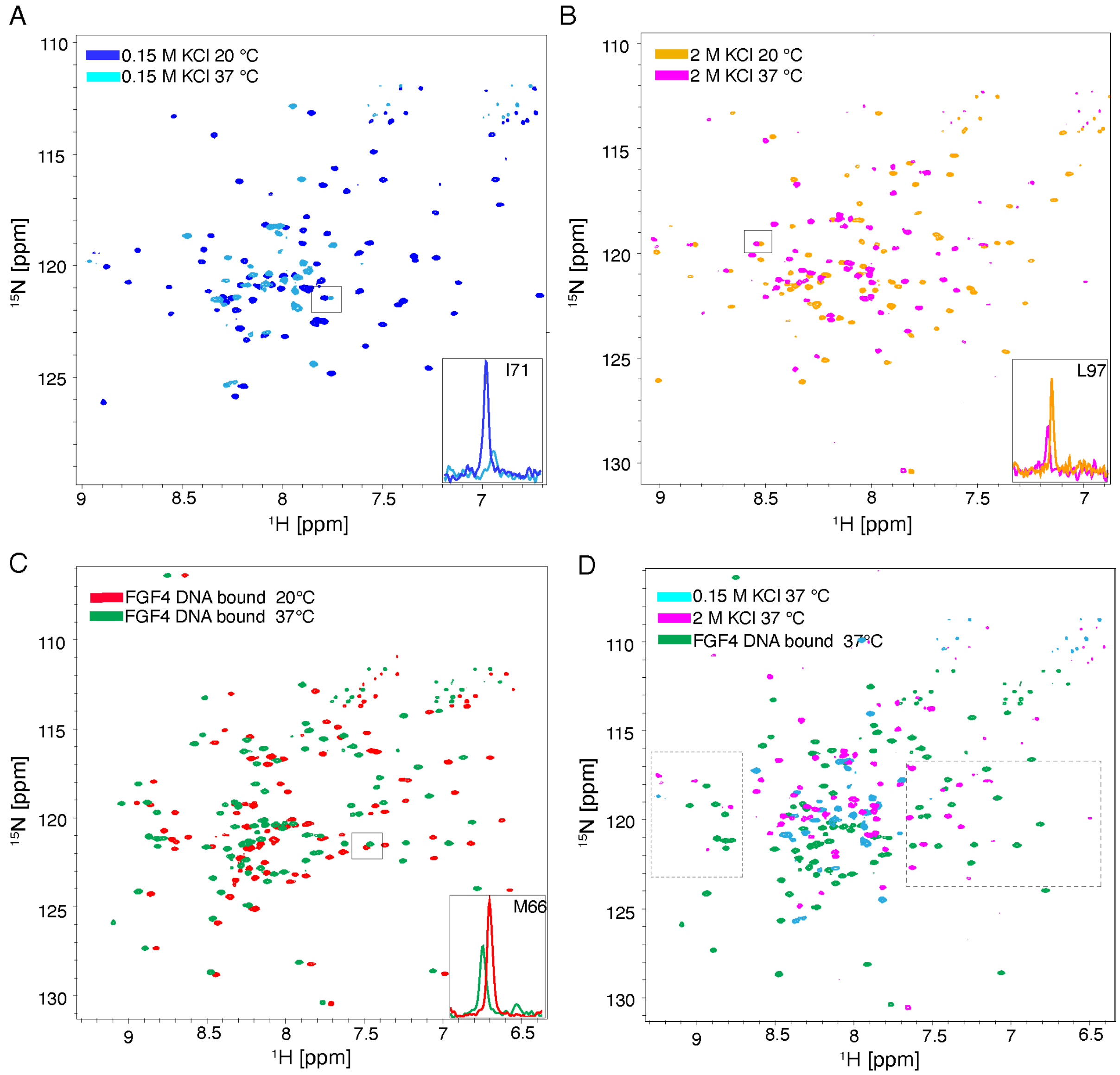

Figure S17. Comparison of ^{15}N-TROSY spectra of Sox2$^{31\text{-}127}$ recorded at 900 MHz and 293 K or 310 K in physiological-like buffer (A), with 2 M KCl (B), with 1 molar equivalent FGF DNA (C). Overlay of three condition at 310 K shown in panel (D). (A) The 310 K condition shows a severe decrease in signal intensity and chemical shift dispersion, indicative of partial unfolding compared to the 293 K condition. A 1D trace of residue I71 in α-helix 2 illustrates the drop of about 80% in intensity at 310 K.(B) The 310 K spectrum shows a moderate decrease in intensity but retains good chemical shift dispersion similar to the 293 K condition. A 1D trace of residue L97 in α-helix 3 shows the moderate intensity reduction of about 30% at 293 K . (C). The 310 K condition displays a moderate decrease in intensity while retaining good chemical shift dispersion comparable to 293 K. A 1D trace of residue M66 in α-helix 1 shows a moderate intensity drop of about 40% between 293 K and 310 K (bottom left).

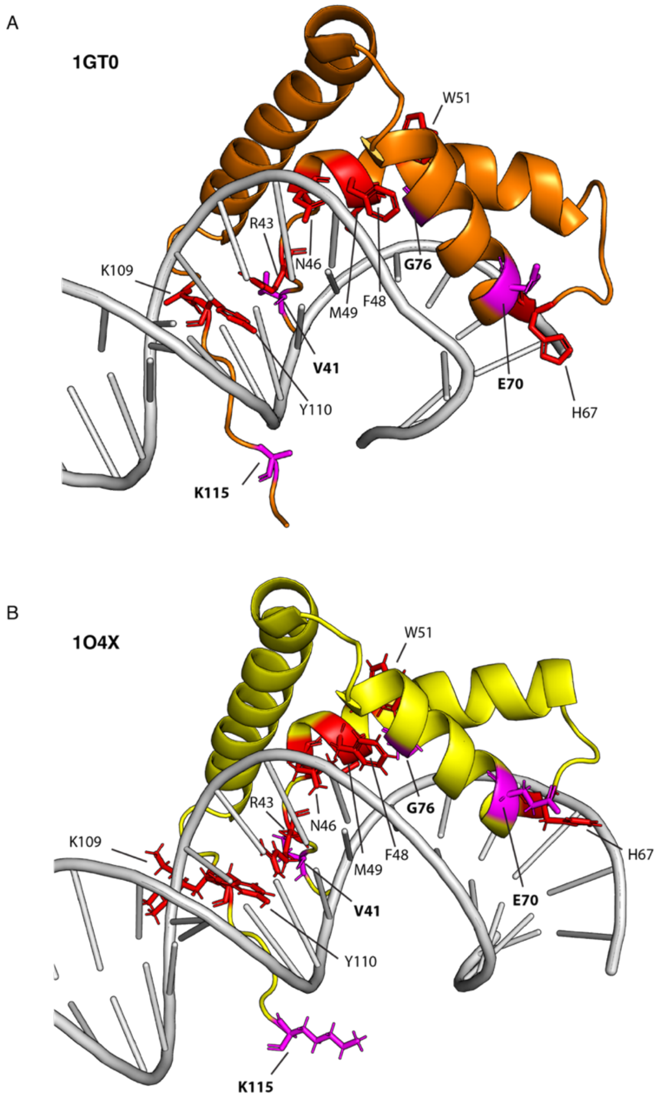

Figure S18. Chemical shift perturbation (CSP) mapping onto the structure of the Sox2-HMG-DNA complexes PDB 1GT0 (A) and PDB 1O4X (B). Residues previously identified as crucial for specific DNA interactions are highlighted in red. Residues with significant CSP that are additional to this previously identified interface, are marked as magenta and the residues labels in black bold. In particular V41 could interact with H105/Y110 as suggested by Weiss, whereas G76 is position on top of the DNA backbone explaining the observed CSP. For E70 the effect is likely indirect due to the critical region N68-S69. Instead K115 could interact with the DNA as indicated in 1O4X [2, 5].

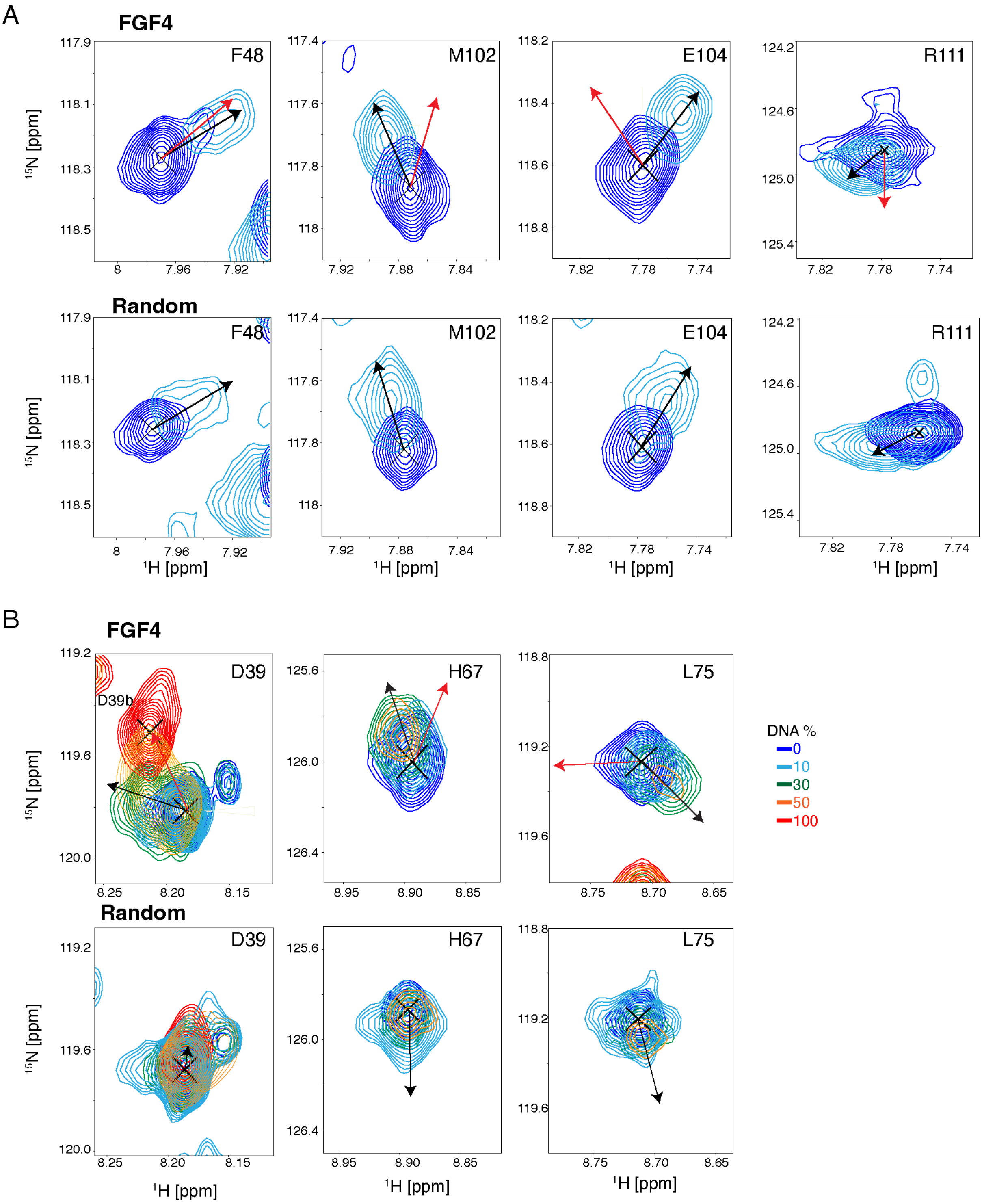

Figure S19. Comparison of peak trajectories in ^{15}N-TROSY spectra of Sox2$^{31\text{-}127}$ with either FGF4 or random DNA , illustrating (A) similarities or (B) differences in initial peak trajectories. Red arrows indicate trajectories to the fully bound state (100%) for the FGF data.

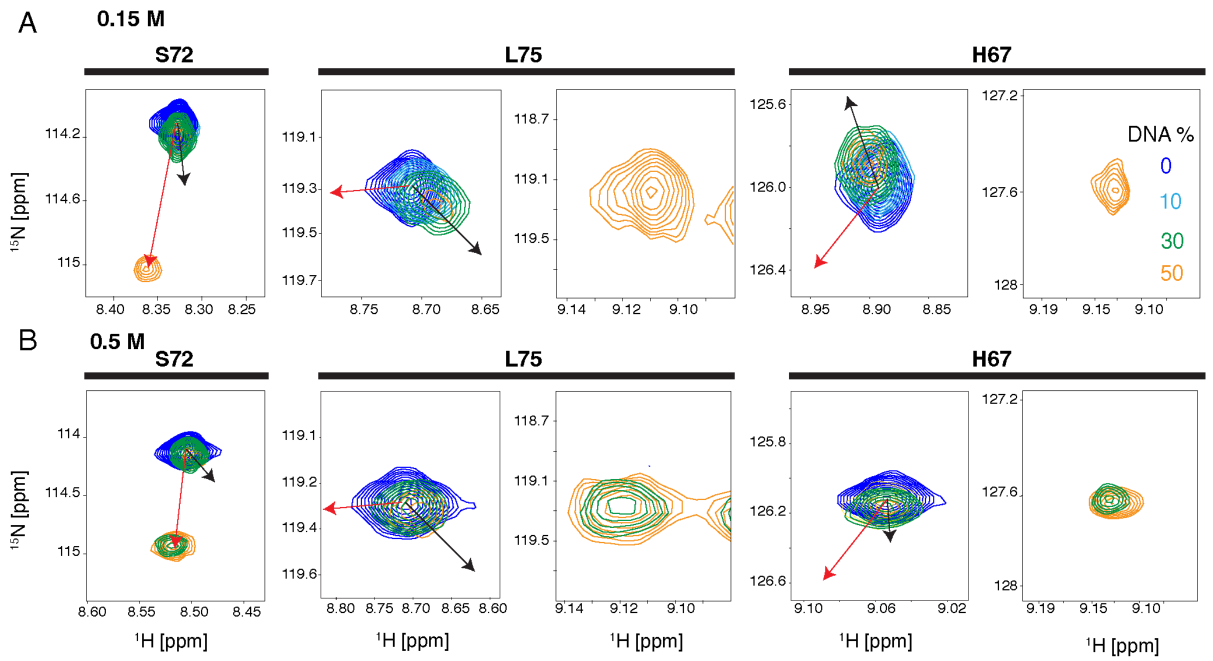

Figure S20. Comparison of peak trajectories in ^{15}N-TROSY spectra of Sox2-DBD during titration with FGF4 DNA at either (A) 0.15 M and (B) 0.5 M ionic strength. For L75 and H67, free state peak position at the left, bound-state peak position on the right. At 30% DNA added, peaks close to the free and the final bound state that are in slow exchange can be seen at 0.5 M, whereas at 0.15 M only a peak close to the free state with CSP in fast exchange can be observed. This indicates that increasing ionic strength promotes the final bound state by reducing non-specific electrostatic interactions. Data at 0.5 M was recorded using a different Sox2-DBD construct, spanning residues 24 to118 with an N-terminal T7-tag.

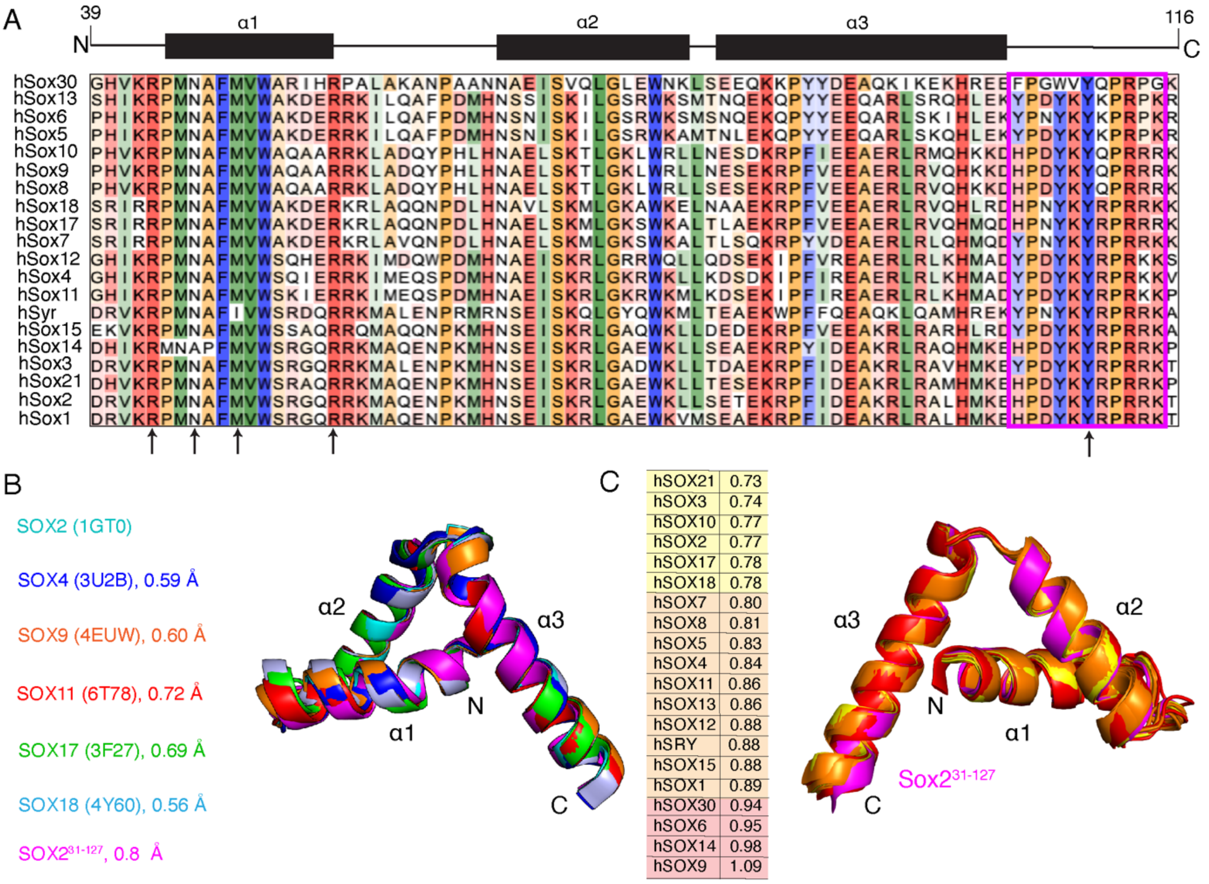

Figure S21. Comparison of Sox sequences and structures. (A) Clustal-based multiple sequence alignment of the 20 Sox family members, spanning residues D39 to T116. Residue conservation is color-coded according to Clustal conventions, with secondary structure information displayed above the alignment. Specific DNA binding interface residues are marked by black arrows. The magenta box highlights the early-middle C-terminal region which rigidifies upon DNA binding. (B) Structural superimposition of DNA-bound states of various Sox HMG domains (residues 47-104) from available PDB entries and the structure of Sox2$^{31\text{-}127}$. Color coding indicated in the Figure, with corresponding RMSD calculated against 1GT0. Minor structural deviations are observed primarily at the C-terminal of α-helix 1 and α-helix 3. (C) Color coded overlay of Sox proteins according to their RMSD value respect to the Sox2$^{31\text{-}127}$ structure.

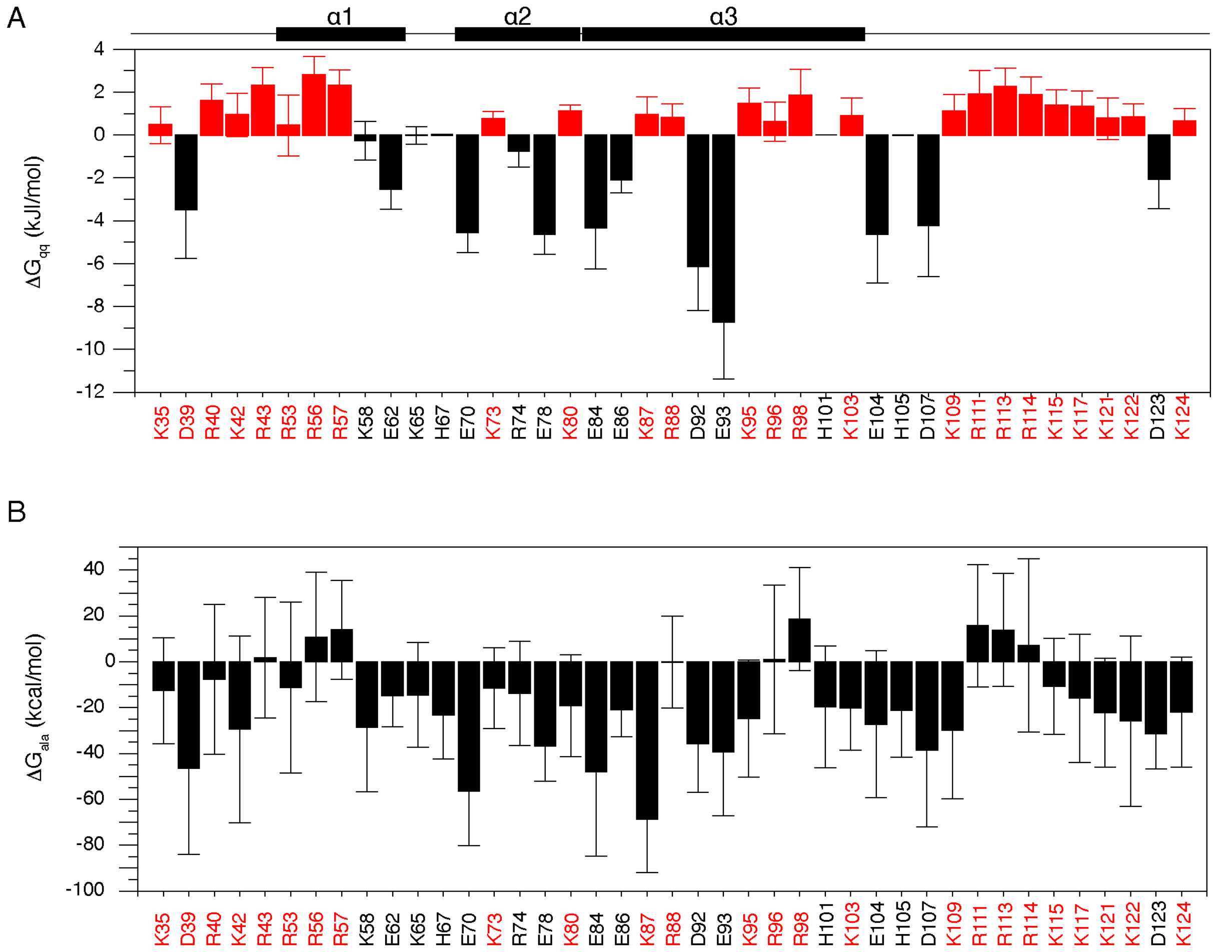

Figure S22. Computational analysis of electrostatic stability of Sox2$^{31\text{-}127}$. (A) Charge–charge interaction energy ΔG_{qq} calculated by Tanford-Kirkwood-Solvent-Accessibility Monte Carlo (TKSA-MC) model for each ionizable residue. Average value and standard deviation of ΔG_{qq} across 20 models in the NMR ensemble are given. Residues with $\Delta G_{qq} > 0$ indicating unfavorable electrostatics are colored in red. (B) Change in electrostatic energy upon Ala-mutation, evaluated after refinement in Haddock3 [6]. The average values and standard deviations from the 20 models in the NMR ensemble are plotted. The deltas are calculated by subtracting the values of the mutant from those of the wild-type. Therefore negative delta means a more negative term (lower energy) for the wild type.

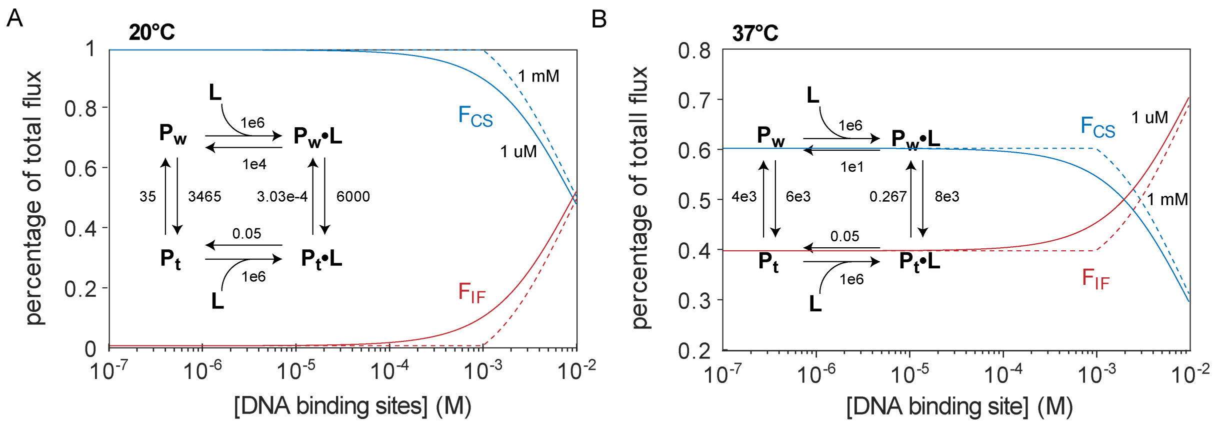

Figure S23. Contribution of conformational selection pathway to Sox2-DBD DNA binding. Assuming a simple thermodynamic square model, using the flux analysis of Hammes et al. [7], at 20 °C (A) and 37 °C (B). Nomenclature as in Hammes et al. [7], estimated kinetic rate constants indicated in the reaction scheme. Rates for the P_w/P_t equilibrium at 20 °C are derived from the experimental exchange rate for group I based on the CPMG measurements, at rates for 37 °C were set to ca. 2x higher values. Estimates for the P_t/L equilibrium are based on kinetic constants reported in Hemphill et al. [8]. The backward conformational change rate when bound to ligand was adjusted based on the other rates to ensure overall microscopic balance. For panel A the P_w state was assumed to be unfolded with very low affinity to the DNA (100 mM KD), this favors the CS mechanism. For panel B the P_w state was assumed to be partially unfolded with medium-low affinity to DNA (10 uM KD), this increases the IF contribution. The rates for the conformational change in presence of ligand were chosen to reflect stabilization of folded state compared to apo conditions. Similar results were obtained over a range of kinetic parameters. As long as the binding on-rate for the tight complex is equal or higher than that for the weak complex and the conformational change for the apo protein is relatively fast, the contribution of the conformational selection pathway is dominant for a wide range of Sox2 and DNA concentrations. Steady-state concentrations were determined analytical solutions derived from Hammes et al. [7] and subsequently used to calculate the flux through each pathway in GNU Octave.

Table S1. Buffers used for condition screening and determined melting temperatures.

Name	Organic buffer / salts	Inorganic salts	Additives	Reducents	pH	T_m (K)[a]	Ref
**1**	20 mM HEPES	150 mM NaCl	1mM EDTA 0.02 % NaN_3	1 mM DTT	7.5	312.0	https://doi.org/10.1074/jbc.M112.352864
**2**	75 mM KH_2PO_4		0.02 % NaN_3	0.5 mM DTT	6.2	310.5	https://doi.org/10.1110/ps.32801
**3**		30 mM KH_2PO_4	0.02 % NaN_3	0.5 mM DTT	5.4	302.7	https://doi.org/10.1110/ps.32801
**4**		10 mM NaH_2PO_4	0.02 % NaN_3	10 mM DTT	6.5	308.2	https://doi.org/10.1038/s41467-020-15571-8
**5**	20 mM PIPES	150 mM NaCl	0.02 % NaN_3	1 mM DTT	7	310.9	https://doi.org/10.1074/jbc.M112.352864
**6**		50 mM KH_2PO_4 150 mM NaCl	0.02 % NaN_3	1 mM DTT	5.5	313.7	https://doi.org/10.1093/nar/gku1390
**7**	50 mM sodium citrate	200 mM NaCl	0.02 % NaN_3	1 mM DTT	5.5	314.0	https://doi.org/10.1007/s10858-016-0027-z
**8**	50 mM Arg/Glu	50 mM NaH_2PO_4 100 mM NaCl	0.02 % NaN_3	0.5 mM BME	7.5	313.5	
**9.1**	20 mM Tris	135 mM KCl, 15 mM NaCl	0.02 % NaN_3	1 mM DTT	7.3	311.8	
**9.2**	20 mM Tris	135 mM KCl. 15 mM NaCl	0.02 % NaN_3	1 mM DTT	8.0	311.9	
**9.3**	20 mM Tris	135 mM KCl, 15 mM NaCl	0.02 % NaN_3	1 mM DTT	8.5	311.7	
**9.4**	20 mM Tris	50 mM KCl	0.02 % NaN_3	1 mM DTT	7.3	308.2	
**9.5**	20 mM Tris	100 mM KCl	0.02 % NaN_3	1 mM DTT	7.3	309.9	
**9.6**	20 mM Tris	250 mM KCl	0.02 % NaN_3	1 mM DTT	7.3	312.9	
**9.7**	20 mM Tris	150 mM KCl	0.02 % NaN_3	1 mM DTT	7.3	311.0	
**9.8**	20 mM Tris	150 mM NaCl	0.02 % NaN_3	1 mM DTT	7.3	311.5	
**9.9**	20 mM PIPES	135 mM KCl, 15 mM NaCl	0.02 % NaN_3	1 mM DTT	7.0	312.0	
**9.10**	20 mM PIPES	135 mM KCl, 15 mM NaCl	0.02 % NaN_3	1 mM DTT	6.5	310.9	

[a] uncertainty in T_m values is estimated to be 0.3 K based on independent replicate experiments for buffer 9.1

Table S2. Model-Free parameters for the free Sox2$^{31-127}$.

**Global diffusion tensor parameters**						
X^2	τ_m **(ns)**	D_{ratio}	**θ**	**ϕ**		
16.90	9.25	1.12	94.48	-26.97		
**Residue-specific local motion parameters**						
**Residue**	**Model**	S^2	S_{2f}	τ_e **(ps)**	R_{ex} **(s^{-1})**	**Γ**
G31	N.A[a]					
S32	N.A[a]					
N33	N.A[a]					
Q34	S/N[b]					
K35	S/N[b]					
N36	S/N[b]					
S37	S/N[b]					
P38						
D39	5	0.116±0.029	0.859±0.054	1037±40		0
R40	5	0.147±0.024	0.769±0.040	1163.5±60		0
V41	5	0.244±0.028	0.865±0.037	1139.4±44		0
K42	5	0.233±0.029	0.802±0.034	1185±72		0
R43	5	0.359±0.028	0.847±0.033	1078±81		0
P44						
M45	5	0.609±0.043	0.938±0.036	1172±142.1		0
N46	overlap					
A47	2	0.887±0.048		359±702.3		0.22E-01
F48	1	0.927±0.020				0.13E+00
M49	1	0.983±0.020				0.21E+01
V50	5	0.912±0.035	0.994±0.024	484±349		0
W51	5	0.883±0.044	0.989±0.031	473±345		0
S52	4	0.930±0.040		17±68	1.32±0.7	0

R53	4	0.916±0.030		143±82	1.4±0.62	0
G54	1	0.894±0.044				0.81E+00
Q55	4	0.925±0.029		242±211	0.5±0.52	0
R56	4	0.956±0.031		103±112	0.63±0.57	0
R57	1	0.991±0.022				0.32E+01
K58	2	0.914±0.016		280.3±229		0.33E+00
M59	4	0.895±0.027		134±57	2.03±0.56	0
A60	4	0.926±0.016		317.3±324.5	1.8±0.46	0
Q61	1	0.931±0.019				0.16E+01
E62	4	0.830±0.027		107±28	3±0.55	0
N63	4	0.807±0.033		96±26	3.1±0.71	0
P64						
K65	5	0.869±0.038	0.938±0.028	398±545		0
M66	5	0.750±0.029	0.939±0.022	1117±172		0
H67	4	0.935±0.042		172.4 ±201	0.93±0.81	0
N68	N.A[a]					
S69	N.A[a]					
E70	5	0.827±0.042	0.970±0.032	1133±340		0
I71	5	0.746±0.034	0.932±0.027	1672±418		0
S72	5	0.868±0.039	0.995±0.028	1007.3±295.3		0
K73	5	0.822±0.035	0.956±0.027	1460±449.4		0
R74	N.M[c]					
L75	N.M[c]					
G76	1	0.998±0.020				0.25E+01
A77	4	0.899±0.043		547.1±315	0.3±0.7	0
E78	5	0.834±0.035	0.943±0.027	1470±621.4		0
W79	5	0.891±0.043	0.992±0.032	1228.2±652		0
K80	4	0.924±0.030		30.4±41.5	3±0.7	0
L81	4	0.909±0.042		879±327	0.7±0.63	0
L82	1	0.892±0.019				0.25E+01
S83	4	0.836±0.027		130.1±37	1.8±0.58	0
E84	N.M[c]					

T85	5	0.818±0.047	0.892±0.033	1009.1±604		0
E86	5	0.839±0.032	0.946±0.022	686.2±242		0
K87	4	0.946±0.033		1.5±83	2.22±0.7	0
R88	2	0.915		204.4±123		0.19E+00
P89						
F90	1	0.977±0.020				0.17E+00
I91	1	0.884±0.019				0.24E+01
D92	4	0.863±0.033		12±32.3	4±0.63	0
E93	overlap					
A94	4	0.946±0.030		124±103.4	0.60±0.6	0
K95	4	0.849±0.031		35±23	2.33±0.6	0.89E-1
R96	overlap					
L97	4	0.888±0.025		295±289	2.2±0.6	0
R98	4	0.894±0.033		134.2±70	0.2±0.75	0
A99	4	0.835±0.028		91±24.3	1.7±0.54	0
L100	4	0.864±0.030		59.5±24.5	1.49±0.58	0
H101	4	0.844±0.033		134.2±49	1.94±0.68	0
M102	4	0.733±0.085		754.3±238.1	2.21±1.09	0
K103	5	0.859±0.049	0.976±0.043	344.1±512.3		0
E104	5	0.753±0.039	0.979±0.030	705.4±136		0
H105	4	0.674±0.104		911.5±241.1	0.75±1.2	0
P106						
D107	5	0.536±0.056	0.959±0.052	840.2±110.1		0
Y108	5	0.345±0.031	0.836±0.035	1362.1±90.4		0
K109	overlap					
Y110	5	0.370±0.041	0.924±0.046	1087±66.3		0
R111	5	0.301±0.040	0.874±0.047	1132±75		0
P112						
R113	S/N[b]					
R114	N.A[a]					
K115	S/N[b]					
T116	S/N[b]					

K117	S/N[b]					
T118	S/N[b]					
L119	5	0.089±0.023	0.765±0.043	993±42		0
M120	5	0.087±0.034	0.787±0.071	945±43		0
K121	5	0.046±0.034	0.795±0.061	1109±68		0
K122	5	0.077±0.031	0.821±0.048	999±38		0
D123	5	0.026±0.020	0.761±0.042	1004±26		0
K124	5	0.060±0.024	0.745±0.042	985±28.5		0
Y125	5	0.059±0.024	0.750±0.048	925.1±26.4		0
T126	5	0.052±0.019	0.708±0.042	804.4±21		0
L127	5	0.014±0.006	0.623±0.016	770±7		0

[a] not assigned

[b] too low signal-to-noise

[c] inconsistent data, could not be fitted to any of the models in ModelFree4

Table S3. Model-Free parameters for the DNA bound Sox2[31-127]

X^2	τ_m (ns)	D_{ratio}	θ	ϕ		
46.78	14.16	0.93	-65.55	53.12		
**Residue-specific local motion parameters**						
**Residue**	**Model**	S^2	S_{2f}	τ_e (ps)	R_{ex} (s^{-1})	Γ
G31	N.A[a]					
S32	N.A[a]					
N33	N.A[a]					
Q34	5	0.112±0.023	0.722±0.071	789.1±22.64		0
K35	5	0.112±0.025	0.799±0.080	924.75±28.06		0
N36	5	0.134±0.027	0.732±0.063	1022.56±50.57		0
S37	N.A[a]					
P38						
D39	5	0.264±0.034	0.823±0.057	1122.12±39.31		0
R40	5	0.356±0.042	0.829±0.056	1128.82±59.50		0
V41	5	0.604±0.045	0.859±0.041	1093±149.80		0
K42	S/N[b]					
R43	overlap					
P44						
M45	overlap					
N46	overlap					
A47	1	0.904±0.048				0.3649E+01
F48	1	0.864±0.035				0.1864E+01
M49	2	0.915±0.023		17.26±21.42		0.8780E+00
V50	1	0.902±0.037				0.2707E+01
W51	S/N[b]					
S52	S/N[b]					
R53	2	0.967±0.021		259.67±994.36		0.9544E-01
G54	N.M[c]					
Q55	2	0.923±0.040		153.75±162.47		0.6413E-01
R56	1	0.932±0.033				0.1778E+01
R57	1	0.870±0.039				0.3262E+01

K58	1	0.871±0.030				0.8881E+00
M59	2	0.906±0.028		89.91±44.90		0.1856E+00
A60	2	0.912±0.036		83.86±55.15		0.7675E+00
Q61	1	0.859±0.024				0.1056E+01
E62	1	0.863±0.024				0.6596E+00
N63	5	0.724±0.026	0.809±0.022	1558.43±559.36		0
P64						
K65	5	0.778±0.050	0.879±0.040	1199.72±433.89		0
M66	1	0.937±0.026				0.5693E+00
H67	S/N[b]					
N68	N.A[a]					
S69	N.A[a]					
E70	4	0.946±0.062		1033.37±1102.60	3.716 ±1.362	0
I71	2	0.917±0.030		49.9±31.34		0.2334E+00
S72	S/N[b]					
K73	1	0.976±0.048				0.4613E+00
R74	2	0.901±0.029		41.62±23.63		0.2008E-01
L75	S/N[b]					
G76	N.M[c]					
A77	1	0.953±0.027				0.4779E+00
E78	1	0.870±0.029				0.3668E+00
W79	1	0.990±0.042				0.2621E+00
K80	1	0.918±0.039				0.5619E+00
L81	5	0.868±0.039	0.928±0.031	644.1±407.29		0
L82	4	0.854±0.052		21.18±17.07	2.179 ±1.205	0
S83	1	0.889±0.034				0.2623E+00
E84	N.M[c]					
T85	1	0.890±0.025				0.4175E+01
E86	1	0.896±0.033				0.2902E+01

K87	4	0.857±0.067		61.22±38.40	1.478 ±1.445	0
R88	1	0.962±0.027				0.1106E+01
P89						
F90	1	0.945±0.033				0.3210E+01
I91	S/N[b]					
D92	1	0.935±0.033				0.6257E+00
E93	1	0.895±0.038				0.1150E+01
A94	overlap					
K95	5	0.857±0.033	0.929±0.027	973.55±385.06		0
R96	2	0.945±0.029		233.01±476.90		0.2658E+00
L97	1	0.963±0.049				0.2088E+01
R98	1	0.869±0.029				0.1010E+01
A99	N.M[c]					
L100	overlap					
H101	overlap					
M102	3	0.873±0.072			2.267±2.246	0.7507E-04
K103	1	0.955±0.037				0.2026E-01
E104	1	0.858±0.033				0.1440E+01
H105	2	0.853±0.032		41.38±20.12		0.1925E-02
P106						
D107	1	0.910±0.037				0.2388E+00
Y108	4	0.861±0.041		12.45±12.57	1.826±0.974	0
K109	N.M[c]					
Y110	1	0.955±0.033				0.2724E+01
R111	5	0.557±0.056	0.762±0.049	1745.82±514.90		0
P112						
R113	N.M[c]					
R114	5	0.585±0.039	0.847±0.036	1020.05±102.68		0
K115	5	0.565±0.044	0.858±0.042	1326.08±162.93		0
T116	5	0.352±0.040	0.768±0.050	1232.16±86.57		0
K117	5	0.236±0.033	0.839±0.061	1181.77±62.56		0

T118	5	0.117±0.025	0.744±0.052	1194.17±38.84		0
L119	5	0.144±0.029	0.837±0.078	1052.63±22.11		0
M120	5	0.159±0.028	0.783±0.065	1165.79±26.87		0
K121	5	0.111±0.024	0.824±0.074	1052.12±27.24		0
K122	5	0.094±0.021	0.826±0.071	1020.65±24.92		0
D123	5	0.086±0.020	0.832±0.073	966.66±16.45		0
K124	5	0.099±0.020	0.727±0.058	1165.20±33.23		0
Y125	5	0.087±0.018	0.800±0.064	1000.27±30.62		0
T126	5	0.051±0.014	0.751±0.065	853.84±21.73		0
L127	5	0.012±0.015	0.684±0.022	761.31±14.37		0.

[a] not assigned

[b] too low signal-to-noise

[c] inconsistent data, could not be fitted to any of the models in ModelFree4

Table S4. Fitted chemical shift difference (Δω)of the CPMG dispersion analysis for free state Sox2$^{31\text{-}127}$, showing group 1 residues in cyan and group 2 in orange.

**Residue (ppm)**	**Δω**
V50	3.18±0.132
W51	3.02±0.176
S52	2.96±0.173
R53	1.85±0.0777
Q55	1.48±0.0643
R57	3.39±0.170
K58	1.97±0.0813
M59	2.48±0.0763
A60	2.67±0.0781
Q61	1.93±0.066
E62	3.65±0.129
N63	3.73±0.136
K65	2.78±0.0994
H67	3.71±0.267
S72	2.69±0.0875
G76	5.42±0.276
A77	2.28±0.088
E78	1.74±0.0526
W79	1.23±0.054
K80	5.19±0.264
L81	3.62±0.145
S83	3.71±0.193
K87	5.03±0.253
R88	2.38±0.103
I91	1.79±0.113
D92	2.50±0.165

Table S5. Hydrogen-bonding analysis for H67 and H101 side chains in MD simulations of free and DNA-bound $Sox2^{31\text{-}127}$.

**Acceptor**	**Donor**	**Occupancy**	**Distance (Å)**	**Angle (°)**
Free				
H67-ND1	S69-NH	0.805	3.038	156.8
H67-ND1	S69-OH(γ)	0.191	2.860	160.9
DNA-bound				
H67-ND1	S69-NH	0.604	3.053	159.1
H67-ND1	S69-OH(γ)	0.217	2.850	159.4
DA-N3	H67-NE2HE2	0.108	2.979	139.7
K42-O	H101-NE2HE2	0.391	2.914	144.6

Supplementary References

1. Shen,Y. and Bax,A. (2013) Protein backbone and sidechain torsion angles predicted from NMR chemical shifts using artificial neural networks. *J. Biomol. NMR*, **56**, 227–241.

2. Reményi,A., Lins,K., Nissen,L.J., Reinbold,R., Schöler,H.R. and Wilmanns,M. (2003) Crystal structure of a POU/HMG/DNA ternary complex suggests differential assembly of Oct4 and Sox2 on two enhancers. *Genes Dev.*, **17**, 2048–2059.

3. Klukowski,P., Riek,R. and Güntert,P. (2022) Rapid protein assignments and structures from raw NMR spectra with the deep learning technique ARTINA. *Nat. Commun.*, **13**, 6151.

4. Pelton, J.G., Torchia, D.A., Meadow, N.D., Roseman S. (1993) Tautomeric states of the active-site histidines of phosphorylated and unphosphorylated IIIGlc, a signal-transducing protein from Escherichia coli, using two-dimensional heteronuclear NMR techniques. *Protein Sci.*, **2**, 543-58.

5. Williams,D.C., Cai,M. and Clore,G.M. (2004) Molecular Basis for Synergistic Transcriptional Activation by Oct1 and Sox2 Revealed from the Solution Structure of the 42-kDa Oct1·Sox2·Hoxb1-DNA Ternary Transcription Factor Complex *. *J. Biol. Chem.*, **279**, 1449–1457.

6. Giulini, M., Reys, V., Teixeira, J.M.C., Jiménez-García, B., Honorato, R.V., Kravchenko, A., Xu, X., Versini, R., Engel, A., Verhoeven, S. and Bonvin A.M.J.J. (2025) HADDOCK3: A modular and versatile platform for integrative modelling of biomolecular complexes. *BioRXiv*. 10.1101/2025.04.30.651432.

7. Hammes,G.G., Chang,Y.-C. and Oas,T.G. (2009) Conformational selection or induced fit: A flux description of reaction mechanism. *Proceedings of the National Academy of Sciences*, **106**, 13737–13741.

8. Hemphill, W.O., Steiner, H.R., Kominsky, J.R., Wuttke, D.S., Cech, T.R. (2024) Transcription factors ERα and Sox2 have differing multiphasic DNA- and RNA-binding mechanisms. *RNA*, **30**, 1089-1105.
